# Low Carbohydrate versus Isoenergetic Balanced Diets for Reducing Weight and Cardiovascular Risk: A Systematic Review and Meta-Analysis

**DOI:** 10.1371/journal.pone.0100652

**Published:** 2014-07-09

**Authors:** Celeste E. Naude, Anel Schoonees, Marjanne Senekal, Taryn Young, Paul Garner, Jimmy Volmink

**Affiliations:** 1 Centre for Evidence-based Health Care, Faculty of Medicine and Health Sciences, Stellenbosch University, Cape Town, South Africa; 2 Division of Human Nutrition, Department of Human Biology, Faculty of Health Sciences, University of Cape Town, Cape Town, South Africa; 3 South African Cochrane Centre, South African Medical Research Council, Cape Town, South Africa; 4 Effective Health Care Research Consortium, Department of Clinical Sciences, Liverpool School of Tropical Medicine, Liverpool, United Kingdom; University of Ottawa, Canada

## Abstract

**Background:**

Some popular weight loss diets restricting carbohydrates (CHO) claim to be more effective, and have additional health benefits in preventing cardiovascular disease compared to balanced weight loss diets.

**Methods and Findings:**

We compared the effects of low CHO and isoenergetic balanced weight loss diets in overweight and obese adults assessed in randomised controlled trials (minimum follow-up of 12 weeks), and summarised the effects on weight, as well as cardiovascular and diabetes risk. Dietary criteria were derived from existing macronutrient recommendations. We searched Medline, EMBASE and CENTRAL (19 March 2014). Analysis was stratified by outcomes at 3–6 months and 1–2 years, and participants with diabetes were analysed separately. We evaluated dietary adherence and used GRADE to assess the quality of evidence. We calculated mean differences (MD) and performed random-effects meta-analysis. Nineteen trials were included (n = 3209); 3 had adequate allocation concealment. In non-diabetic participants, our analysis showed little or no difference in mean weight loss in the two groups at 3–6 months (MD 0.74 kg, 95%CI −1.49 to 0.01 kg; I^2^ = 53%; n = 1745, 14 trials; moderate quality evidence) and 1–2 years (MD 0.48 kg, 95%CI −1.44 kg to 0.49 kg; I^2^ = 12%; n = 1025; 7 trials, moderate quality evidence). Furthermore, little or no difference was detected at 3–6 months and 1–2 years for blood pressure, LDL, HDL and total cholesterol, triglycerides and fasting blood glucose (>914 participants). In diabetic participants, findings showed a similar pattern.

**Conclusions:**

Trials show weight loss in the short-term irrespective of whether the diet is low CHO or balanced. There is probably little or no difference in weight loss and changes in cardiovascular risk factors up to two years of follow-up when overweight and obese adults, with or without type 2 diabetes, are randomised to low CHO diets and isoenergetic balanced weight loss diets.

## Background

Overweight, obesity and the related burdens of cardiovascular disease (CVD), type 2 diabetes, other non-communicable diseases (NCD) and premature mortality are escalating globally [1]–[3]. Nearly 80% of annual NCD deaths occur in low and middle income populations [4] and the NCD burden is projected to rise disproportionately in these populations over the next ten years [5].

Some weight loss diets widely promoted through the media, such as the Atkins diet [6], [7], recommend a regimen greatly restricting carbohydrates (CHO), with increased protein and unrestricted total and saturated fat intake. Advocates claim these diets are more effective for losing weight compared to balanced weight loss diets and also improve cardiovascular health, and prevent or cure diabetes [8]. To achieve the very low CHO intake, these diets prescribe restriction of most vegetables and fruit, wholegrains, legumes and other carbohydrate-containing foods. It is plausible that these low CHO diets could be harmful, especially over the longer term [9]–[11]. We therefore sought to determine whether low CHO diets have any beneficial or harmful effects on weight and cardiovascular risk factors when compared to balanced diets.

### What do existing systematic reviews say?

We first examined evidence from existing systematic reviews. We sought any review that synthesised evidence on dietary macronutrient manipulation and cardiovascular outcomes or risk factors (last search: 3 March 2014). We found 50 reviews but these had a number of methodological constraints precluding the possibility that they could meaningfully address the question we set out to answer (see Supporting Information S1 for detailed summary). The main constraints were: they did not adequately define the macronutrient composition of treatment and control diets; the total energy intake in treatment and control diets was not considered or was different between groups; arms included additional interventions that could confound the findings, such as exercise; inclusion of non-randomised studies and studies with dissimilar follow-up periods (Table 1). In light of these shortcomings, which make interpretation of the previous reviews problematic, we carried out our own systematic review.

**Table 1:** Main limitations identified in existing systematic reviews that served as constraints to interpretation of the evidence and what we did to address them in our review.

What answering the research question requires	Why was it identified as a limitation in existing reviews?	What we did to address identified limitations in our review
Explicit definition of treatment and control diets with complete macronutrient profile	If unclear, any effects seen on weight loss and CVD risk factors cannot be attributed to a well-defined intervention diet compared to a well-defined control diet	Used explicit cut-off ranges for macronutrients for treatment and control diets; the complete macronutrient profile of intervention diets had to be available (proportions of total energy intake)
Recommended energy intake in treatment and control groups needs to be similar	If different, any effects seen on weight loss and CVD risk factors would be confounded by total energy intake	Only included isoenergetic diet comparisons
Co-interventions, such as drugs given as part of the intervention, or recommendations for exercise, need to be similar in the comparison groups	If different, any effects on CVD risk factors could be confounded by co-interventions	Only included interventions with a diet component alone, or combined interventions that were similar to prevent confounding by co-interventions
Appropriate study design for the question	Methodological heterogeneity: some reviews included both controlled and uncontrolled trials	Only included randomised controlled trials
Meaningful and comparable follow-up in trials needs to be considered	Outcomes of trials with different follow-ups were pooled; generalised conclusions about weight loss may be skewed by early changes; or follow-up may be insufficient to detect CVD risk factor changes	Only included studies with 12 weeks or more follow-up; and outcomes were grouped by defined lengths of follow-up

CVD: cardiovascular disease.

Note: see Supporting Information S1 for the critical summary of existing systematic reviews.

### Macronutrient recommendations and low carbohydrate diets

Nutrition specialists have defined “recommended, balanced diets” in terms of macronutrient composition, micronutrients and dietary quality to ensure adequate nutrition, energy balance for health and weight maintenance, and prevention of NCDs in healthy populations [12]–[15]. Recommended macronutrient ranges have been developed in the USA and Canada, Australia and New Zealand and Europe [12]–[15] and are very similar across the various countries and regions. For CHO, the recommended range varies between 45 and 65% of total energy, for protein between 10 and 35% and for fat between 20 and 35%. Since not only quantity (% contribution to total energy intake), but also quality (type and nature) of macronutrients are important, guidance on the quality aspects of CHO and fats are also included in most recommendations [12]–[15]. Balanced weight loss diets restrict total energy and adhere to the principles of a balance between energy derived from CHO, protein and fat, as well as the recommended quality of each macronutrient.

To further improve our understanding of these diets, we examined and summarised the main themes in the advocacy literature on low CHO diets and their supposed benefits. We identified two main *variants* of low CHO diets. In Table 2, we summarise examples of these, along with a balanced weight loss diet, comparing key characteristics. Essentially, low CHO diets emphasise a change in recommended macronutrient balance with CHO restriction implemented by elimination or reduction of specific foods and food groups and replacement of these with high fat and protein foods. All restrict CHO intake, but the definitions used for ‘low’ and the specific implementation, advice and health claims provided with these diets vary. Very low CHO diets advocate extreme restriction of CHO and are consequently high in *both* protein and fat (which we have labelled *high fat variant*). A second variant is also high in protein, but the amount of fat is within recommended ranges and therefore restriction of CHO is less extreme (labelled *high protein variant*). This information helped to inform the protocol, specifically the sub-group analysis, for our systematic review of relevant randomised controlled trials.

**Table 2:** Low carbohydrate (CHO) diets compared with a recommended, balanced weight loss diet.

	Low CHO diet, high fat variant^a^	Low CHO diet, high protein variant^b^	Balanced weight loss diet
*Examples*	*Atkins diet* [6], [7]	*Zone diet* [67], [68]	*British Dietetic Association weight loss plan* [69]
**Energy**			
Is energy explicitly restricted?	No	No^c^	Yes
**Macronutrients**			
CHO	Extreme restriction	Moderate restriction	45–65% of total energy
Fat	Unrestricted fat	25–35% of total energy	25–35% of total energy
Protein	Unrestricted protein	Promotes lean protein	10–20% of total energy
**Quality**			
CHO	Extreme restriction of all CHO food sources	Extreme restriction of grains and starches; fruit and vegetables recommended	High fibre, unprocessed; promotion of fruit, vegetables and legumes
Protein	Unrestricted, especially animal protein	Increased lean animal protein, protein bars and shakes	Emphasis on plant protein and lean animal protein
Fat	Promotion of increased ‘natural’ fats, including saturated (animal) fats	Promotion of monounsaturated fats, mention of omega-3 fats	Promotion of polyunsaturated and monounsaturated fats, replacement of saturated fats with unsaturated fats, avoidance of trans fats; adequate omega-3 fats
**Micronutrients**			
Is micronutrient intake addressed?	Not specifically^d^	Not specifically^e^	Not specifically^f^
**Selected claimed health benefits**			
Main	Weight loss	Weight loss	Weight loss (if energy is restricted)
Other	“Improvement in risk factors for heart disease, hypertension and diabetes, inflammation”	“Reverses cellular inflammation”. “Cellular inflammation is what makes us gain weight, accelerate the development of chronic disease, and decrease our physical performance”	Reduces risk of obesity-related illness; Reduces risk of non-communicable diseases; Promotes nutritional adequacy

a Energy reduction is implicit as a consequence of extreme restriction of carbohydrates, the reported satiating effect of protein, and appetite suppressing effect of ketones.

b Energy reduction is implicit as a consequence of extreme restriction of grains and starches and reported satiating effect of protein.

c Portion guides sometimes provided.

d Potential risks of inadequacies by extreme restriction of carbohydrates, including most vegetables and fruit.

e Potential risks of inadequacies by restricting grains and starches.

f Promoted indirectly through recommending a variety of foods from all food groups and quality food choices (including plenty of vegetables and fruit).

## Objective

To compare the effects of low CHO and isoenergetic balanced weight loss diets in overweight and obese adults.

## Inclusion Criteria

### Types of studies

We included randomised controlled trials (RCTs) in humans published in English. Trials could be of a parallel or crossover design, however, crossover trials were only included if first period data could be extracted. We excluded trials with less than 10 participants randomised in each group.

### Types of participants

People who are overweight or obese, have diabetes, glucose intolerance or insulin resistance, cardiovascular conditions or risk factors such as hypertension and dyslipidaemia, as defined by trial authors. We excluded pregnant and lactating women and individuals younger than 18 years.

### Types of interventions

We required the studies to provide the macronutrient goals of the diet in terms of their contribution to total energy intake, or that these goals could be calculated as proportions of total energy intake, for both the treatment and comparison arms. Treatment diets were low CHO weight loss diet plans, including a) low CHO, high fat, high protein diet (*high fat variant*) or b) low CHO, recommended fat, high protein diet (*high protein variant*) (Table 3). Control diets were balanced weight loss diet plans (Table 3) with the same or similar prescribed energy content as the treatment diet.

**Table 3:** Cut-off ranges* used to classify the macronutrient goals of treatment and control diets.

	Classifications		
Macronutrients	Low	Balanced	High
Carbohydrate (% of total energy)	<45	45 to 65	>65
Fat (% of total energy)	<25	25 to 35	>35
Protein (% of total energy)	<10	10 to 20	>20

*Established by drawing on macronutrient recommendations from five global institutions and governments [12]–[15], [70].

We excluded studies where: the treatment and control diets were not adequately defined or where the control diet was defined as ‘no dietary intervention’; diets were combined with any other interventions (e.g. exercise, pharmacological, surgical) so that the effect of diet alone could not be assessed; dietary interventions had an exclusive focus on energy restriction, i.e. no macronutrient manipulation was instituted; a substantial disparity in energy intake (>500 kilojoules) between the prescribed treatment and control diets was present; an *ad libitum* energy prescription was used; interventions focused on specific foods, food groups or food components (e.g. dairy, oats, plant sterols), meal replacement or supplement products were used; the duration of the intervention was less than 12 weeks or test meal responses (post-prandial) were assessed.

### Types of outcome measures

#### Weight

Total weight change (kg); body mass index (BMI) (kg/m^2^).

#### Markers of cardiovascular disease risk

Diastolic blood pressure (DBP) and systolic blood pressure (SBP) (mmHg); serum cholesterol: low density lipoprotein (LDL), high density lipoprotein (HDL) and total (mmol/L); serum triglycerides (TG) (mmol/L).

#### Markers of diabetes mellitus risk or glycaemic control

Glycosylated haemoglobin (HbA1c) (%); fasting blood glucose (FBG) (mmol/L).

Mortality, myocardial infarction and stroke were not explicitly excluded as outcomes, but we did not expect to find randomised controlled trials with these outcomes where dietary manipulations were under study.

## Search Methods for Identification of Studies

Electronic searches were done in *MEDLINE* via *PubMed*, Excerpta Medica Database (EMBASE) and *The Cochrane Central Register of Clinical Trials* (CENTRAL), with the last search on 19 March 2014. The full electronic search strategy for EMBASE is detailed in Table 4. In addition, the references of the previously mentioned 50 existing systematic reviews were searched.

**Table 4:** Search strategies for EMBASE.

Search: 22 October 2012		
No.	Query	Results
#5	**#3** AND **#4**	1312
#4	‘randomised controlled trial’/exp OR ‘randomised controlled trial’ OR ‘randomised controlled trials’ OR ‘randomized controlled trial’/exp OR ‘randomized controlled trial’ OR ‘randomized controlled trials’/exp OR ‘randomized controlled trials’ AND [humans]/lim AND [english]/lim AND [embase]/lim AND [1-1-1966]/sd NOT [22-10-2012]/sd AND [1966-2012]/py	249285
#3	**#1** AND **#2**	2862
#2	‘carbohydrate restricted diet’/exp OR ‘carbohydrate restricted diet’ OR ‘carbohydrate restricted diets’ OR ‘high fat diet’/exp OR ‘high fat diet’ OR ‘high fat diets’ OR ‘fat restricted diet’/exp OR ‘fat restricted diet’ OR ‘fat restricted diets’ OR ‘ketogenic diet’/exp OR ‘ketogenic diet’ OR ‘ketogenic diets’ AND [humans]/lim AND [english]/lim AND [embase]/lim AND [1-1-1966]/sd NOT [22-10-2012]/sd AND [1966–2012]/py	11176
#1	‘randomized controlled trial’/exp OR ‘randomized controlled trial’ OR random*:ab,ti OR trial:ti OR allocat*:ab,ti OR factorial*:ab,ti OR placebo*:ab,ti OR assign*:ab,ti OR volunteer*:ab,ti OR ‘crossover procedure’/exp OR ‘crossover procedure’ OR ‘double-blind procedure’/exp OR ‘double-blind procedure’ OR ‘single-blind procedure’/exp OR ‘single-blind procedure’ OR (doubl* NEAR/3 blind*):ab,ti OR (singl*:ab,ti AND blind*:ab,ti) OR crossover*:ab,ti OR cross+over*:ab,ti OR (cross NEXT/1 over*):ab,ti AND [humans]/lim AND [english]/lim AND [embase]/lim AND [1-1-1966]/sd NOT [22-10-2012]/sd AND [1966–2012]/py	879594

Updated search: 5 June 2013		
#5	**#3** AND **#4**	80
#4	‘randomised controlled trial’/exp OR ‘randomised controlled trials’ OR ‘randomized controlled trial’/exp OR ‘randomized controlled trials’/exp AND [humans]/lim AND [english]/lim AND [embase]/lim AND [23-10-2012]/sd NOT [6-6-2013]/sd	20424
#3	**#1** AND **#2**	236
#2	‘carbohydrate restricted diet’/exp OR ‘carbohydrate restricted diets’ OR ‘high fat diet’/exp OR ‘high fat diets’ OR ‘fat restricted diet’/exp OR ‘fat restricted diets’ OR ‘ketogenic diet’/exp OR ‘ketogenic diets’ AND [humans]/lim AND [english]/lim AND [embase]/lim AND [23-10-2012]/sd NOT [6-6-2013]/sd	1005
#1	‘randomized controlled trial’/exp OR random*:ab,ti OR trial:ti OR allocat*:ab,ti OR factorial*:ab,ti OR placebo*:ab,ti OR assign*:ab,ti OR volunteer*:ab,ti OR ‘crossover procedure’/exp OR ‘double-blind procedure’/exp OR ‘single-blind procedure’/exp OR (doubl* NEAR/3 blind*):ab,ti OR (singl*:ab,ti AND blind*:ab,ti) OR crossover*:ab,ti OR cross+over*:ab,ti OR (cross NEXT/1 over*):ab,ti AND [humans]/lim AND [english]/lim AND [embase]/lim AND [23-10-2012]/sd NOT [6-6-2013]/sd	73855

Updated search: 19 March 2014		
#5	#3 AND #4	145
#4	‘randomised controlled trial’/exp OR ‘randomised controlled trial’ OR ‘randomised controlled trials’ OR ‘randomized controlled trial’/exp OR ‘randomized controlled trial’ OR ‘randomized controlled trials’/exp OR ‘randomized controlled trials’ AND [humans]/lim AND [english]/lim AND [embase]/lim AND [7-6-2013]/sd NOT [18-3-2014]/sd	29989
#3	#1 AND #2	384
#2	‘carbohydrate restricted diet’/exp OR ‘carbohydrate restricted diet’ OR ‘carbohydrate restricted diets’ OR ‘high fat diet’/exp OR ‘high fat diet’ OR ‘high fat diets’ OR ‘fat restricted diet’/exp OR ‘fat restricted diet’ OR ‘fat restricted diets’ OR ‘ketogenic diet’/exp OR ‘ketogenic diet’ OR ‘ketogenic diets’ AND [humans]/lim AND [english]/lim AND [embase]/lim AND [7-6-2013]/sd NOT [18-3-2014]/sd	1731
#1	‘randomized controlled trial’/exp OR ‘randomized controlled trial’ OR random*:ab,ti OR trial:ti OR allocat*:ab,ti OR factorial*:ab,ti OR placebo*:ab,ti OR assign*:ab,ti OR volunteer*:ab,ti OR ‘crossover procedure’/exp OR ‘crossover procedure’ OR ‘double-blind procedure’/exp OR ‘double-blind procedure’ OR ‘single-blind procedure’/exp OR ‘single-blind procedure’ OR (doubl* NEAR/3 blind*):ab,ti OR (singl*:ab,ti AND blind*:ab,ti) OR crossover*:ab,ti OR cross+over*:ab,ti OR (cross NEXT/1 over*):ab,ti AND [humans]/lim AND [english]/lim AND [embase]/lim AND [7-6-2013]/sd NOT [18-3-2014]/sd	108635

## Data Collection and Analysis

### Selection of studies

Two authors (CN and AS) screened titles and abstracts of all search results and identified potentially eligible studies using the pre-specified eligibility criteria. Full text articles for these studies were obtained and assessed by the two authors simultaneously. Studies not fulfilling eligibility criteria were excluded with reasons. All discrepancies were resolved by consensus.

### Data extraction and management

Two authors (CN and AS) extracted data using an electronic data extraction spreadsheet in Microsoft Excel. The main sections of the spreadsheet included information on the design, country, participants, treatment, control, diet quality, energy and nutrient composition, adherence, outcomes and results, funding, conflict of interest, and risk of bias. The extracted data were collated in tables and figures. The author of one included RCT [16] was contacted and provided means and standard deviations that could not be read accurately from a figure in the publication.

### Length of follow-up

Outcomes were grouped into those measured between baseline and three to six months of follow-up; and between baseline and one to two years of follow-up. For trials measuring outcomes at several time points within either of these two categories, we took the values for the longest follow-up within that category (for example, where results were available at three and six months, the results at six months were used).

### Risk of bias assessment

Two authors (CN and AS) assessed the risk of bias in the included studies by using the Cochrane Collaboration risk of bias tool [17], where domains include random sequence generation, allocation concealment, performance and detection bias, attrition bias, reporting bias and ‘other’ bias. Criteria for low risk, high risk and unclear risk of bias per the Cochrane Handbook for Systematic Reviews of Interventions [17] were used.

### Adherence

For energy, the prescribed and reported total energy intakes (kilojoules) for each reported follow-up category in the trial were tabulated per group, as were group comparisons of mean reported energy intake reported by trial authors. For macronutrients, adherence was calculated as the difference between the reported mean and prescribed distribution of energy intake (% of total energy) from CHO, fat and protein for each follow-up category. For trials reporting dietary intake at several time points within either of the two follow-up categories, we took the values for the longest follow-up within that category. Specifically, adherence was calculated using a Mahalanobis distance equation, which can be used to measure the similarity between a set of actual conditions relative to a set of ideal conditions [18]. The equation generated an adherence score that represents the degree of deviation from the prescribed goals for macronutrients in the treatment and control groups. A lower score reflects better adherence and a higher score reflects poorer adherence.

The equation for the macronutrient adherence score, where TE is total energy:
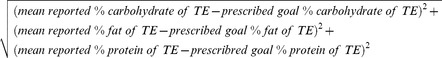



### Measures of treatment effect

Review Manager (RevMan) 5.2 was used to manage the extracted data and to conduct meta-analyses [19] for each outcome, where relevant, to determine a pooled effect of low CHO diets compared to balanced diets. Mean differences (MD) were calculated for continuous data and reported alongside 95% confidence intervals (CIs). Where change per group was not available, end values were used and we combined change from baseline results with end values [17]. Footnotes on the figures of forest plots indicate when end values were used.

### Unit of analysis issues

No crossover trials met the inclusion criteria. In the case of multiple intervention groups, we selected one pair of interventions i.e. treatment and control that was most relevant to this systematic review question [17].

### Assessment of heterogeneity

Statistical heterogeneity was assessed with the Chi^2^ test (significance level p <0.1) and quantified with the I^2^ test [20] where I^2^ values of 50% or more indicate a substantial level of heterogeneity and values of 75% or more indicate considerable heterogeneity [17].

### Assessment of reporting bias

We assessed reporting bias with funnel plots when we had 10 or more studies per outcome, which was the case for five outcomes in non-diabetic overweight and obese adults in the early follow-up category.

### Data synthesis and investigation of heterogeneity

The outcomes were reported as the difference in the mean change between the treatment and control groups. Because the presence of diabetes is likely to influence the effects of the diet, we stratified by trials of overweight and obese participants without and with type 2 diabetes. Heterogeneity between the included studies was anticipated due to variations in dietary plans and goals, length of follow-up and dietary methodology, and the random-effects model was therefore used for all meta-analyses. We stratified the analysis by whether the treatment group was the high fat variant or the high protein variant of low CHO diets, and pooled the estimate if there was no obvious heterogeneity.

### GRADE analysis

We assessed the quality of evidence using GradePro (Grade Profiler) 3.2.2 software [21], [22]. We used standard terms to translate the quality of the evidence, as assessed by GRADE, into words to express the quality of evidence and magnitude of effect. For example, for large effects and moderate quality evidence, we use the word “probably”, whereas for low quality we use the word “may” [23].

## Results

### Description of studies

#### Results of the search and included studies

**Figure 1:**
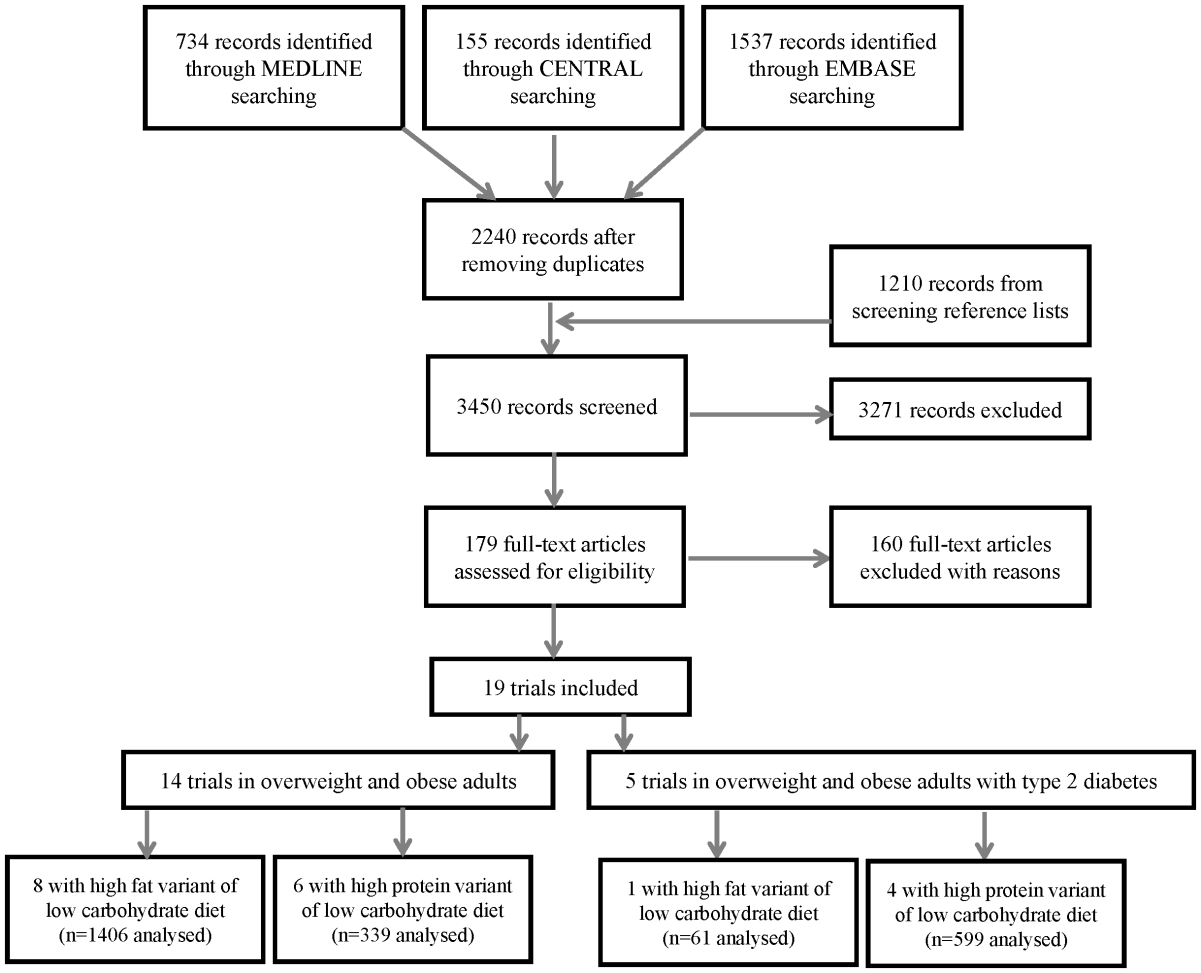
Flow diagram illustrating the search results and selection process, as well as the variants of the low carbohydrate diets used as treatments in the included trials. The high fat variant of low carbohydrate diets is low in carbohydrates (<45% of total energy), high in fat (>35% of total energy) and high in protein (>20% of total energy). The high protein variant of low carbohydrate diets is low in carbohydrates (<45% of total energy), has a recommended proportion of fat (20 to 35% of total energy) and is high in protein (>20% of total energy).

We screened 3450 records and retrieved and screened 179 full-text articles, after which we included 19 RCTs (Figure 1). We included 19 RCTs with 3209 participants [16], [24]–[41]. All trials used a parallel group design, were published after 2001 and were conducted in high-income countries (United States of America (5), Australia (7), New Zealand (1), Germany (1), Norway (1), United Kingdom (1), Sweden (1) and Spain (2)). Sample size varied between 25 and 402 participants. Follow-up ranged from 12 weeks to two years.

There were 14 trials in people without diabetes [16], [24], [26]–[29], [31]–[33], [36]–[39], [41] and five trials in people with type 2 diabetes mellitus [25], [30], [34], [35], [40]. Nine trials tested the high fat variant of the low CHO diet and 10 trials tested the high protein variant. Figure 1 displays the number of trials and variants of the low CHO diet used as treatments in each population. In people without diabetes, eight trials examined the high fat variant [16], [24], [26], [27], [29], [32], [33], [38] and 6 the high protein variant [28], [31], [36], [37], [39], [41]. A single trial [30] evaluated the high fat variant and four [25], [34], [35], [40] evaluated the high protein variant in adults with type 2 diabetes mellitus. No included trials reported mortality, myocardial infarction or stroke as outcomes.

Two trials were only in men [33], [41] and the rest were mixed. All trials included only participants who were overweight or obese (BMI of 26 kg/m^2^ or greater). In all trials that reported baseline BMIs, the mean baseline BMI in both groups was greater than 30 kg/m^2^. The WHO classifies an individual as overweight when their BMI is greater than or equal to 25 kg/m^2^ and as obese when BMI is greater than or equal to 30 kg/m^2^ [42]. Table 5 provides characteristics of included trials per population group and Table 6, the prescribed dietary goals for the treatment and control diets per included trial and population group. We excluded 160 full-text articles with reason given in Table 7; the most common reason was follow-up less than 12 weeks.

**Table 5:** Characteristics of included randomised controlled trials.

First author (follow-up in weeks)	Year of publication	Country	Parallel design	No randomised	No Completed in Rx group	Dropout in Rx group	No Completed in Control group	Dropout in Control group	Gender	Age Range (yrs)	Types of Participants	Total Intervention Period in weeks
**Overweight and obese adults**												
Aude (12) [24]	2004	USA	Yes	60	29	1	25	5	Both	27–71	Overweight or Obese	12
De Luis (12) [27]	2009	Spain	Yes	118	52	0	66	0	Both	NR	Overweight or Obese	12
De Luis (12) [26]	2012	Spain	Yes	305	147	0	158	0	Both	NR	Obese	12
Farnsworth (16) [28]	2003	UK	Yes	66	28	NR	29	NR	Both	20–65	Overweight or Obese	16
Frisch (52) [29]	2009	Germany	Yes	200	85	15	80	20	Both	18–70	Overweight or Obese	52
Keogh (52) [31]	2008	Australia	Yes	36	7	NR	6	NR	Both	20–65	Overweight or Obese	52
Klemsdal (52) [32]	2010	Norway	Yes	202	78	22	86	16	Both	30–65	Overweight or Obese and CVD risk	52
Krauss (12) [33]	2006	USA	Yes	224	40	12	49	8	Males	NR	Overweight or Obese with Dyslipidaemia	12
Lasker (16) [36]	2008	USA	Yes	65	25	7	25	8	Both	40–56	Overweight or Obese	16
Layman (52) [37]	2009	USA	Yes	130	41	23	30	36	Both	40–57	Overweight or Obese	52
Lim (64) [38]	2010	Australia	Yes	113	17	13	15	15	Both	20–65	Overweight or Obese with CVD risk	64
Luscombe (16) [39]	2003	Australia	Yes	36	17	0	19	0	Both	20–65	Overweight or Obese	16
Sacks (104) [16]	2009	USA	Yes	811	168	33	169	35	Both	30–70	Overweight or Obese	104
Wycherley (52) [41]	2012	Australia	Yes	123	33	26	35	29	Males	20–65	Overweight or Obese	52
**Overweight and obese adults with Type 2 diabetes mellitus**												
Guldbrand (104) [30]	2012	Sweden	Yes	61	30	0	31	0	Both	NR	Overweight or Obese with T2DM	104
Brinkworth (64) [25]	2004	Australia	Yes	66	19	14	19	14	Both	58–65	Overweight or Obese with T2DM	64
Krebs (104) [34]	2012	New Zealand	Yes	419	144	63	150	62	Both	30–78	Overweight or Obese with T2DM	104
Larsen (52) [35]	2011	Australia	Yes	108	48	9	45	6	Both	30–75	Overweight or Obese and T2DM	52
Parker(12) [40]	2002	Australia	Yes	66	26	6	28	6	Both	NR	Obese with T2DM	12

CVD = cardiovascular disease; No = number; NR = not reported; Rx = treatment; T2DM = type two diabetes mellitus; USA = United States of America; yrs = years.

Note: In the case of multiple intervention groups, we selected one pair of interventions i.e. treatment and control that was most relevant to this systematic review question.

**Table 6:** Prescribed dietary goals per length of follow-up for included randomised controlled trials.

First author (follow-up in weeks)	Year of publication	No. of weeks of weight loss	Prescribed energy for Rx group	Prescribed energy for Control group	Prescribed CHO for Rx group	Prescribed fat for Rx group	Prescribed protein for Rx group	Prescribed CHO for Control group	Prescribed fat for Control group	Prescribed protein for Control group
			(kJ)	(kJ)	(% of TE)	(% of TE)	(% of TE)	(% of TE)	(% of TE)	(% of TE)
**Overweight and obese adults: High fat variant of low CHO diet**										
Aude (12)	2004	12	5460–6720	5460–6720	28	39	33	55	30	15
De Luis (12)	2009	12	6300	6330	38	36	26	52	27	20
De Luis (12)	2012	12	6329	6300	38	36	26	53	27	20
Frisch (24 and 52)	2009	24	2100 deficit	2100 deficit	<40	>35	25	>55	<30	15
Klemsdal (24 and 52)	2010	24	2100 deficit	2100 deficit	30–35	35–40	25–30	55–60	<30	15
Krauss (12)	2006	5	4200 deficit	4200 deficit	26	45	29	54	30	16
Lim (24 and 64)	2010	24	6500	6500	4	60	35	50	30	30
Sacks (24 and 104)	2009	24	3150 deficit	3151 deficit	35	40	25	65	20	15
**Overweight and obese adults: High protein variant of low CHO diet**										
Farnsworth (16)	2003	12	6000–6300	6000–6300	40	30	30	55	30	15
Keogh (12 and 52)	2008	12	6000	6000	33	27	40	60	20	20
Lasker (16)	2008	16	7100	7100	40	30	30	55	30	15
Layman (16 and 52)	2009	16	7100–7940	7100–7940	40	30	30	55	30	15
Luscombe (16)	2003	12	6500–8200	6500–8201	40	30	30	55	30	15
Wycherley (12 and 52)	2012	52	7000	7000	40	25	35	58	25	17
**Overweight and obese adults with type 2 diabetes mellitus: High fat variant of low CHO diets**										
Guldbrand (12–24)	2012	12–24	M:6696	M:6696;	20	50	30	55–60	30	10–15
			F: 7531	F: 7531						
Guldbrand (104)	2012	104	M:6696;	M:6696;	20	50	30	55–60	30	10–15
			F: 7531	F: 7531						
**Overweight and obese adults with type 2 diabetes mellitus: High protein variant of low CHO diets**										
Brinkworth (12)	2004	8	NR	NR	40	30	30	55	30	15
Brinkworth (64)	2004	N/A	NR	NR	40	30	30	55	30	15
Krebs (24 and 104)	2012	12	2000 deficit	2000 deficit	40	30	30	55	30	15
Larsen (12)	2011	12	6400/−30%E	6400/−30%E	40	30	30	55	30	15
Larsen (52)	2011	N/A	E balance	E balance	40	30	30	55	30	15
Parker (12)	2002	8	6720-E balance	6721-E balance	40	30	30	60	25	15

CHO = carbohydrate; E = energy; F = females; g = gram; kJ = kilojoule; M = males; MJ = megajoule; N/A = not applicable; No = number; NR = not reported; Rx = treatment; TE = total energy.

**Table 7:** Excluded studies and reasons for exclusion.

Reasons for exclusion	Number of studies excluded
Not a randomised controlled trial	4 [71]–[74]
Duration of the intervention <12 weeks	40 [75]–[114]
All three macronutrients not prescribed (or cannot be calculated as proportions of the total energy intake)	20 [115]–[134]
Non-English language	1 [135]
Test meal response measured	1 [136]
Meal replacement	2 [137], [138]
Combined interventions were involved	3 [139]–[141]
Treatment and control both low carbohydrate – not an eligible comparison	3 [142]–[144]
Comparison not meaningful (carbohydrate content of treatment and controls differ <5% of TE)	2 [145], [146]
No eligible balanced carbohydrate control	1 [147]
Crossover trial where first period data cannot be extracted: 1	1 [148]
Substantial disparity in energy intake between prescribed intervention diets	13 [49], [51], [149]–[159]
Treatment diet is not low in carbohydrates	26 [160]–[185]
Control diet is not within balanced macronutrient range	4 [186]–[189]
Duplicate and/or complimentary	24 [190]–[213]
Energy intake *ad libitum*	8 [214]–[221]
Ineligible low carbohydrate diet variant	6 [222]–[227]
Less than 10 participants randomised per group	1 [228]

RCT = randomised controlled trial; CHO = carbohydrate.

#### Risk of bias in included studies

**Figure 2:**
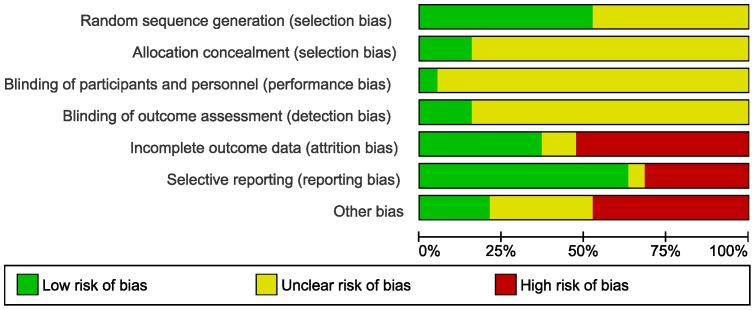
Risk of bias: systematic review authors' judgements about each risk of bias item presented as percentages across all included trials using the Cochrane risk of bias tool (n = 19).

Risk of bias is reported in Table 8 and displayed in Figure 2.

**Table 8:** Risk of bias in overweight and obese adult population.

First author	Year published	Random sequence generation Judgement	Random sequence generation Comment	Allocation concealment Judgement	Allocation concealment Comment	Performance bias Judgement	Performance bias Comment	Detection bias Judgement	Detection bias Comment	Attrition bias Judgement	Attrition* bias Comment (Rx/Control group)	Reporting bias Judgement	Reporting bias Comment	Other bias Judgement	Other bias Comment
**Overweight and obese adults**															
Aude [24]	2004	Low risk	Block design	Unclear risk	NR	Unclear risk	Equal contact time but not blinded	Low risk	Assessors blinded	High risk	3%/17% attrition (differential), no reasons	Low risk	Protocol not available, but prespecified and all NB outcomes addressed	High risk	Food choice advice & fibre supplements only given to Rx group
De Luis [27]	2009	Low risk	Random number list	Unclear risk	“closed envelope”	Unclear risk	Equal contact time but not blinded	Unclear risk	Not blinded	Low risk	No attrition	Low risk	Protocol not available, but prespecified and all NB outcomes addressed	High risk	Funding & COI NR, imbalanced baseline DBP, HDL, TG
De Luis [26]	2012	Unclear risk	NR	Unclear risk	NR	Unclear risk	Equal contact time but not blinded	Unclear risk	Not blinded	Low risk	No attrition	High risk	No prespecified outcomes, protocol not available	High risk	Funding & COI NR, imbalanced baseline SBP, HDL
Farnsworth [28]	2003	Unclear risk	NR	Unclear risk	NR	Unclear risk	Equal contact time but not blinded	Unclear risk	Not blinded	Unclear risk	14% total attrition, attrition & reasons not provided per group	High risk	No prespecified outcomes, protocol not available	Unclear risk	Funding: possible influences
Frisch [29]	2009	Low risk	Computer generated random no. lists	Unclear risk	NR	Unclear risk	Equal contact time but not blinded	Unclear risk	Not blinded	Low risk	ITT analysis	Low risk	Prespecified and all NB outcomes addressed, protocol available	Low risk	-
Keogh [31]	2008	Unclear risk	NR	Unclear risk	NR	Unclear risk	Equal contact time but not blinded	Unclear risk	Not blinded	High risk	36% total attrition, attrition & reasons not provided per group	Low risk	Prespecified and NB outcomes addressed, protocol available	High risk	Incomplete and suspected errors in reporting, imbalanced baseline TG
Klemsdal [32]	2010	Unclear risk	NR	Unclear risk	NR	Unclear risk	Equal contact time but not blinded	Unclear risk	Not blinded	Low risk	ITT analysis	Low risk	Prespecified and NB outcomes addressed, protocol available	Unclear risk	COI NR
Krauss [33]	2006	Low risk	Blocks of 4, 8, 12, 16, 20, 24	Unclear risk	Sealed sequentially no. envelopes, not opaque	Unclear risk	Equal contact time but not blinded	Unclear risk	Not blinded	High risk	23/14% attrition (differential), reasons not per group	High risk	Only 1 outcome prespecified, protocol not available	Unclear risk	COI NR
Lasker [36]	2008	Low risk	Block randomisation	Unclear risk	NR	Unclear risk	Equal contact time but not blinded	Unclear risk	Not blinded	High risk	22/24% attrition, no reasons	Low risk	Protocol not available, but prespecified and all NB outcomes addressed	High risk	Funding: possible influences
Layman [37]	2009	Unclear risk	NR	Unclear risk	NR	Unclear risk	Equal contact time but not blinded	Unclear risk	Not blinded	High risk	36/55% attrition (differential), reasons differ per group	Low risk	Protocol not available, but prespecified and all NB outcomes addressed	Unclear risk	Funding and COI reported: possible influences
Lim [38]	2010	Unclear risk	NR	Unclear risk	NR	Unclear risk	Equal contact time but not blinded	Unclear risk	Not blinded	High risk	43/50% attrition, reasons differ per group (differential)	Low risk	Protocol not available, but prespecified and all NB outcomes addressed	Low risk	-
Luscombe [39]	2003	Unclear risk	NR	Unclear risk	NR	Unclear risk	Equal contact time but not blinded	Unclear risk	Not blinded	Low risk	No attrition	High risk	No prespecified outcomes, protocol not available	Unclear risk	COI NR; Funding: possible influences
Sacks [16]	2009	Unclear risk	NR	Low risk	Centrally	Low risk	Participants blinded	Low risk	Assessors blinded	Low risk	ITT	Low risk	Prespecified and all NB outcomes addressed, protocol available	Low risk	-
Wycherley [41]	2012	Low risk	Computer generated random no. lists	Unclear risk	NR	Unclear risk	Equal contact time but not blinded	Unclear risk	Not blinded	High risk	44/45% attrition	Unclear risk	Protocol retrospectively registered, outcomes only specified in abstract, NB outcomes addressed	High risk	Funding: possible influence, analysis at 12 weeks only included data from 52 week completers but dropouts after 12 weeks lost less weight
**Overweight and obese adults with Type 2 diabetes mellitus**															
Brinkworth [25]	2004	Low risk	Random no. generator	Unclear risk	NR	Unclear risk	Equal contact time but not blinded	Unclear risk	Not blinded	High risk	42/42% attrition, reasons differ per group	Low risk	Protocol not available, but prespecified and all NB outcomes addressed	High risk	Imbalanced baseline weight, DBP, SBP glucose
Guldbrand [30]	2012	Low risk	Drawing blinded ballots	Unclear risk	NR	Unclear risk	Equal contact time but not blinded	Unclear risk	Not blinded	Low risk	No attrition	High risk	Protocol available: prespecified outcomes vague	High risk	Imbalanced baseline weight & BMI
Krebs [34]	2012	Low risk	Computer generated random no.	Low risk	Independent biostatistician	Unclear risk	Equal contact time but not blinded	Unclear risk	Not blinded	High risk	30/29%, reasons differ per group (differential)	Low risk	Prespecified and NB outcomes addressed, protocol available	Low risk	-
Larsen [35]	2011	Low risk	Random block sizes	Low risk	Centrally	Unclear risk	Equal contact time but not blinded	Low risk	Assessors blinded	High risk	16/12% attrition, reasons differ per group (differential) LOCF analysis only on some missing participants	Low risk	Prespecified and NB outcomes addressed, protocol available	Unclear risk	COI: NR
Parker [40]	2002	Unclear risk	NR	Unclear risk	NR	Unclear risk	Equal contact time but not blinded	Unclear risk	Not blinded	Unclear risk	19/18% attrition, no reasons	High risk	No prespecified outcomes, protocol not available	High risk	COI NR; Funding: possible influences; imbalanced baseline weight & glucose

*number of attrition per group given for longest follow-up within the categories;

BMI = body mass index; BP = blood pressures; COI = conflict of interest; DBP = diastolic blood pressure; HDL = high density lipoprotein cholesterol; ITT = intention-to-treat; LOCF: Last observation carried forward; NB = important; No = number; NR = Not reported; Rx = treatment; TG = triglycerides.

*Generation of sequence and allocation concealment:* Ten trials reported the method of randomisation; three trials reported adequate concealment and in the rest it was unclear.

*Blinding:* Blinding of participants in diet trials is not easy as they usually have to follow specific dietary plans in order to attain the prescribed goals of the intervention diets. Three trials blinded the outcome assessors of which one also reported blinding the participants.

*Incomplete outcome data:* After 3–6 months, reported loss to follow-up ranged from no loss to 42%, peaking at 47% after 15 months in one of the trials. Ten trials had overall attrition greater than 20%, differential attrition between groups, or both. Six trials had differential loss and/or different or unspecified reasons for loss to follow-up. Seven trials had low risk of attrition bias, with four reporting no attrition and three performing intention-to-treat analysis.

*Selective reporting:* Six trials did not have protocols available or outcomes were not pre-specified in the methods section of the trial reports and one trial had an unclear risk of reporting bias. The remaining 12 trials were judged to have low risk of reporting bias.

*Publication bias:* Assessment of the funnel plot asymmetry for the five outcomes in overweight and obese adults in the early follow-up category showed that for weight loss, small studies with a negative mean difference are missing. Similarly, smaller studies appear to be missing for the other four outcomes, namely serum LDL, HDL and total cholesterol, and serum triglycerides (data not shown).

*Other potential sources of bias:* Nine trials were judged to have a high risk of other types of bias. Six trials were funded independently, five were funded by industry, five by a combination of independent and industry funding and the remaining three trials did not report their funding source. Four trials had low risk of other bias.

#### Adherence to prescribed dietary goals

Table 9 shows the energy prescriptions, the mean reported total energy intakes and the calculated adherence scores for macronutrients for all lengths of follow-up per diet group (see Table 6 for the prescribed dietary goals for the treatment and control diets per included trial). Energy prescriptions for the weight loss diets were expressed as absolute goals or ranges, or as absolute or percentage deficits, with some trials using sex-specific goals. In the 12 trials that reported group comparisons in energy intake, only one found a difference, with a lower reported intake in the balanced diet group [34] (Table 9). None of these 12 trials demonstrated a difference in weight loss between the low CHO and balanced diet groups at any follow-up category.

**Table 9:** Group comparisons of mean reported energy intakes and calculated adherence scores per diet group for all lengths of follow-up.

Study ID	Length of follow-up (weeks)	Energy prescription in both groups in kJ	Mean reported energy intake (SD) in kJ		Group comparison of mean reported energy intake reported by trial authors	Adherence scores^a^ for macronutrients	
			Low CHO diet group	Balanced diet group		Low CHO diet group	Balanced diet group
Aude 2004	12	6720 (m); 5460 (f)	–	–	NA	–	–
Brinkworth 2004	all	equivalent	–	–	NA	–	–
De Luis 2009	12	6330	6502 (NR)	6775 (NR)	–	–	–
De Luis 2012	12	6300–6329	6598 (NR)	6779 (NR)	–	–	–
Farnsworth 2003	12	6000–6300	6300 (529)	6500 (539)	“did not differ”		
	16	balance	8000 (1058)	8200 (1077)	“did not differ”	5.93	4.00
Frisch 2009	24	2100 deficit	7316 (2621)	7489 (2507)	p = 0.636	5.96	6.13
	52		7837 (2982)	7787 (2621)	p = 0.903	7.08	5.19
Guldbrand 2012	12–24	7531 (m); 6694 (f)	5791 (1531)	6498 (1787)	p = 0.065 for change	7.87	8.54
	52		6017 (2075)	6619 (2075)	over all time points		
	104		5234 (1799)	6104 (1891)	between groups	13.89	9.49
Keogh 2008	12	6000	6242 (4576)	6262 (3876)	“did not differ”	6.81	7.15
	52		–	–	NA	–	–
Klemsdal 2010	All	2100 deficit	–	–	NA	–	–
Krauss 2006	12	4200 deficit	–	–	NA	–	–
Krebs 2012	12	2000 deficit	7400 (3057)	6815 (1841)		9.71	8.32
	52		7258 (2098)	6784 (1792)	p = 0.012		
	104		7170 (1974)	7093 (1851)	over 104 weeks	11.24	8.71
Larsen 2011	12	6400 or 30% restriction	6449 (2652)	6029 (2652)	p = 0.22 for “group by	1.85	8.37
	52	balance	6664 (3233)	6628 (3233)	time interaction”	4.00	8.09
Lasker 2008	16	7100	6607 (1175)	5875 (1955)	p>0.10	2.45	8.77
Layman 2009	16	7100	6730 (1659)	6200 (1714)	p>0.05	3.16	6.93
	52		7118 (1793)	6800 (1917)	p>0.05	6.32	4.69
Lim 2010	12	6500	7706 (868)	7659 (1044)	–		
	24		7367 (1372)	6449 (1668)		11.10	2.77
	52		7726 (1609)	7124 (2287)			
	64		6841 (1348)	6593 (1503)		41.28	8.10
Luscombe 2003	12	6500	6358 (585)	6663 (819)	p>0.05		
	16	8200	8068 (1542)	8235 (263)	p>0.05	6.18	4.14
Parker 2002	8	6720	6665 (771)	6480 (977)	“not different”		
	12	balance	8522 (1178	7497 (1645)	“not different”	3.59	5.54
Sacks 2009	24	3150 deficit	6821 (2033)	6871 (2033)	“similar between	10.11	10.07
	104		5935 (1793)	6430 (2016)	groups”	10.04	14.24
Wycherley 2012	12	7000	7134 (771)	7189 (535)	p = 0.73	3.83	7.83
	52		7629 (1085)	7243 (739)	p = 0.09	7.64	11.55

–: not reported; CHO: carbohydrate; f: females, m: males; kJ: kilojoules; NA: not applicable; SD: standard deviation

a Arbitrary adherence score, calculated using a Mahalanobis distance equation, represents the degree of deviation from the prescribed goals for macronutrients in the two diet groups. A lower score reflects better adherence and a higher score reflects poorer adherence.

Thirteen and eight trials reported mean CHO, fat and protein intakes at 3–6 months and 1–2 years, respectively (Table 9). Calculated adherence scores were variable across the two diet groups and follow-up categories. Four trials showed similar adherence (difference in scores between groups <1) to prescribed macronutrient goals in the two diet groups after 3–6 month follow-up [16], [29]–[31]. Five trials showed better adherence in the low CHO diet groups [35]–[37], [40], [41] and four trials showed better adherence in the balanced diet group [28], [34], [38], [39]. At 1–2 years follow-up, there were greater discrepancies in the adherence scores between the two diet groups. The low CHO diet group showed better adherence to macronutrient prescriptions in three trials [16], [35], [41] and the balanced diet group showed better adherence in five trials [29], [30], [34], [37], [38] (Table 9).

### Effects of interventions

The effect estimates between the two dietary variants (high fat and high protein) did not show a qualitative difference and the heterogeneity between the groups was small or not detectable, so we pooled data across the two low CHO diet variants in the analysis.

### Trials in participants without type 2 diabetes

#### Total weight loss

At 3–6 months, the average weight loss in trials in the low CHO group ranged from 2.65 to 10.2 kg and in the isoenergetic balanced diet group from 2.65 to 9.4 kg. At 1–2 years, the range of weight loss was 2.9 to 12.3 kg with low CHO diets and 3.5 to 10.9 kg with isoenergetic balanced diets.

**Figure 3:**
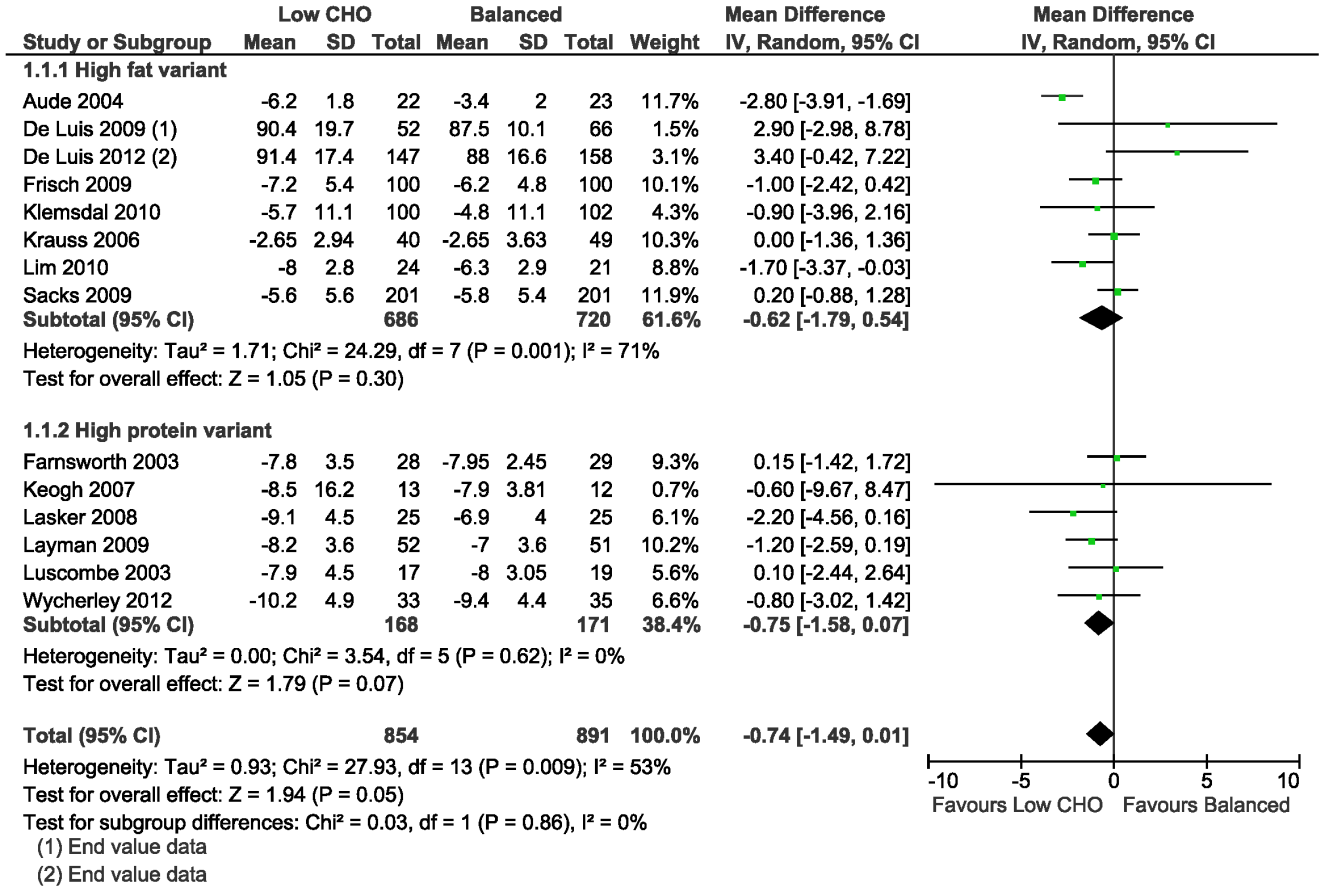
Forest plot of low carbohydrate versus balanced diets in overweight and obese adults for weight loss (kg) at 3–6 months.

**Figure 4:**
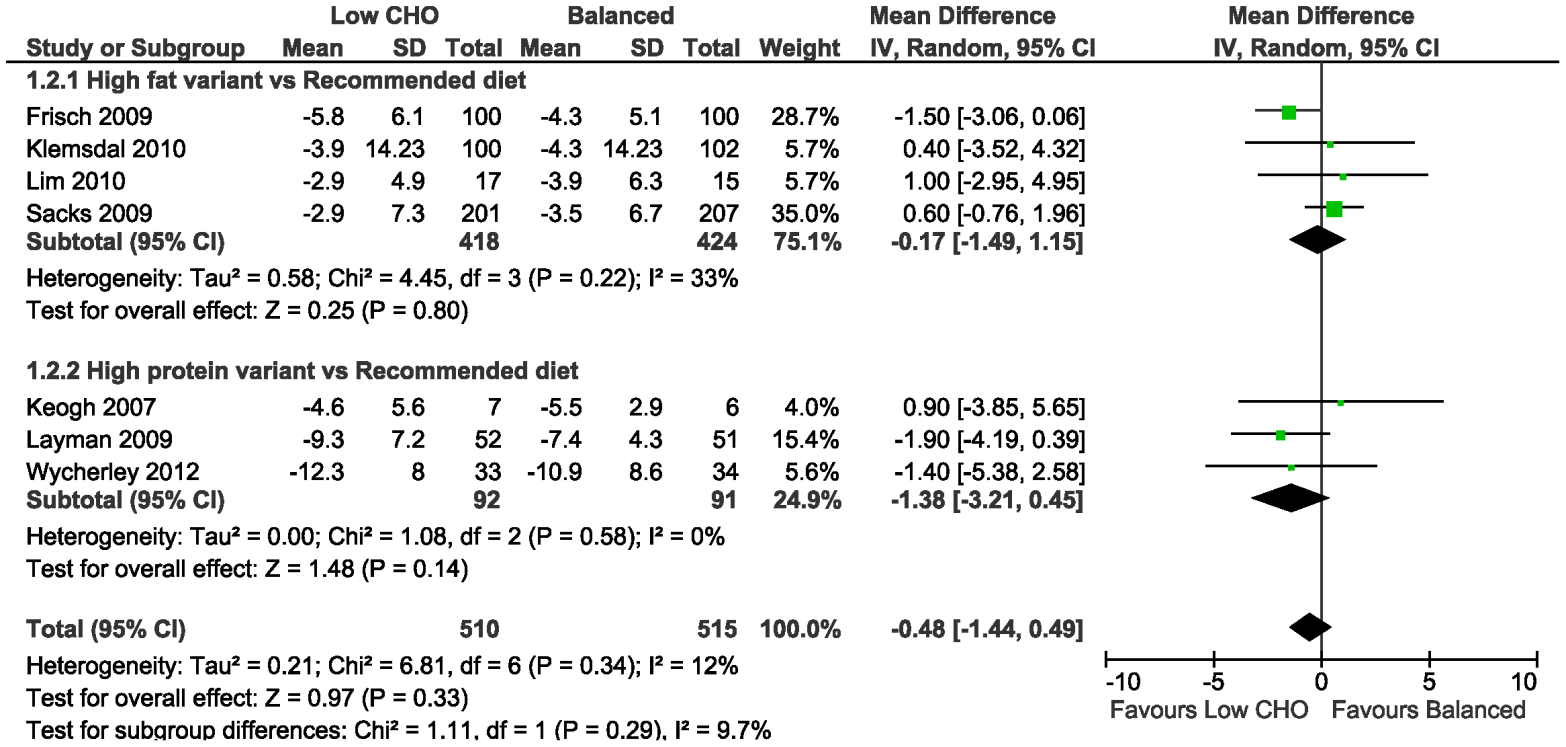
Forest plot of low carbohydrate versus balanced diets in overweight and obese adults for weight loss (kg) at 1–2 years.

The meta-analysis of the mean difference in weight loss between the low CHO and balanced diets did not demonstrate a difference at 3–6 months (−0.74 kg, 95%CI −1.49 to 0.01; 14 trials) (Table 10; Figure 3); and at 1–2 years (−0.48 kg, 95%CI −1.44 to 0.49; 7 trials) (Table 11; Figure 4). In the study [16] that concealed allocation, there was no mean difference in weight loss at 3–6 months (0.20 kg, 95%CI −0.88 to 1.28; n = 402) and at 1–2 years (0.60 kg, 95%CI −0.76 to 1.96).

**Table 10:** Summary of findings for meta-analysis of low carbohydrate diets compared with balanced diets for overweight and obese adults: 3–6 months follow-up.

**Patient or population:** overweight and obese adults without type 2 diabetes
**Settings:** primary care
**Intervention:** low carbohydrate diets (includes high fat and high protein variants)
**Comparison:** balanced diets
**Follow-up:** 3–6 months after starting diet

Outcomes	Balanced diets	Low carbohydrate diets	No. of participants (studies)	Quality of the evidence (GRADE)
	*Illustrative range of change in average values from pre-diet levels by study: range across studies* a	*The effect difference with low carbohydrate diets in randomised comparison to balanced diets (95%CI)*		
Weight loss	Lower by 2.65 to 9.4 kg	0.74 kg more weight lost	1745	⊕⊕⊕⊝
		(could be 1.49 lost to a gain of 0.01)	(14 studies)	moderate^1^^,^ 2
BMI	Lower by 1.6 to 2.4 kg/m^2^	0.25 kg/m^2^ lower BMI	673	⊕⊕⊕⊝
		(could be 0.64 lower to 0.13 higher)	(4 studies)	moderate^3^
Diastolic	Lower by 1 to 14 mmHg	0.08 mmHg lower diastolic blood pressure	1362	⊕⊕⊝⊝
blood pressure		(could be 1.53 lower to 1.36 higher)	(8 studies)	low^4^^,^ 5
Systolic	Lower by 1 to 16 mmHg	1.26 mmHg lower systolic blood pressure	1057	⊕⊕⊕⊝
blood pressure		(could be 2.67 lower to 0.15 mmHg higher)	(7 studies)	moderate^6^
LDL cholesterol	From 0.03 lower to 0.82 mmol/L higher	0.09 mmol/L higher LDL cholesterol	1603	⊕⊕⊕⊝
		(could be 0 to 0.18 mmol/L higher)	(12 studies)	moderate^7^
HDL cholesterol	From 0.1 lower to 0.08 mmol/L higher	0.03 mmol/L higher HDL cholesterol	1603	⊕⊕⊝⊝
		(could be 0.01 lower to 0.08 mmol/L higher)	(12 studies)	low^8^^,^ 9
Total cholesterol	Lower by 0.07 to 0.88 mmol/L	0.08 mmol/L higher total cholesterol	1603	⊕⊕⊕⊝
		(could be 0.02 lower to 0.17 mmol/L higher)	(12 studies)	moderate^7^
Triglycerides	From 0.49 lower to 0.01 mmol/L higher	0.05 mmol/L lower triglycerides	1603	⊕⊕⊝⊝
		(could be 0.14 lower to 0.04 mmol/L higher)	(12 studies)	low^10^^,^ 11

CI: Confidence interval ;

a Note this is the univariate average change observed between follow-up and baseline in the control group.

GRADE Working Group grades of evidence.

High quality: Further research is very unlikely to change our confidence in the estimate of effect.

Moderate quality: Further research is likely to have an important impact on our confidence in the estimate of effect and may change the estimate.

Low quality: Further research is very likely to have an important impact on our confidence in the estimate of effect and is likely to change the estimate.

Very low quality: We are very uncertain about the estimate.

1 Downgraded by 1 for risk of bias: 8 of 14 studies did not report adequate sequence generation and 13 studies did not report adequate allocation concealment. 4 studies had high total attrition (>20%) and 2 other studies had differential attrition.

2 Not downgraded for inconsistency: no qualitative heterogeneity; some quantitative heterogeneity, to be expected.

3 Downgraded by 1 for risk of bias: 1 study did not report adequate sequence generation, none of the studies reported on allocation concealment and 1 study had high total attrition (>20%).

4 Downgraded by 1 for risk of bias: 5 of 8 studies did not report adequate sequence generation and 7 studies did not report adequate allocation concealment. 2 studies had high total attrition (>20%).

5 Downgraded by 1 for inconsistency: Mean differences were on opposite sides of the line of no difference (I^2^ 51%).

6 Downgraded by 1 for risk of bias: 5 of 8 studies did not report adequate sequence generation and 7 studies did not report adequate allocation concealment. 2 studies had high total attrition (>20%).

7 Downgraded by 1 for risk of bias: 5 of 12 studies did not report adequate sequence generation and 11 studies did not report adequate allocation concealment. 3 studies had high total attrition (>20%) and 2 other studies had differential attrition.

8 Downgraded by 1 for risk of bias: 6 of 12 studies did not report adequate sequence generation and 11 studies did not report adequate allocation concealment. 3 studies had high total attrition (>20%) and 2 studies had differential attrition.

9 Downgraded by 1 for inconsistency: Mean differences were on opposite sides of the line of no difference (I^2^ 63%).

10 Downgraded by 1 for risk of bias: 6 of 12 studies did not report adequate sequence generation and 11 studies did not report adequate allocation concealment. 3 studies had had total attrition (>20%) and 2 studies had differential attrition.

11 Downgraded by 1 for inconsistency: Mean differences were on opposite sides of the line of no difference (I^2^ 72%).

**Table 11:** Summary of findings for low carbohydrate diets compared with balanced diets for overweight and obese adults at 1–2 years follow-up.

**Patient or population:** overweight and obese adults without type 2 diabetes
**Settings:** primary care
**Intervention:** low carbohydrate diets (includes high fat and high protein variants)
**Comparison:** balanced diets
**Follow-up:** 1–2 years after starting diet

Outcomes	Balanced diets	Low carbohydrate diets	No. of participants (studies)	Quality of the evidence (GRADE)
	*Illustrative range of change in average values from pre-diet levels by study: range across studies* a	*The effect difference with low carbohydrate diets in randomised comparison to balanced diets (95%CI)*		
Weight loss	Lower by 3.5 to 10.9 kg	0.48 kilograms more weight lost	1025	⊕⊕⊕⊝
		(could 1.44 lost to a gain of 0.49 kg)	(7 studies)	moderate^1^
BMI	Lower by 1.5 kg/m^2^	0.40 kg/m^2^ lower BMI (high fat variant only)	200	⊕⊕⊝⊝
(at 1 year)		(could be 0.94 lower to 0.14 higher)	(1 study)	low^2^^,^ 3
Diastolic	Lower by 1 to 11 mmHg	0.03 mmHg lower diastolic blood pressure	914	⊕⊕⊕⊝
blood pressure		(could be 1.68 lower to 1.62 mmHg higher)	(6 studies)	moderate^4^
Systolic	From 10 lower to 8 mmHg higher	2 mmHg lower systolic blood pressure	914	⊕⊕⊕⊝
blood pressure		(could be 5 lower to 1 mmHg higher)	(6 studies)	moderate^4^
LDL cholesterol	From 0.79 lower to 0.06 mmol/L higher	0.07 mmol/L higher LDL cholesterol	915	⊕⊕⊕⊝
		(could be 0.01 lower to 0.16 mmol/L higher)	(6 studies)	moderate^4^
HDL cholesterol	From 0.03 lower to 0.15 mmol/L higher	0.04 mmol/L higher HDL cholesterol	986	⊕⊕⊕⊝
		(could 0.01 to 0.08 mmol/L higher)	(7 studies)	moderate^1^
Total cholesterol	From 0.76 lower 0.13 mmol/L higher	0.06 mmol/L higher total cholesterol	915	⊕⊕⊕⊝
		(could be 0.03 lower to 0.16 mmol/L higher)	(6 studies)	moderate^4^
Triglycerides	From 0.44 lower to 0.06 mmol/L higher	0.06 mmol/L lower triglycerides	915	⊕⊕⊕⊝
		(could be 0.14 lower to 0.03 mmol/L higher)	(6 studies)	moderate^4^

CI: Confidence interval;

a Note this is the univariate average change observed between follow-up and baseline in the control group.

GRADE Working Group grades of evidence.

High quality: Further research is very unlikely to change our confidence in the estimate of effect.

Moderate quality: Further research is likely to have an important impact on our confidence in the estimate of effect and may change the estimate.

Low quality: Further research is very likely to have an important impact on our confidence in the estimate of effect and is likely to change the estimate.

Very low quality: We are very uncertain about the estimate.

1 Downgraded by 1 for risk of bias: 5 of 7 studies did not report adequate sequence generation and only 1 reported adequate allocation concealment. 5 studies were judged to have a high or unclear risk of attrition bias.

2 Downgraded by 1 for risk of bias: the study did not report adequate allocation concealment and reasons for attrition differed between groups.

3 Downgraded by 1 for imprecision: difference in mean BMI change ranges from a reduction of −0.94 to an increase of 0.14 kg/m^2^ (approximately equivalent to 2 to 4 kilograms).

4 Downgraded by 1 for risk of bias: 4 of 6 studies did not report adequate sequence generation and 5 studies did not report adequate allocation concealment. 2 studies had high total attrition (>20%), 1 of which also had differential attrition.

A few studies reported change in BMI. As with weight, average BMI was lower after dieting in both diet groups, but with no difference detected at either 3–6 months across the 4 trials reporting this (Table 10; Figure S2A in Supporting Information S2), or in the one trial reporting this at 1–2 years (Table 11; Figure S2B in Supporting Information S2).

#### Blood pressure

At 3–6 months, the average DBP compared to baseline in each study was reduced in both the low CHO group (range: −10 to −1 mmHg) and in those on balanced diets (range: −14 to −1 mmHg). At 1–2 years, the average drop within studies compared to baseline ranged from 9 mmHg lower to no change in DBP with low CHO and a reduction across studies with balanced diets of 11 to 1 mmHg.

The meta-analyses of the mean difference in DBP change did not demonstrate a difference between the low CHO and balanced diets at 3–6 months (95%CI −1.53 to 1.36; 8 trials) (Table 10; Figure S2C in Supporting Information S2) and at 1–2 years (95%CI −1.68 to 1.62; 6 trials) (Table 11; Figure S2D in Supporting Information S2). (In one of the trials [26], the mean SBP value after three months in the low CHO group was reported as 103.1±13.7 mmHg (corresponding reported mean baseline value of 138.6±16.8 mmHg). We suspected this very low SBP value to be a typographical error, but did not receive a response after contacting the authors and therefore excluded this data from the meta-analysis.)

At 3–6 months, the average SBP in each study compared to baseline showed a drop in both the low CHO (range: −15 to −2 mmHg) and balanced diet groups (range: −16 to −1 mmHg) in all trials. At 1–2 years, average SBP decreased with low CHO (range: −10.6 to −0.9 mmHg) and either decreased or increased with balanced diets (range: −10 to 8 mmHg). The increase was observed in a small trial (n = 25) with 48% attrition when the trial ended after one year [31].

The meta-analysis of the mean difference in SBP change showed no difference after 3–6 months (−1.26 mmHg, 95%CI −2.67 to 0.15; 7 trials) (Table 10; Figure S2E in Supporting Information S2) and after 1–2 years (−2.00 mmHg, 95%CI −5.00 to 1.00; 6 trials) (Table 11; Figure S2F in Supporting Information S2).

#### Blood lipids

At 3–6 months, compared to baseline, average LDL and total cholesterol were inconsistent across trials with low CHO diets (range LDL: −0.62 to 0.3 mmol/L; total cholesterol: −0.71 to 0.1 mmol/L), while these values decreased with balanced diets in each of the 12 trials that reported these values (range LDL: −0.82 to −0.03 mmol/L; total cholesterol: −0.88 to −0.07 mmol/L). Average changes in HDL and TG from baseline varied with low CHO (range HDL: −0.07 to 0.1 mmol/L; TG: −0.64 to 0.01 mmol/L) and balanced diets (range HDL: −0.1 to 0.08 mmol/L; TG: −0.49 to 0.01 mmol/L). At 1–2 years, average lipid marker changes from baseline were inconsistent in both diet groups across trials, with variations in ranges of change that were similar to those reported at 3–6 months.

The meta-analyses of the mean differences in blood lipids between the low CHO and balanced diets were small in both follow-up categories, with narrow confidence intervals suggesting little or no difference in effect between the two diets (Tables 10 and 11; Figures S2G to S2N in Supporting Information S2).

#### Fasting blood glucose

From baseline to 3–6 months, average FBG decreased with low CHO (range −0.47 to −0.06 mmol/L) and balanced diets (range −0.52 to −0.1 mmol/L), and at 1–2 years average changes were variable with low CHO (range: −0.71 to 0.17 mmol/L) and balanced diets (range: of −0.4 to 0.06 mmol/L). The meta-analysis showed no difference between low CHO and balanced diets in FBG change at either 3–6 months (0.05 mmol/l, 95%CI −0.05 to 0.15; 10 trials; Figure S2O in Supporting Information S2) or 1–2 years (0.0 mmol/L, 95%CI −0.16 to 0.16; 6 trials; Figure S2P in Supporting Information S2).

### Trials in participants with type 2 diabetes

#### Total weight loss

**Figure 5:**
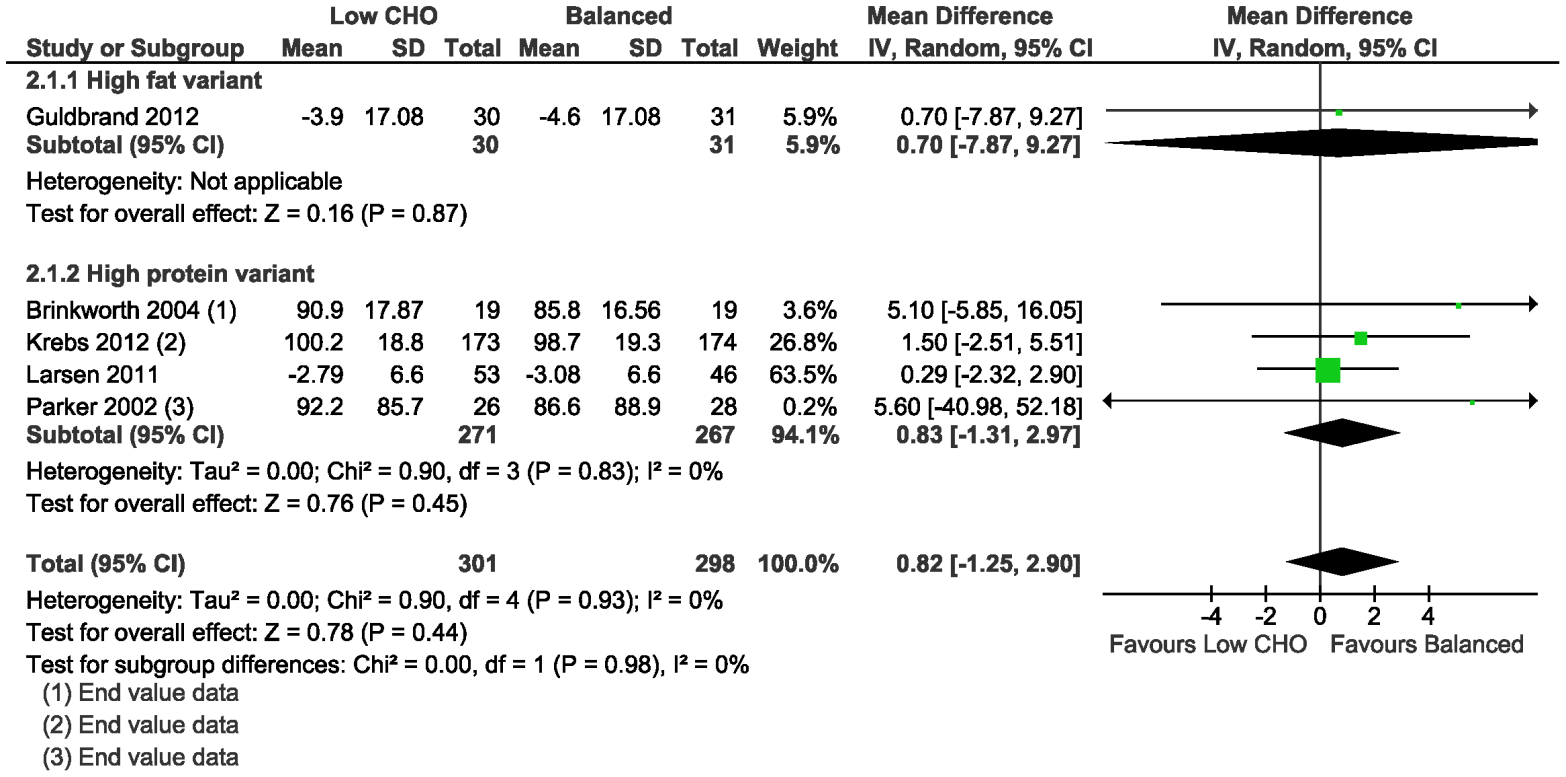
Forest plot of low carbohydrate versus balanced diets in overweight and obese adults with type 2 diabetes for weight loss (kg) at 3–6 months.

**Figure 6:**
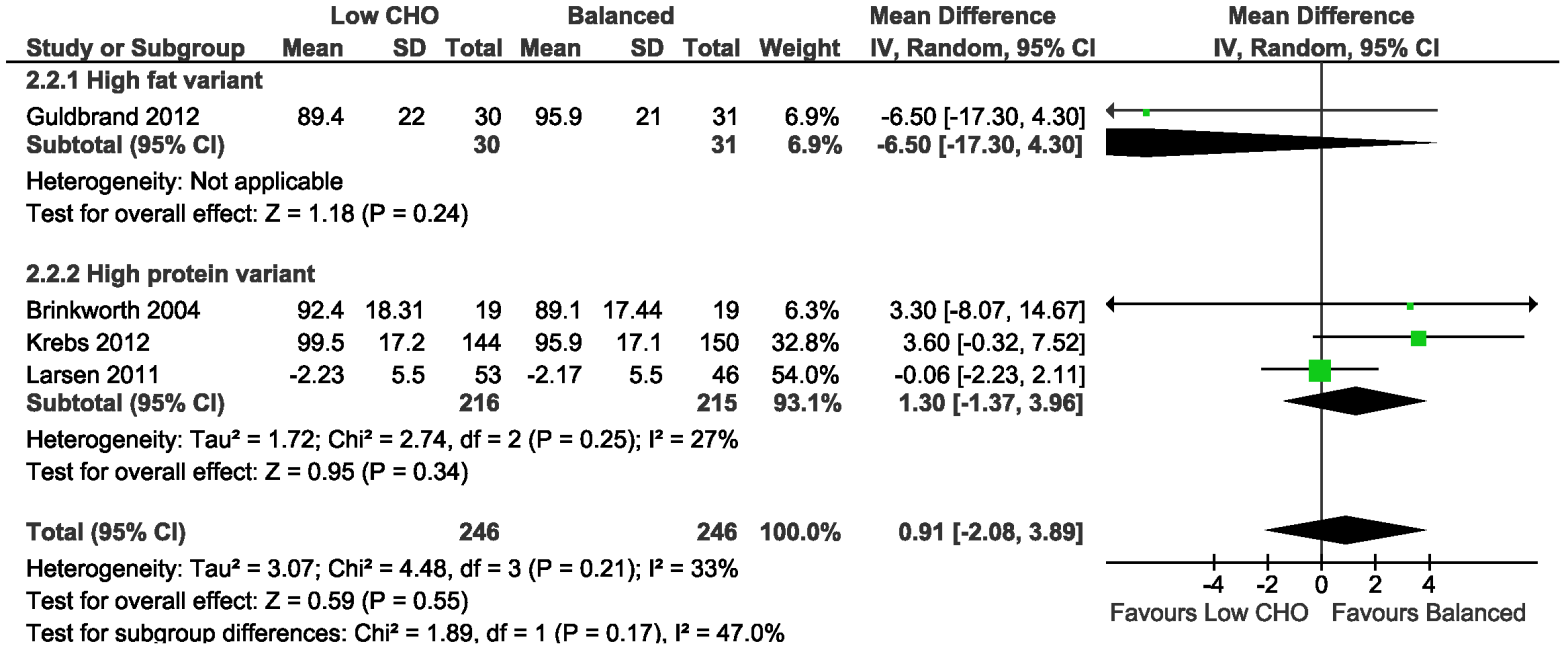
Forest plot of low carbohydrate versus balanced diets in overweight and obese adults with type 2 diabetes for weight loss (kg) at 1–2 years.

Average weight loss was evident at 3–6 months with low CHO (range: 2.79 to 5.5 kg) and isoenergetic balanced diets (range: 3.08 to 5.4 kg), and similarly with both diets at 1–2 years (range low CHO diets: 2 to 3.9 kg; range balanced diets: 2.1 to 6 kg) in all trials. The meta-analysis of the mean difference in weight loss between the low CHO and balanced diets did not demonstrate a difference at 3–6 months (0.82 kg, 95%CI −1.25 to 2.90; 5 trials) (Table 12; Figure 5); and at 1–2 years (0.91 kg, 95%CI −2.08 to 3.89; 4 trials) (Table 13; Figure 6).

**Table 12:** Summary of findings for low carbohydrate diets compared with balanced diets for overweight and obese adults with type 2 diabetes mellitus at 3–6 months follow-up.

**Patient or population:** overweight or obese adults with type 2 diabetes
**Settings:** primary care
**Intervention:** low carbohydrate diets (includes high fat and high protein variants)
**Comparison:** balanced diets
**Follow-up:** 3–6 months after starting diet

Outcomes	Balanced diets	Low carbohydrate diets	No. of participants (studies)	Quality of the evidence (GRADE)
	*Illustrative range of change in average values from pre-diet levels by study: range across studies* a	*The effect difference with low carbohydrate diets in randomised comparison to balanced diets (95%CI)*		
Weight loss	Lower by 3.08 to 5.4 kg	0.82 kg less weight lost	599	⊕⊕⊝⊝
		(could be 1.25 lost to a gain of 2.9 kg)	(5 studies)	low^1^^,^ 2
HbA1c	Lower by 0.3 to 0.51%	0.19% higher HbA1c	599	⊕⊕⊕⊝
		(could be 0 to 0.39% higher)	(5 studies)	moderate^3^
Diastolic	From 3 lower to 1.63 mmHg higher	0.77 mmHg higher diastolic blood pressure	545	⊕⊕⊕⊝
blood pressure		(could be 1.77 lower to 3.3 mmHg higher)	(4 studies)	moderate^4^
Systolic	Lower by 0.06 to 8 mmHg	0.61 mmHg higher systolic blood pressure	545	⊕⊕⊝⊝
blood pressure		(could be 3.14 lower to 4.36 mmHg higher)	(4 studies)	low^4^^,^ 5
LDL cholesterol	From 0.11 lower to 0.09 mmol/L higher	0.06 mmol/L higher LDL cholesterol	599	⊕⊕⊕⊝
		(could be 0.11 lower to 0.23 mmHg higher)	(5 studies)	moderate^3^
HDL cholesterol	From 0.01 lower to 0.03 mmol/L higher	0.01 lower HDL cholesterol	599	⊕⊕⊕⊝
		(could be 0.05 lower to 0.04 mmol/L higher)	(5 studies)	moderate^3^
Total cholesterol	Lower by 0.01 to 0.31 mmol/L	0.04 mmol/L higher total cholesterol	599	⊕⊕⊕⊝
		(could be 0.21 lower to 0.3 mmol/L higher)	(5 studies)	moderate^3^
Triglycerides	From 0 to 0.45 mmol/L lower	0.20 mmol/L lower triglycerides	252	⊕⊕⊝⊝
		(could be 0.45 lower to 0.05 mmol/L higher)	(4 studies)	low^6^^,^ 7

**CI:** Confidence interval;

a Note this is the univariate average change observed between follow-up and baseline in the control group.

GRADE Working Group grades of evidence.

High quality: Further research is very unlikely to change our confidence in the estimate of effect.

Moderate quality: Further research is likely to have an important impact on our confidence in the estimate of effect and may change the estimate.

Low quality: Further research is very likely to have an important impact on our confidence in the estimate of effect and is likely to change the estimate.

Very low quality: We are very uncertain about the estimate.

1 Downgraded by 1 for risk of bias: 1 of 5 studies did not report adequate sequence generation and 3 of 5 studies did not report adequate allocation concealment. 1 study had high total attrition (>20%) and 2 studies had differential attrition.

2 Downgraded by 1 for imprecision: difference in mean weight loss ranges from a loss of 1.25 to a gain of 2.9 kilograms.

3 Downgraded by 1 for risk of bias: 1 out of 5 studies did not report adequate sequence generation and 3 out of 5 studies did not report allocation concealment. 1 study had high total attrition and 2 studies had differential attrition.

4 Downgraded by 1 for risk of bias: 2 of 4 studies did not report adequate allocation concealment. 1 study had high total attrition (>20%) and 2 studies had differential attrition.

5 Downgraded by 1 for imprecision: difference in mean systolic blood pressure ranges from a reduction of 3.14 to an increase of 4.36 mmHg.

6 Downgraded for risk of bias: 1 of 4 studies did not report adequate sequence generation and 2 studies did not report adequate allocation concealment. 2 studies had differential attrition.

7 Downgraded by 1 for imprecision: confidence interval range is 0.5 mmol/L.

**Table 13:** Summary of findings for low carbohydrate diets compared with balanced diets for overweight and obese adults with type 2 diabetes mellitus at 1–2 years follow-up.

**Patient or population:** overweight or obese adults with type 2 diabetes
**Settings:** primary care
**Intervention:** low carbohydrate diets (high fat and high protein variants combined)
**Comparison:** balanced diets
**Follow-up:** 1–2 years after starting diet

Outcomes	Balanced diets	Low carbohydrate diets	No. of participants (studies)	Quality of the evidence (GRADE)
	*Illustrative range of change in average values from pre-diet levels by study: range across studies* a	*The effect difference with low carbohydrate diets in randomised comparison to balanced diets (95%CI)*		
Weight loss	Lower by 2.1 to 6 kg	0.91 kg less weight lost	492	⊕⊕⊝⊝
		(could be 2.08 lost to a gain of 3.89)	(4 studies)	low^1^^,^ 2
HbA1c	From 0.28% lower to 0.4% higher	0.01% higher HbA1c	492	⊕⊕⊕⊝
		(could be 0.28 lower to 0.3 higher)	(4 studies)	moderate^3^
Diastolic	From 6 lower to 2.5 mmHg higher	0.09 mmHg higher diastolic blood pressure	492	⊕⊕⊕⊝
blood pressure		(could be 1.95 lower to 2.13 higher)	(4 studies)	moderate^1^
Systolic	From 11 lower to 3.7 mmHg higher	0.31 mmHg higher systolic blood pressure	492	⊕⊕⊕⊝
blood pressure		(could be 3.1 lower to 3.72 higher)	(4 studies)	moderate^1^
LDL cholesterol	From 0.3 lower to 0.04 mmol/L higher	0.10 mmol/L higher LDL cholesterol	492	⊕⊕⊕⊝
		(could be 0.06 lower to 0.27 higher)	(4 studies)	moderate^3^
HDL cholesterol	Higher by 0.02 to 0.19 mmol/L	No difference in HDL cholesterol	492	⊕⊕⊕⊝
		(could be 0.09 lower to 0.08 higher)	(4 studies)	moderate^3^
Total cholesterol	From 0.3 lower to 0.35 mmol/L higher	0.10 mmol/L higher total cholesterol	492	⊕⊕⊕⊝
		(could be 0.12 lower to 0.31 mmol/L higher)	(4 studies)	moderate^3^
Triglycerides	Lower by 0.1 to 0.3 mmol/L	0.08 mmol/L lower triglycerides	198	⊕⊕⊝⊝
		(could be 0.49 lower to 0.26 higher)	(3 studies)	low^4^^,^ 5

CI: Confidence interval;

a Note this is the univariate average change observed between follow-up and baseline in the control group.

GRADE Working Group grades of evidence.

High quality: Further research is very unlikely to change our confidence in the estimate of effect.

Moderate quality: Further research is likely to have an important impact on our confidence in the estimate of effect and may change the estimate.

Low quality: Further research is very likely to have an important impact on our confidence in the estimate of effect and is likely to change the estimate.

Very low quality: We are very uncertain about the estimate.

1 Downgraded by 1 for risk of bias: 2 of 4 studies did not report adequate allocation concealment. 1 study had high total attrition (>20%) and 2 studies had differential attrition.

2 Downgraded by 1 for imprecision: The 95% confidence interval includes both a loss of 2.08 kg and a gain of 3.89 kg.

3 Downgraded by 1 for risk of bias: 2 of 4 studies did not report adequate allocation concealment, 2 studies had high total attrition (>20%), 2 studies had differential attrition.

4 Downgraded by 1 for risk of bias: 1 of 3 studies did not report adequate allocation concealment. 2 studies had high total attrition (>20%), 2 studies had differential attrition.

5 Downgraded by 1 for imprecision: confidence interval range is about 0.7 mmol/L.

A single trial found no difference in BMI change between the low CHO (high fat variant) and balanced diets at 3–6 months (Figures S3A and S3B in Supporting Information S3).

#### Markers of glycaemic control

At 3–6 months, compared to baseline, changes in average HbA1c varied across studies with low CHO diets (range: −0.54 to 0%), and decreased in each study with balanced diets (range: −0.51 to −0.3%). At 1–2 years, average HbA1c changes from baseline were inconsistent in both diet groups across trials (range low CHO: −0.23 to 0.1%; balanced: −0.28 to 0.4%).

The meta-analyses of the mean difference in HbA1c change did not demonstrate a difference between the low CHO and balanced diets at 3–6 months (0.19%, 95%CI −0.0 to 0.39; 5 trials) (Table 12; Figure S3C in Supporting Information S3) and at 1–2 years (0.01%, 95%CI −0.28 to 0.30, 4 trials) (Table 13; Figure S3D in Supporting Information S3).

Similarly, no mean difference in FBG change between low CHO and balanced diets was detected by meta-analysis of 2 studies at 3–6 months (Figure S3E in Supporting Information S3). One trial reported no difference in FBG change after 15 months (Figure S3F in Supporting Information S3).

#### Blood pressure

Average changes in DBP from baseline varied at 3–6 months with low CHO (range: −4 to 2.24 mmHg) and balanced diets (range: −3 to 1.63 mmHg) and also at 1–2 years (range low CHO: −5 to 0.21 mmHg; balanced: −6 to 2.5 mmHg).

The meta-analyses of the mean difference in DBP change did not demonstrate a difference between the low CHO and balanced diets at 3–6 months (95%CI −1.77 to 3.30; 4 trials) (Table 12; Figure S3G in Supporting Information S3) and at1–2 years (95%CI −1.95 to 2.13, 4 trials) (Table 13; Figure S3H in Supporting Information S3).

The average SBP in each study compared to baseline showed a drop in both the low CHO (range: −9 to −1 mmHg) and balanced diets (range: −8 to −0.06 mmHg) at 3–6 months, with varied changes at 1–2 years (range low CHO: −9 to 2.2 mmHg; balanced: −11 to 3.7 mmHg).

The meta-analysis of the mean difference in SBP change showed no difference after 3–6 months (95%CI −3.14 to 4.36; 4 trials) (Table 12; Figure S3I in Supporting Information S3) and after 1–2 years (95%CI −3.10 to 3.72; 4 trials) (Table 13; Figure S3J in Supporting Information S3).

#### Blood lipids

At 3–6 months, blood lipids (LDL, HDL, total cholesterol, TG) showed variable changes from baseline in both low CHO and balanced diets. Overall, changes from baseline were inconsistent between the diet groups and for both follow-up categories. The changes on meta-analysis were small suggesting little or no difference in effect between the two diets (Table 12 and 13; Figures S3K to S3R in Supporting Information S3).

## Discussion

This review, including 19 RCTs with 3209 participants showed there is probably little or no difference in changes in weight and cardiovascular and diabetes risk factors with low CHO weight loss diets compared to isoenergetic balanced weight loss diets. This was in both overweight and obese adults without diabetes and those with diabetes, with follow-up for up to two years. When reported, energy intake was similar in the diet groups being compared, but participants did not adhere fully to the prescribed macronutrient goals for both diets in most trials.

### Overweight and obese adults without type 2 diabetes

#### Weight loss

Participants lost weight in both groups, with similar before and after average loss after 3–6 months, and 1–2 years of follow-up. There was little or no difference in weight loss and change in BMI between the low CHO and balanced weight loss diets in the two follow-up periods. The similar reported mean energy intakes in the low CHO and balanced diet groups and the corresponding similar average weight loss in the diet groups supports the fundamental physiologic principle of energy balance, namely that a sustained energy deficit results in weight loss regardless of macronutrient composition of the diet [43].

Norms for defining “stable weight” are gaining less than or equal to 2 kg and losing less than 2 kg [44] indicating that both low CHO and balanced weight loss diets (or energy-restricted diets) result in meaningful weight loss. Clearly, the goal of any healthy weight loss strategy should be to achieve weight loss and to subsequently maintain this over the long-term. The 2013 AHA/ACC/TOS Guideline for the Management of Overweight and Obesity in Adults state that strategies for weight maintenance after successful loss differ from the strategies for achieving weight loss and make recommendations in this regard [44].

#### Impacts on markers of cardiovascular risk

Weight loss improves markers of cardiovascular risk [45]–[47]. According to the 2013 AHA/ACC/TOS Guideline [44], based largely on systematic reviews, clinically meaningful changes in CVD risk indicators are associated with a loss of at least 2.5 kg, or 2% of body weight, achieved with lifestyle interventions over one to four years. This document states that at a 5% weight loss, a weighted mean reduction in DBP of about 2 mmHg and in SBP of about 3 mmHg is observed [44]. Correspondingly, the weight loss in both diet groups in our review was accompanied by reductions in average DBP and SBP in all trials. In line with the weight loss findings, there is probably little or no difference in SBP changes after 3–6 months and there may be little or no difference in DBP changes between the low CHO and balanced diet groups. After 1–2 years, there is probably little or no difference in changes in DBP and SBP between the diet groups. These judgements are based on both the meta-analyses and the quality of the evidence for these outcomes per length of follow-up category.

When considering blood lipid changes, a weight loss of 5 kg to 8 kg is reported to result in LDL cholesterol reduction of approximately 0.13 mmol/L and an increase in HDL cholesterol of between 0.05 to 0.08 mmol/L [44]. In overweight and obese adults with and without CVD risk who lose 3 kg on a lifestyle intervention, a weighted reduction in serum TG of approximately 0.17 mmol/L is observed [44]. In the trials in our review, effects on blood lipids and FBG with low CHO and balanced diets were variable, with greater and lesser average changes in LDL, HDL and TG than the observations described above. When comparing low CHO and isoenergetic balanced diets, the pooled mean differences across the trials and quality of evidence indicate that there is probably little or no difference in changes in LDL, HDL and total cholesterol and there may be little or no difference in TG change at 3–6 months. Similarly, after 1–2 years, there is probably little or no difference in serum LDL and total cholesterol and TG between the diet groups. Meta-analysis of HDL cholesterol difference was 0.04 mmol/L higher with low CHO diets compared to balanced diets after 1–2 years, but the difference was not clinically meaningful, and no difference was detected for LDL.

The primary reason for the moderate grade of evidence in most outcomes at 3–6 months and 1–2 years is the risk of selection, performance and attrition bias in most included trials. For serum triglycerides, inconsistency (as discussed above) in effects resulted in further downgrading to low quality indicative of less confidence in the findings. Similarly, for DBP at 3–6 months, inconsistency in the mean differences across the different trials resulted in further downgrading to low quality evidence. This inconsistency could not be explained by the different variants of the low CHO diet. Most of the inconsistency can be ascribed to two trials [32], [41] with similar weights in the meta-analysis (19.5% and 15.5%, respectively) that produced significant opposite mean differences for DBP. Klemsdal and colleagues [32] found that the low CHO diet reduced DBP more than the balanced diet (−3.40 mmHg, 95%CI −6.02 to −0.78). They reported that this observation should be interpreted with some caution, since blood pressure was a secondary endpoint in the study and the effect on SBP did not differ between the two groups. This effect was no longer significant at one year. In contrast, Wycherley and colleagues [41] reported a greater reduction in DBP with the balanced diet compared to the low CHO diet (4.00 mmHg, 95%CI 0.58 to 7.42). Similarly, this difference in effect was not found for SBP and disappeared at one year. The heterogeneity may also be attributable to differences in dietary adherence, as well as mean baseline DBP in one trial [27] that could be judged as being imbalanced (85.8 and 80.7 mmHg in low CHO and balanced diet groups, respectively). Although not reported, it could be argued that differences in the sodium and potassium content of the intervention diets may explain some of the variable effects on DBP.

### Overweight and obese adults with type 2 diabetes mellitus

#### Weight loss

Both low CHO diets and balanced weight loss diets showed similar weight loss on average after 3–6 months and after 1–2 years. Meta-analysis and quality of evidence indicate that in overweight and obese adults with type 2 diabetes there may be little or no difference in weight loss after 3–6 months and 1–2 years. The earlier discussion of the long-term effects of dieting on weight loss is also applicable in this population.

#### Impacts on glycaemic control and cardiovascular risk

Weight loss is associated with improvements in glycaemia in overweight and obese adults with type 2 diabetes. According to the 2013 AHA/ACC/TOS Guideline, 2% to 5% weight loss achieved with one to four years of lifestyle intervention results in modest reductions in FBG and lowering of HbA1c by 0.2% to 0.3% [44]. Along with weight loss in both diet groups in our included trials, both low CHO and balanced diet groups showed similar reductions in average HbA1c in most trials after 3–6 months. At 1–2 years average HbA1c change was more variable. Comparing these changes by combining data across trials indicated that there is probably little or no difference in changes in HbA1c between the two diets at 3–6 months and 1–2 years. The meta-analysis at 3–6 months of two small trials [25], [40] showed similar findings for FBG concentrations. Only one of these trials went on to report FBG at 15 months and had the same finding [25].

Effects on DBP with low CHO and balanced diets were variable in most trials, showing both reductions and increases. Both the low CHO and balanced weight loss diets demonstrated reductions in average SBP in all trials after 3–6 months, but effects were variable with both diets after 1–2 years. Based on both the meta-analyses and the quality of the evidence, there is probably little or no difference in DBP change between the two diets and there may be little or no difference in SBP change after 3–6 months. After 1–2 years, there is probably little or no difference in changes in both DBP and SBP.

Effects on blood lipids with low CHO and balanced diets were variable between included trials, as was seen in the non-diabetic population. Considering the meta-analyses and the quality of the evidence, there is probably little or no difference in changes in LDL, HDL and total cholesterol after 3–6 months and 1–2 years when comparing the two diets. There may be little or no difference in changes in TG concentrations after 3–6 months and 1–2 years.

As in the non-diabetic overweight and obese population, the presence of risk of selection, performance and attrition bias in most included trials were the primary reasons for the moderate grade of evidence in most outcomes in the diabetic population. For weight loss at 3–6 months and 1–2 years follow-up, imprecision of the effect estimates resulted in further downgrading to low quality evidence. Similarly, the evidence for triglycerides for both follow-up categories and for SBP at 3–6 months was downgraded due to imprecision of the effect estimates. These imprecise estimates possibly relate to the smaller samples in the diabetes population.

### Adherence

Assessment of adherence to energy prescriptions across the 19 trials was problematic due to the different methods used to express prescriptions and the lack of reported energy intake data in some trials. The dietary intake methodology used also varied between the included trials, with trials using food records/diaries, single or multiple 24 hour recalls, food frequency questionnaires or combinations of these methods.

From the calculated adherence scores it was clear that strict adherence to prescribed macronutrient goals failed with both diets in most trials and generally declined with longer follow-up. This diminished adherence after the first few months has been well documented in weight loss trials [48]–[51] and is more likely in weight loss diets involving extreme dietary changes such as drastic restrictions of entire food groups. This is supported by the fact that trials of low CHO diets have reported a very low incidence of urinary ketosis after six months [49]–[51], which suggests that most overweight participants in weight loss trials struggle to sustain a low intake of CHO. It could thus be argued that overweight participants following reduced energy weight loss diets in trials tend to revert to their usual macronutrient intakes over time, but may nonetheless, be able to lose weight if they are able to maintain the energy deficit. The novelty factor attached to a particular diet, media attention, and the opinion of the researchers involved could possibly affect the adherence of participants to any type of diet. It is clear from this and other research [52] that one of the pertinent issues in the treatment of overweight and obesity relates to the improvement of behavioural adherence to reduced dietary energy intake. It should be noted that the adherence score is based on calculations using mean reported intakes of macronutrients (% of total energy) and thus does not consider the variation around the mean.

### Overall completeness and applicability of evidence

The findings of our review need to be interpreted in light of the presence of risk of bias or lack of power or both in many of the included trials, the possibility that adherence to dietary macronutrient goals were not optimal and that there was inter-trial variation in quantity (and type) of fat consumed. The interpretation of many weight loss trials is limited by a lack of blinded ascertainment of the outcome, small samples, large loss to follow-up, potentially limited generalisability and a lack of data on adherence to assigned diets [53]. These limitations all apply to the evidence assessed in our systematic review. Strengths of our review include the clear definitions used in relation to the energy content and macronutrient composition of treatment and control diets, as well as the restriction of included studies to those testing diets only thereby reducing the risk of confounding by co-interventions. By considering only isoenergetic comparisons we also avoided the problem of the effect of energy imbalance between the comparison groups being confounded with any potential effect of macronutrient manipulation on the outcomes being investigated. Furthermore, we only included studies with follow-up of 12 weeks or more to allow for sufficient time to detect weight and CVD risk factor changes and assessed outcomes at defined lengths of follow-up. These methods differentiate our systematic review from previous reviews on this topic.

Our results show that the weight loss in overweight and obese subjects with or without diabetes on isoenergetic low CHO or balanced weight loss diets was similar at 3–6 months and at 1–2 years. Thus, the weight loss is the result of a reduction in total dietary energy intake rather than manipulation of macronutrient contribution. It follows that when considering dietary strategies for weight loss, less emphasis should be placed on an ‘ideal’ macronutrient composition and more emphasis on reduction in total energy intake, as well as improvement of behavioural adherence to reduced energy intake. This will go a long way to ensure that weight loss is achieved and maintained to gain health benefits. Guidance on macronutrient composition to meet nutritional requirements and prevent disease [12]–[15] remains integral to healthy sustainable weight management.

The small size and short duration of weight loss trials often account for their lack of definitive evidence of the effectiveness of dietary interventions on CVD risk. By contrast sound observational data, population-level interventions and “natural experiments” in whole populations have demonstrated a reduction in population risk with adoption of recommended, balanced dietary strategies to lower cardiovascular risk. For example, over the past three decades, levels of population cardiovascular risk factors have declined in Finland, with the greatest change being dietary behaviour (reduction in total and saturated fat and increased vegetables and fruit intake). These declines explain most of the observed decline in CHD mortality in the Finnish middle-aged population over this period [54]. Mortality due to coronary heart disease was reduced in Poland over a ten year period by partly replacing dietary saturated fats with polyunsaturated fats while maintaining a low intake of trans fatty acids [55]. A large prospective cohort study in 30 to 49 year old Swedish women (n = 43396; average follow-up 15.7 years) reported significantly increased incidence of cardiovascular disease overall (n = 1270) with a one tenth decrease in carbohydrate intake or increase in protein intake, or a two unit increase in the low carbohydrate-high protein score [9].

Our systematic review did not address macronutrient quality of the diets, specifically the quality of CHO and fat, which along with total macronutrient quantities and proportions, explains the effects of diet on cardiovascular risk [56]. The replacement nutrient is central to these effects. When foods high in CHO are avoided and replaced with high protein foods, reliance on animal protein sources becomes necessary since most foods with significant amounts of plant protein are also high in CHO (e.g. legumes). This reliance on animal protein will result in a greater intake of both total and especially saturated fat leading to higher serum HDL and LDL cholesterol over time. Substitution of saturated fat with polyunsaturated fats reduces coronary heart disease risk [57], [58], while substitution with high glycaemic index CHO increases risk [59]. LDL-cholesterol is a causal risk factor for heart disease and reducing LDL cholesterol has been shown to be effective in reducing risk of heart disease irrespective of the presence of prior heart disease, age, sex, hypertension and diabetes [60]–[62]. Mendelian randomisation studies have demonstrated a 54% reduction in coronary heart disease risk per 1 mmol/l lower serum LDL cholesterol over a lifetime [62]. Treatment of elevated cholesterol levels reduces coronary heart disease risk, with clinical trials demonstrating a 24% reduction in risk per 1 mmol/l reduction in LDL over 5 years [60]. Furthermore, the role of ultra-processed products in the etiology and treatment of obesity and NCD is a pertinent consideration in this area [63], [64]. The inconsistent changes in blood lipids and markers of diabetes risk with both diets in the trials may be attributable to differences in the quality of macronutrients in the intervention diets, for example, different intakes of saturated fat and/or types of carbohydrates (low or high glycaemic), an issue which was beyond the scope of our review. These inconsistences may also be attributable to participants not fully adhering to the prescribed total macronutrient goals for each of the diets, as evident from the adherence data.

Any dietary guidelines for health should be sustainable in the long-term, specifically in terms of ease of adherence, availability and affordability of foods, as well as social and cultural acceptability. Bearing this in mind, the dietary approach for weight management should be one that is nutritionally sound, not harmful and feasible to maintain over time. Such diets can be tailored to the needs of individuals on the basis of each individual's complete health and risk profile, for example existing lipid abnormalities and comorbidities, as well as food preferences, socioeconomic circumstances and personal and cultural preferences, thereby improving the chances of longer term success. Suitably qualified healthcare professionals should guide the tailoring of dietary advice for individuals. Monitoring and follow-up by a healthcare professional during a dietary weight loss intervention is known to positively affect outcomes [16]. The demonstrated value of combining dietary and other positive lifestyle interventions such as increased physical activity for weight loss and reduction of cardiovascular risk, is also important to keep in mind [65], [66].

### Potential biases in the review process

Three prominent electronic databases were searched and two authors carried out the various steps in the review (screening and selecting, extracting, risk of bias assessment, analysing, collation and interpretation). Although we planned not to include non-English randomised controlled trials, we did not come across any potentially eligible studies that we needed to exclude based on language.

## Conclusions

Trials show weight loss in the short-term irrespective of whether the diet is low CHO or balanced in terms of its macronutrient composition. There is probably little or no difference in weight loss and changes in cardiovascular risk factors up to two years of follow-up when overweight and obese adults, with or without type 2 diabetes, are randomised to low CHO diets and isoenergetic balanced weight loss diets.

## Supporting Information

PRISMA Checklist S1 (PDF)

Support Information S1 **A critical summary of existing systematic reviews.**
(DOCX)

Support Information S2 **Forest plots of meta-analyses in overweight and obese adults without type 2 diabetes mellitus.** Figure S2A: Forest plot of low carbohydrate versus balanced diets in overweight and obese adults for body mass index (kg/m^2^) at three to six months. Figure S2B: Forest plot of low carbohydrate versus balanced diets in overweight and obese adults for body mass index (kg/m^2^) at one year. Figure S2C: Forest plot of low carbohydrate versus balanced diets in overweight and obese adults for diastolic blood pressure (mmHg) at three to six months. Figure S2D: Forest plot of low carbohydrate versus balanced diets in overweight and obese adults for diastolic blood pressure (mmHg) at one to two years. Figure S2E: Forest plot of low carbohydrate versus balanced diets in overweight and obese adults for systolic blood pressure (mmHg) at three to six months. Figure S2F: Forest plot of low carbohydrate versus balanced diets in overweight and obese adults for systolic blood pressure (mmHg) at one to two years. Figure S2G: Forest plot of low carbohydrate versus balanced diets in overweight and obese adults for serum LDL cholesterol (mmol/L) at three to six months. Figure S2H: Forest plot of low carbohydrate versus balanced diets in overweight and obese adults for serum LDL cholesterol (mmol/L) at one to two years. Figure S2I: Forest plot of low carbohydrate versus balanced diets in overweight and obese adults for serum HDL cholesterol (mmol/L) at three to six months. Figure S2J: Forest plot of low carbohydrate versus balanced diets in overweight and obese adults for HDL cholesterol (mmol/L) at one to two years. Figure S2K: Forest plot of low carbohydrate versus balanced diets in overweight and obese adults for serum total cholesterol (mmol/L) at three to six months. Figure S2L: Forest plot of low carbohydrate versus balanced diets in overweight and obese adults for serum total cholesterol (mmol/L) at one to two years. Figure S2M: Forest plot of low carbohydrate versus balanced diets in overweight and obese adults for serum triglycerides (mmol/L) at three to six months. Figure S2N: Forest plot of low carbohydrate versus balanced diets in overweight and obese adults for serum triglycerides (mmol/L) at one to two years. Figure S2O: Forest plot of low carbohydrate versus balanced diets in overweight and obese adults for fasting blood glucose (mmol/L) at three to six months. Figure S2P: Forest plot of low carbohydrate versus balanced diets in overweight and obese adults for fasting blood glucose (mmol/L) at one to two years.
(DOCX)

Support Information S3 **Forest plots of meta-analyses in overweight and obese adults with type 2 diabetes mellitus.** Figure S3A: Forest plot of low carbohydrate versus balanced diets in overweight and obese adults with type 2 diabetes mellitus for body mass index (kg/m^2^) at three to six months. Figure S3B: Forest plot of low carbohydrate versus balanced diets in overweight and obese adults with type 2 diabetes mellitus of body mass index (kg/m^2^) at two years. Figure S2C: Forest plot of low carbohydrate versus balanced diets in overweight and obese adults with type 2 diabetes mellitus for glycosylated hemoglobin (%) at three to six months. Figure S3D: Forest plot of low carbohydrate versus balanced diets in overweight and obese adults with type 2 diabetes mellitus for glycolosated hemoglobin (%) at one to two years. Figure S3E: Forest plot of low carbohydrate versus balanced diets in overweight and obese adults with type 2 diabetes mellitus for fasting blood glucose (mmol/L) at three to six months. Figure S3F: Forest plot of low carbohydrate versus balanced diets in overweight and obese adults with type 2 diabetes mellitus for fasting blood glucose (mmol/L) at 15 months. Figure S3G: Forest plot of low carbohydrate versus balanced diets in overweight and obese adults with type 2 diabetes mellitus for diastolic blood pressure (mmHg) at three to six months. Figure S3H: Forest plot of low carbohydrate versus balanced diets in overweight and obese adults with type 2 diabetes mellitus for diastolic blood pressure (mmHg) at one to two years. Figure S3I: Forest plot of low carbohydrate versus balanced diets in overweight and obese adults with type 2 diabetes mellitus for systolic blood pressure (mmHg) at three to six months. Figure S3J: Forest plot of low carbohydrate versus balanced diets in overweight and obese adults with type 2 diabetes mellitus for systolic blood pressure (mmHg) at one to two years. Figure S3K: Forest plot of low carbohydrate versus balanced diets in overweight and obese adults with type 2 diabetes mellitus for LDL cholesterol (mmol/L) at three to six months. Figure S3L: Forest plot of low carbohydrate versus balanced diets in overweight and obese adults with type 2 diabetes mellitus for LDL cholesterol (mmol/L) at one to two years. Figure S3M: Forest plot of low carbohydrate versus balanced diets in overweight and obese adults with type 2 diabetes mellitus for HDL cholesterol (mmol/L) at three to six months. Figure S3N: Forest plot of low carbohydrate versus balanced diets in overweight and obese adults with type 2 diabetes mellitus for HDL cholesterol (mmol/L) at one to two years. Figure S3O: Forest plot of low carbohydrate versus balanced diets in overweight and obese adults with type 2 diabetes mellitus of total cholesterol (mmol/L) at three to six months. Figure S3P: Forest plot of low carbohydrate versus balanced diets in overweight and obese adults with type 2 diabetes mellitus for total cholesterol (mmol/L) at one to two years. Figure S3Q: Forest plot of low carbohydrate versus balanced diets in overweight and obese adults with type 2 diabetes mellitus for triglycerides (mmol/L) at three to six months. Figure S3R: Forest plot of low carbohydrate versus balanced diets in overweight and obese adults with type 2 diabetes mellitus for triglycerides (mmol/L) at one to two years.
(DOCX)
