# Correction: Low Carbohydrate versus Isoenergetic Balanced Diets for Reducing Weight and Cardiovascular Risk: A Systematic Review and Meta-Analysis

**DOI:** 10.1371/journal.pone.0200284

**Published:** 2018-07-02

**Authors:** The PLOS ONE Editors

In response to questions raised about this article [1] following its publication and a criticism and rebuttal that were published in the South African Medical Journal [2, 3], *PLOS ONE* did a full re-evaluation of this article, advised by Editorial Board members and a statistical reviewer. Based on this assessment, the *PLOS ONE* Editors concluded that some corrections and clarifications are needed; these are addressed below. With these amendments, the article meets *PLOS ONE*’s publication criteria and the conclusions of the article stand.

The following errors were noted following publication:

The authors made a typographical error in Table 3. The range of Fat for Balanced should be 20–35% and not 25–35% as in the published table. The cut-offs pertain to criteria for inclusion of studies in the systematic review. These cut-offs were based on the international guidelines, which are referenced in the paper. The authors confirmed that they correctly applied 20% as an inclusion criterion when conducting the study. Although this typographical error does not directly influence the results and conclusions of the systematic review, there are 2 included trials [4, 5] where the control diets had fat ranges that do not comply with the cut-off value that was incorrectly reported in Table 3, and thus they would have been excluded from the review had the incorrect range reported in the original Table 3 been applied. Please see the corrected Table 3 here.

**Table 3:** Cut-off ranges* used to classify the macronutrient goals of treatment and control diets.

	Classifications		
Macronutrients	Low	Balanced	High
Carbohydrate (% of total energy)	< 45	45 to 65	> 65
Fat (% of total energy)	< 25	20 to 35	> 35
Protein (% of total energy)	< 10	10 to 20	> 20

*Established by drawing on macronutrient recommendations from four global institutions and governments [12–15,70].

A reader pointed out that the same study was reported in two articles included in the systematic review, cited in the original article as references 28 and 39 [6, 7]; hence this study was represented in duplicate in the meta-analysis. The two papers, published in the same year, did not reference each other, so it was unclear from the published record that they reported results from the same trial (n = 36), though the trial’s investigators confirmed this point in January 2017 when contacted by the authors. The duplication only affects the meta-analysis reported in Fig 3 and the following sentences in the Results section, where the duplicate paper is represented by reference 39:

“In people without diabetes, eight trials examined the high fat variant [16], [24], [26], [27], [29], [32], [33], [38] and 6 the high protein variant [28], [31], [36], [37], [39], [41].”

“Five trials showed better adherence in the low CHO diet groups [35]–[37], [40], [41] and four trials showed better adherence in the balanced diet group [28], [34], [38], [39].”

**Fig 3:**
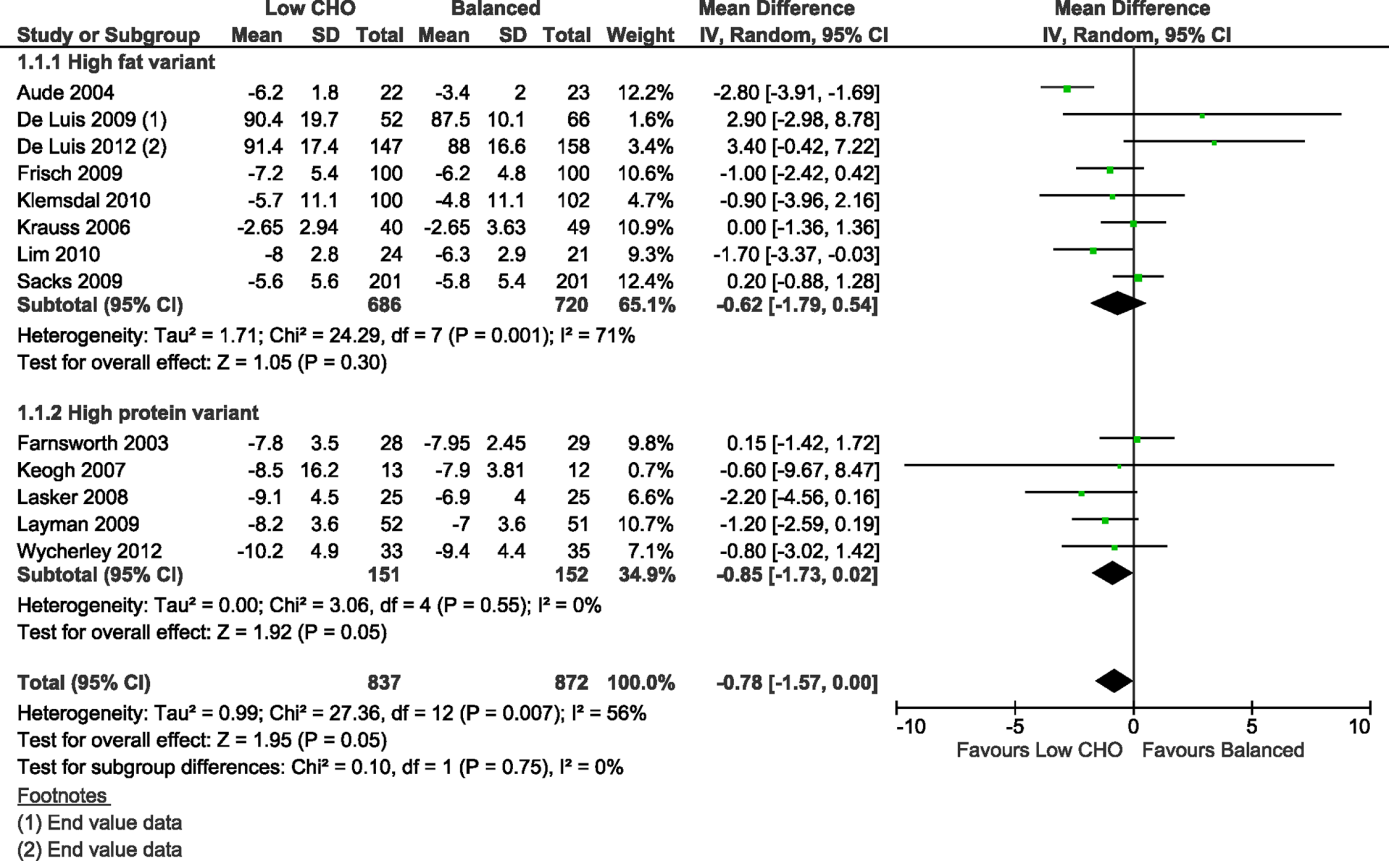
Forest plot of low carbohydrate versus balanced diets in overweight and obese adults for weight loss (kg) at 3–6 months.

With this Correction, the authors provide a new version of Fig 3 in which the meta-analysis has been repeated with the duplicate study removed, as a sensitivity analysis excluding the duplicate trial [7]. The previously published mean difference in weight at 3–6 months in non-diabetic overweight and obese adults, following low carbohydrate diets compared to balanced diets, was -0.74 kg (95%CI -1.49 to 0.01; I^2^ = 53%; n = 1745; 14 trials) [1]. Following the sensitivity analysis, the mean difference in weight was -0.78 kg (95%CI -1.57 to 0.00; I^2^ = 56%; n = 1709; 13 trials), a discrepancy in mean differences of 40 g, as shown in the corrected version of Fig 3.

The authors also provide the following clarifications and additional details in response to the independent *PLOS ONE* re-evaluation:

## The model used in the analyses

The authors used the random effects model, which uses inverse variance weighting. This is a standard meta-analysis model where one anticipates that the true effect estimate may be different between studies [8]. As can be expected with diets, people adhere differently, length of follow up varies, and different methods are used to estimate dietary intake, thus a random effects model is likely the correct model to use in these circumstances. Heterogeneity was estimated with I^2^ and interpreted using standard thresholds [8]. The authors did not quote pooled results if the I^2^ was more than 75%, which indicates substantial heterogeneity. Using these thresholds for the interpretation of I^2^ can be seen as a limitation, since the importance of inconsistency depends on several factors [8]. The observed value of I^2^ depends on magnitude and direction of effects and strength of evidence for heterogeneity, as provided by the P value from the chi-squared test, or a confidence interval for I^2^ [8]. The authors reported the P values from the chi-squared test in the Figures [1]. The authors also took care in interpreting heterogeneity when there was a small number of trials where the evaluation of heterogeneity may be underpowered.

## Discussion of BMI versus weight change in the context of the systematic review

As per their protocol, the authors reported results for total weight change (kg) and body mass index (BMI) (kg/m^2^). Of the 18 included trials, 15 reported on power calculations, of which 10 trials used the weight in their sample size calculation, while none used BMI—meaning most trials were specifically powered to detect differences in weight between the groups over time. For this reason, the authors chose to report the weight change result in their abstract and synthesized weight and BMI findings in the Results section. The BMI results for overweight and obese adults without type 2 diabetes are summarized in Tables 10 and 11 [1] and in the Supporting Information (Figures S2A, S2B, S3A and S3B) [1]. Results for BMI mirrored those for weight change [1].

## Statistical significance, clinical significance, and observed effect

Correct and careful interpretation of statistical tests demands assessing the sizes of estimates of effect and confidence intervals, as well as precise P values (not just if P values are above or below 0.05 or a different threshold) [9]. Confusing statistical significance with the size or importance of an effect has been discussed at length in the peer-reviewed literature [9–11]. Statistical significance does not equate to clinical importance, which requires a meaningful change in an outcome that matters, and in general, refers to the smallest change in an outcome that is considered “important” or “worthwhile” by the practitioner or the patient and/or would result in a change in patient management [12]. Thus, if slightly different inclusion criteria were applied resulting in a few trials being included or excluded in the various meta-analyses [1], the statistical significance of mean differences may have changed, but this does not automatically change the size or importance of the observed differences in effect between the diets being compared.

Body weight typically fluctuates from day to day due to food intake and fluid balance changes, which depend on gender, age and activity level, with diurnal fluctuations of up to 2 kg [13]. Heavier individuals tend to have larger fluctuations in weight [14]. Fig 3 showed that in overweight and obese adults, the average amount of weight lost at 3–6 months with both low carbohydrate weight loss diets and balanced weight loss diets equal in energy, could be regarded as clinically important to overweight and obese adult patients and their practitioners. When the authors compared the average amount of weight lost after following these two diets for 3–6 months, the *difference* in amount of weight loss between the two diets was not clinically important for the overweight and obese patient, at only 780 grams (well within the range of typical daily weight fluctuation). The same applies to BMI at 3–6 months.

The clinical importance of the observed difference in effects on weight and BMI when comparing the two diets, is especially pertinent when considering that the trial participants started with BMIs of 26 kg/m^2^ or greater and were obese on average (mean baseline BMI in both groups was greater than 30 kg/m^2^ in trials that reported baseline BMI). If a weight-loss diet results in only about 780 g more weight loss after 3 to 6 months in an overweight or obese adult compared to another weight-loss diet, one cannot practically and clinically conclude that the first diet is more effective in treating the overweight participant than the second diet.

## Limitation related to assessment of publication bias

The authors assessed publication bias with funnel plots when there were 10 or more studies per outcome [1], as recommended [8]. This was the case for five outcomes in non-diabetic overweight and obese adults in the early follow-up category (3–6 months), as interpreted in the published paper [1]. For all the other outcomes, they could not assess publication bias–an acknowledged limitation. The authors used random-effects meta-analyses, which awards relatively more weight to smaller studies compared to fixed-effect meta-analyses [8]. The use of a random-effects model thus does not offer protection to the extent that a fixed-effect model does, which gives less weight to small studies. Sensitivity analyses comparing findings of random effects versus fixed effects models showed no impacts on the clinical importance of effect estimates.

## Clarifying the use of the Cochran Q test to detect heterogeneity

The Cochran Q (i.e. chi-square) is known to be underpowered to detect heterogeneity, especially for meta-analyses with few studies [8, 15]. For this reason, the authors used a significance level of P<0.1, as stated in the methods [1]. Thus, their methods accounted for the underpowered nature of the chi-square.

Some have argued that since clinical and methodological diversity always occur in a meta-analysis, statistical heterogeneity is inevitable. Consequently, the test for heterogeneity is irrelevant to the choice of analysis. Heterogeneity will always exist whether or not we happen to be able to detect it using a statistical test. Methods have been developed for quantifying inconsistency across studies that move the focus away from testing whether heterogeneity is present to assessing its impact on the meta-analysis. An example of this is the I^2^ test, which is useful since it quantifies inconsistency, and describes the percentage of the variability in effect estimates that is due to heterogeneity rather than sampling error (8). Thus, the authors considered another measure of heterogeneity by using the I^2^ test (1).

The authors assessed and quantified heterogeneity in all meta-analyses, but as occurs often with meta-analysis, some heterogeneity may likely have remained undetected. The potentially undetected heterogeneity, particularly in small meta-analyses, is a limitation. The authors did not apply a fixed-effects model which assumes homogeneity but acknowledged the potential for undetected heterogeneity by employing a random-effects model.

## References

[1] Naude CE, Schoonees A, Senekal M, Young T, Garner P, et al (2014) Low Carbohydrate versus Isoenergetic Balanced Diets for Reducing Weight and Cardiovascular Risk: A Systematic Review and Meta-Analysis. PLoS ONE 9(7): e100652 doi: 10.1371/journal.pone.0100652 25007189 10.1371/journal.pone.0100652 PMC4090010

[2] Harcombe Z, Noakes TD. The universities of Stellenbosch/Cape Town low-carbohydrate diet review: Mistake or mischief?. S Afr Med J. 2016; 106(12):1179–1182. doi: 10.7196/SAMJ.2016.v106.i12.12072 27917760 10.7196/SAMJ.2016.v106.i12.12072

[3] Naude CE, Schoonees A, Senekal M, Garner P, Young T, Volmink J. Reliable systematic review of low-carbohydrate diets shows similar weight-loss effects compared with balanced diets and no cardiovascular risk benefits: Response to methodo-logical criticisms. S Afr Med J. 2017; 107(3):170 doi: 10.7196/SAMJ.2017.v107i3.12382

[4] Sacks FM, Bray GA, Carey VJ, Smith SR, Ryan DH, et al Comparison of Weight-Loss Diets with Different Compositions of Fat, Protein, and Carbohydrates. N Engl J Med. 2009; 360(9):859–873. doi: 10.1056/NEJMoa0804748 19246357 10.1056/NEJMoa0804748 PMC2763382

[5] Keogh JB, Brinkworth GD, Clifton PM. Effects of weight loss on a low-carbohydrate diet on flow-mediated dilatation, adhesion molecules and adiponectin. Br J Nutr. Cambridge University Press; 2007; 98(4): 852–859. doi: 10.1017/S0007114507747815 17490508 10.1017/S0007114507747815

[6] Farnsworth E, Luscombe ND, Noakes M, Wittert G, Argyiou E, et al Effect of a high-protein, energy-restricted diet on body composition, glycemic control, and lipid concentrations in overweight and obese hyperinsulinemic men and women. Amer J Clin Nutr. 2003; 78(1): 31–39. doi: 10.1093/ajcn/78.1.31 12816768 10.1093/ajcn/78.1.31

[7] Luscombe ND, Clifton PM, Noakes M, Farnsworth E, Wittert G (2003) Effect of a high-protein, energy-restricted diet on weight loss and energy expenditure after weight stabilization in hyperinsulinemic subjects. Int J Obes Relat Metab Disord. 2003; 27: 582–590. doi: 10.1038/sj.ijo.0802270 12704402 10.1038/sj.ijo.0802270

[8] Higgins D, Green S, editors. Cochrane Handbook for Systematic Reviews of Interventions Version 5.1.1 [updated March 2011]. London: John Wiley & Sons, Ltd; 2011

[9] Greenland S, Senn SJ, Rothman KJ, Carlin JB, Poole C, Goodman SN, et al Statistical tests, P values, confidence intervals, and power: a guide to misinterpretations. Eur J Epidemiol. 2016; 31:337–350. doi: 10.1007/s10654-016-0149-3 27209009 10.1007/s10654-016-0149-3 PMC4877414

[10] Sterne JA, Smith GD. Sifting the evidence-what's wrong with significance tests? Phys Ther. 2001;81(8):1464–9. doi: 10.1093/ptj/81.8.1464 28206639 10.1093/ptj/81.8.1464

[11] Gardner MA, Altman DG. Confidence intervals rather than P values: estimation rather than hypothesis testing. Br Med J. 1986;292:746–750 3082422 10.1136/bmj.292.6522.746 PMC1339793

[12] Guyatt G. Rennie D. Meade MO, Cook DJ. Users' Guide to Medical Literature: A Manual for Evidence-Based Clinical Practice, 2nd Edition 2008.

[13] Lohman T, Roche AF, Martorell R. Anthropometric Standardization Reference Manual. Human Kinetics: Champaign, IL, 1988

[14] Truesdale KP, Stevens J, Lewis CE, Schreiner PJ, Loria CM, Cai J. Changes in risk factors for cardiovascular disease by baseline weight status in young adults who maintain or gain weight over 15 years: the CARDIA study. Int J Obes (Lond). 2006 9; 30(9): 1397–1407. doi: 10.1038/sj.ijo.0803307 16534519 10.1038/sj.ijo.0803307 PMC3234682

[15] Hardy RJ, Thompson SG. Detecting and describing heterogeneity in meta-analysis. Stat Med. 1998;17(8):841–56. Epub 1998/05/22. 9595615 10.1002/(sici)1097-0258(19980430)17:8<841::aid-sim781>3.0.co;2-d

