# Supplementary material for: Low Carbohydrate versus Isoenergetic Balanced Diets for Reducing Weight and Cardiovascular Risk: A Systematic Review and Meta-Analysis
Source: PLoS One. 2014 Jul 9;9(7):e100652. doi: 10.1371/journal.pone.0100652 (PMC4090010; doi:10.1371/journal.pone.0100652)
Supplement: Support Information S1 — A critical summary of existing systematic reviews. (DOCX) [file pone.0100652.s002.docx]

**Low carbohydrate versus isoenergetic balanced diets for reducing weight and cardiovascular risk:**

**a systematic review and meta-analysis**

**Support Information S1: A critical summary of existing systematic reviews**

# Introduction

We sought to answer this question from existing systematic reviews, using the Cochrane Overview of Systematic Reviews methods [[1](#_ENREF_1)].

# Objectives

To examine the available synthesised evidence on dietary macronutrient manipulation and cardiovascular outcomes and risk factors. A secondary objective was to identify any methodological deficiencies in existing published systematic reviews that could be addressed should we need to undertake a fresh systematic review to answer the question.

# Criteria for considering systematic reviews for inclusion

## **Types of studies:**

Systematic reviews published in English, reporting on dietary macronutrient manipulation and cardiovascular outcomes and risk factors that included experimental (randomised controlled trials, quasi-randomised controlled trials or before-after studies) or observational studies in humans. To avoid excluding any relevant systematic reviews due to the inconsistent terminology that is used to describe similar dietary approaches (for example, low carbohydrate diets are also called high protein diets), we included any type of dietary macronutrient manipulation in relation to cardiovascular health.

## Types of participants

General population; individuals who are overweight or obese, have diabetes, glucose intolerance or insulin resistance, cardiovascular conditions or risk factors such as hypertension and dyslipidaemia. Systematic reviews were excluded when pregnant or lactating women or children younger than 18 years were the focus.

## **Types of treatments or exposures**

Dietary regimens purporting various dietary macronutrient manipulations, excluding: the combination of dietary macronutrient manipulation with any other interventions (e.g. exercise, pharmacological, surgical) where the effect of diet alone could not be assessed; interventions with an exclusive focus on energy restriction; and interventions of specific foods, food groups or food components (e.g. dairy, oats, plant sterols).

## **Types of controls**

A macronutrient manipulation or advice different from the treatment.

## **Types of outcome measures**

All-cause and cardiovascular mortality; cardiovascular morbidity such as first diagnosis of cardiovascular disease (CVD), myocardial infarction (MI), stroke; weight, glycaemic control, blood pressure and circulating lipids levels.

# Search methods for identification of studies

A comprehensive electronic search of *The Cochrane Library* and *Medline* were conducted to identify potential relevant systematic reviews, with the last search on 3 March 2014. *The Cochrane Library*, the *Cochrane Database of Systematic reviews* and *Database of Abstracts of Reviews and Effects* (DARE) database were searched with the following search string: **“(carbohydrate OR fat OR protein) AND diet”** in **Title, Abstract or Keywords.** Medline (via PubMed) was searched using the following search string: “Diet, High-Fat”[Mesh] OR “Diet, Protein-Restricted”[Mesh] OR “Diet, Fat-Restricted”[Mesh] OR “Diet, Carbohydrate-Restricted”[Mesh] OR “Diet Therapy”[Mesh] OR “Ketogenic Diet”[Mesh] OR “Diabetic Diet”[Mesh] OR “Diet, Reducing”[Mesh] Filters: Systematic Reviews; Meta-Analysis*.* Finally, reference lists provided by two nutrition experts were hand-searched.

# Data extraction and tabulation

## Selection of studies

Two authors (CN and AS) independently screened the titles and abstracts of all search results and identified potentially eligible systematic reviews using the pre-specified eligibility criteria. Where at least one author considered a study to be relevant, the full text document of the article was obtained. Screening of full text articles for final inclusion was divided between the first two authors. Uncertainty was resolved by discussion among review authors. Reasons were provided for excluding full text articles.

## Data extraction and tabulation

Two authors (CN and AS) simultaneously extracted information compiled tables of this information to display the scope of the questions that were addressed. The following was extracted: types of participants, description of participants, number and types of studies included, pre-specified treatment/exposure and comparator and outcomes reported relevant to this project.

# Findings

## Description of studies

The search results and selection process are detailed in Figure 1. Briefly, we screened 851 records and retrieved and screened 105 full-text articles, after which we included 50 reviews.


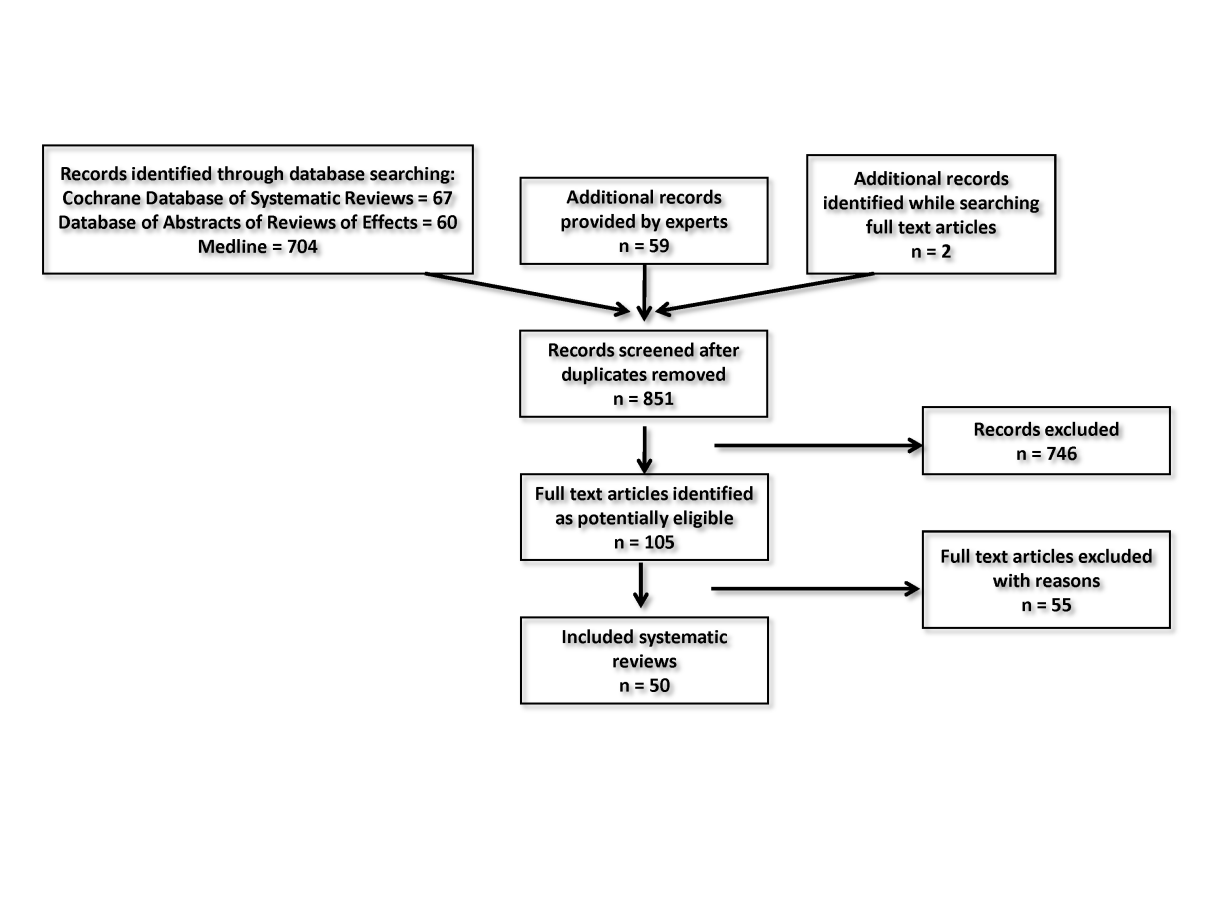


**Figure 1: Flowchart of search results and selection process**

## **Included studies**

We grouped the data extracted from the 50 included reviews into four tables according to the macronutrient emphasis of the treatment diets, as follows: 9 concentrated solely on the effect or association of carbohydrate intake (Table 1); 28 concentrated only on the effect or association of fat intake (including Mediterranean diet and cholesterol lowering diets) (Table 2); 6 were concerned only with protein intake (Table 3); and 7 examined the effect or association of two or all three macronutrients (Table 4).

The 50 included reviews examined various questions around dietary macronutrient composition, weight and cardiovascular health, but did not explicitly address the question we set out to answer. Five major constraints to interpreting the reviews in relation to our question were identified (Table 5). In the large majority of reviews the treatment and control diets were not explicitly defined, with the complete macronutrient profiles of the comparison diets being unclear (e.g. only provided the goal for on one of the macronutrients). The outcomes of these reviews could thus not be ascribed to any particular intervention diet in terms of its macronutrient composition. Inconsistent definitions for commonly used terms to describe and define intervention diets such as “low”, “moderate” or “usual” were also problematic. Table 5 also outlines how the identified limitations were addressed in our systematic review.

## **Excluded studies**

A total of 55 full-text articles were excluded with reasons (Table 6).

**Table 1: Systematic reviews with emphasis on the effect or association of carbohydrate intake**

| **Types of participants** | **First author, year Date of last search** | **Description of participants** | **Number and types of studies included** | | | | **Pre-specified treatment/exposure** | **Pre-specified comparator** | **Outcomes reported relevant to this project** | | |
| --- | --- | --- | --- | --- | --- | --- | --- | --- | --- | --- | --- |
|  |  |  | CT | Cohort | CS | Other |  |  | CV events or incidence of disease | Weight-related | CVD risk factors |
| Adults with CV risk factors or disease | Bueno 2013 [[2](#_ENREF_2)]; August 2012 | ≥18 years; BMI ≥ 27.5 kg/ m² | 13 |  |  |  | Restricted energy, ‘very low CHO ketogenic diet’ (≤50 g CHO/d or ≤10% of TE from CHO) | Restricted energy diet; low fat diet (<30% of TE from fat) |  | weight | LDL, HDL, TG, SBP, DBP, HbA1c, glucose, insulin |
|  | Hu 2012 [[3](#_ENREF_3)]; 20 June 2011 | Adults ≥18 years | 23 |  |  |  | Low CHO diet (≤ 45% of TE) | Low fat diet (≤ 30% of TE) |  | weight | TC, LDL, HDL, TG, SBP, DBP, glucose, insulin |
|  | Nordmann 2006 [[4](#_ENREF_4)]; 28 February 2005 | People ≥16 years; BMI ≥25 kg/m^2^ | 5 |  |  |  | Low CHO diet (maximum intake of 60 g/day) without energy restriction | Low fat diet (maximum of 30% of the daily TE from fat) with energy restriction |  | weight | TC, LDL, HDL, TG, SBP, DBP, glucose, insulin |
| Adults with DM or impaired glucose control | Castaneda-Gonzalez 2011 [[5](#_ENREF_5)]; 1 January 2000 to 1 January 2010 | T2 DM; ≥18 years | 8 |  |  |  | Restricted CHO diet defined as maximum of 130g /d | Non-restricted CHO diet; Excluded trials where the quality of the CHO differed between groups |  | weight | TC, LDL, HDL, TG, HbA1C |
|  | Dyson 2008 [[6](#_ENREF_6)]; March 2007 | T2 DM | 6 |  |  |  | Reduced CHO diets | NR |  | weight, BMI | TC, LDL, HDL, TG, HbA1c |
|  | Kirk 2008 [[7](#_ENREF_7)]; April 2006 | T2 DM; ≥19 years | 13 |  |  |  | Restricted CHO diet (4 - 45% of TE) | “Non-restricted CHO diet” |  | weight | TC, LDL, HDL, TG, HbA1c, glucose |
| Not reported or other | Bravata 2003 [[8](#_ENREF_8)] ; 15 February 2003 | Adults, not pregnant | 94 |  |  |  | One of the following interventions: low CHO, ketogenic, higher protein or higher fat diets; Data to calculate g/day of CHO and TE/day have to be reported | NR |  | weight, BMI | TC, LDL, HDL, TG, SBP, fasting glucose |
|  | Santos 2012 [[9](#_ENREF_9)]; March 2011 | Adults >18 years; Excluded all diabetics, COPD, cancer, epilepsy, menopause, pregnant women | 13 |  |  |  | Low CHO diet as defined by the primary study author | Baseline conditions |  | weight, BMI | TC, LDL, HDL, TG SBP, DBP, HbA1c, glucose, insulin |
|  | Shikany 2011 [[10](#_ENREF_10)]; April 2010 | NR | 4 |  |  |  | Modified CHO diets by reducing the amount of CHO and improving the quality of CHO using the concepts glycaemic index and glycaemic load | “Standard energy restricted and reduced fat diets” |  | weight, BMI |  |

BMI = body mass index; CHO = carbohydrate; CS = cross-sectional ; CT = clinical trials; CV = cardiovascular; CVD = cardiovascular disease; DBP = diastolic blood pressure; HbA1c = glycosylated haemoglobin; HDL = high density lipoprotein cholesterol; LDL = low density lipoprotein cholesterol; NR = not reported; SBP = systolic blood pressure; T2 DM = type II diabetes mellitus; TC = total cholesterol; TE = total energy; TG = triglycerides

**Table 2: Systematic reviews focussing only on the effect or association of fat intake (including Mediterranean diet and cholesterol lowering diets)**

| **Types of participants** | **First author, year Date of last search** | **Description of participants** | **Number and types of studies included** | | | | **Pre-specified treatment/exposure** | **Pre-specified comparator** | **Outcomes reported relevant to this project** | | |
| --- | --- | --- | --- | --- | --- | --- | --- | --- | --- | --- | --- |
|  |  |  | CT | Cohort | CS | Other |  |  | CV events or incidence of disease | Weight-related | CVD risk factors |
| Adults with CV risk factors or disease | Buckland 2008 [[11](#_ENREF_11)]; July 2007 | Adults | 11 | 3 | 7 |  | MD as a whole, not restricting the MD definition to specific food groups or nutrients | NR |  | weight, BMI |  |
|  | Hooper 2012 [[12](#_ENREF_12)]; June 2010 | Adults (≥18 years) at any risk of CVD (with or without existing CVD); Excluded acutely ill, pregnant & lactating women | 48 |  |  |  | Intention to reduce or modify dietary fat or cholesterol; Reduced fat diet (<30% of TE from fat, and at least partially replace the energy lost with CHO (simple or complex), protein or fruit and vegetables) or modified fat diet (≥30% of TE from fat, and included higher levels of MUFAs or PUFAs than a ’usual’ diet) | Usual or control diet or reduced total fat | Total & CV mortality, CVD events | weight, BMI | TC, LDL, HDL, TG, SBP, DBP |
|  | Nordmann 2011 [[13](#_ENREF_13)]; January 2011 | Overweight/obese patients with at least 1CV risk factor or established CAD | 6 |  |  |  | MD defined as having moderate fat intake (main sources of added fat were olive oil and nuts), rich in vegetables, and low in red meat (with poultry and fish replacing beef and lamb) | Low fat diet: ≤ 30% of TE from fat |  | weight, BMI | TC, LDL, HDL, TG, SBP, DBP, glucose, insulin |
|  | Pirozzo 2003 [[14](#_ENREF_14)]; February 2002 | ≥18 years, BMI >25 kg/m^2^ | 6 |  |  |  | Low fat | “Another type of weight-reducing diet” |  | weight, BMI | TC, LDL, HDL, TG, BP, HbA1c, glucose |
|  | Smart 2011 [[15](#_ENREF_15)]; February 2010 | Otherwise healthy adults (≥18 years) with acquired (not familial) hypercholesterol-aemia | 0 |  |  |  | Any low fat dietary intervention (e.g. low fat and low SFA diets) intended to lower serum TC or LDL, or to raise HDL | Usual care, calorie-restricted diets or pharmacological interventions | All-cause mortality, CVD events |  | TC, LDL, HDL, TG, |
| Adults with DM or impaired glucose control | Garg 1998 [[16](#_ENREF_16)]; NR | T2 DM | 9 |  |  |  | High MUFA | High CHO |  | weight | TC, LDL, HDL, TG, SBP, DBP, HbA1c, glucose, insulin |
|  | Schwingshackl 2011 [[17](#_ENREF_17)]; May 2011 | Adults, abnormal glucose metabolism, BMI >25 kg/m^2^ | 9 |  |  |  | High MUFA, with MUFA >12% of TE | Low MUFA, that were differentiated to be: low fat diets (total fat ≤ 30%, SFA ≤ 7–10%); low glycaemic index diets; high glycaemic index diets; high-protein diets; control diets (total fat content >30% and/or SFA ≥10%) |  |  | HbA1c, glucose, insulin |
|  | Winters 2008 [[18](#_ENREF_18)]; February 2007 | T2 DM on statin therapy | 6, but 0 on T2 DM |  |  |  | Low fat diet plus statin therapy | Only statin therapy |  |  | LDL |
| Adults with and without DM or impaired glucose control | Cao 2009 [[19](#_ENREF_19)]; 2007 | Diabetics and non-diabetics | 30 |  |  |  | Moderate fat cholesterol-lowering diet rich in unsaturated fat | Low fat diets, isoenergetic to control | CHD incidence |  | TC, LDL, HDL, TG |
|  | Esposito 2010 [[20](#_ENREF_20)]; 30 November 2009 | Diabetics or people at risk of diabetes | 9 | 2 | 4 | 2 | Extent of adherence to a MD; Adherence to MD was assessed as a score, based on the monthly frequency intake of food groups that are common to the MD | NR | Total & CV mortality, CVD events, diabetes incidence |  | HDL, TG, SBP, HbA1C, glucose, insulin |
| Not reported or other | Astrup 2000 [[21](#_ENREF_21)]; July 1999 | Non-diabetics | 16 |  |  |  | Ad libitum low fat diets | Habitual diet or a medium fat diet ad libitum |  | weight |  |
|  | Brunner 1997 [[22](#_ENREF_22)]; July 1993 | Free living adults | 17 |  |  |  | Diets aimed at changing patterns of fat, sodium, or fibre consumption | NR |  |  | TC, DBP |
|  | Clarke 1997 [[23](#_ENREF_23)]; NR | Healthy volunteers | 395 |  |  |  | Dietary intake of fatty acids and cholesterol | Isoenergetic diet compared to the treatment group |  |  | TC, LDL, HDL |
|  | Esposito 2011 [[24](#_ENREF_24)]; January 2010 | NR | 16 |  |  |  | MD as an dietary pattern | NR |  | weight, BMI |  |
|  | Hooper 2012 [[25](#_ENREF_25)]; June 2010 | Apparently healthy adults and children; from any country | 33 | 10 |  |  | Reduce total fat intake (in terms of % TE from fat, or reducing total gram fat/day); at least 26 weeks | “Usual diet” |  | weight, BMI | TC, LDL, HDL, TG, DBP, SBP |
|  | Kastorini 2010 [[26](#_ENREF_26)]; December 2009 | NR | 21 | 3 | 11 |  | MD | NR | Total & CV mortality, CAD incidence | weight, BMI | TC, LDL, HDL, TG, DBP, HbA1c, glucose |
|  | Kastorini 2011 [[27](#_ENREF_27)]; 30 April 2010 | NR | 35 | 2 | 13 |  | MD, including all food groups of MD | NR |  |  | HDL, TG, SBP, DBP, glucose |
|  | Psaltopoulou 2013[[28](#_ENREF_28)]; 31 October 2012 | NR |  | 7 | 2 | 13 | Adherence to MD measured by variety of scores | NR | Stroke |  |  |
|  | Rees 2013 [[29](#_ENREF_29)]; September 2012 | ≥18 years; healthy adults and adults at high risk of CVD; Excluded trials where >25% of participants had CV events previously or where >25% participants had T2 DM | 11 |  |  |  | “MD-style dietary pattern”, having at least 2 components of the following: (1) high MUFA:SFA, (2) low to moderate red wine intake, (3) high legume intake, (4) high grains and cereal intake, (5) high fruit and vegetable intake, (6) low intake of meat and meat products, with increased intake of fish, (7) moderate milk and dairy products intake | No intervention or minimal intervention (e.g. leaflet to follow a dietary pattern with no person-to-person contact) | All-cause & CV mortality, diabetes incidence |  | TC, LDL, HDL, TG, DBP, SBP, HbA1c |
|  | Schwingshackl 2013 [[30](#_ENREF_30)]; March 2013 | Overweight or obese patients; not with previous CV event | 32 |  |  |  | Low fat diet (≤30% of TE); ≥12 months | High fat diet (>30% of TE) |  | weight | TC, LDL, HDL, TG |
|  | Shafiq 2010 [[31](#_ENREF_31)]; 1 June 2008 | Children and adults with familial hypercholeste-rolaemia | 11 |  |  |  | Cholesterol-lowering diet or any other dietary intervention intended to lower total and LDL cholesterol | Could be compared to anything as long as the only difference between the treatment and control groups was the diet | Mortality, incidence of IHD and other CVD |  | TC, LDL, HDL, TG |
|  | Shah 2007 [[32](#_ENREF_32)]; August 2006 | Adults | 10 |  |  |  | High CHO diets, with participants’ weight to remain stable throughout trial | Isoenergetic high-*cis*-MUFA diets, with participants’ weight to remain stable throughout trial |  |  | SBP, DBP |
|  | Siri-Tarino 2010 [[33](#_ENREF_33)]; 17 September 2009 | Generally healthy adults |  | 2 |  |  | Dietary consumption of SFA was given scores, with the higher scores (4^th^ quartile) being the treatment group | Lower SFA scores (1^st^ quartile) | Incidence of CHD and other CVD events |  |  |
|  | Skeaff 2009 [[34](#_ENREF_34)]; NR |  | Unclear | 2 |  |  | High dietary fat exposure was assessed using fatty acid biomarkers | Low dietary fat exposure | Total mortality, fatal and non-fatal CVD events |  |  |
|  | Sofi 2010 [[35](#_ENREF_35)]; June 2010 | NR |  | 18 |  |  | Higher adherence to MD, as measured with a priori adherence score | 2 point lower adherence to MD, as measured with a priori adherence score | CVD mortality, CVD incidence |  |  |
|  | Tang 1998 [[36](#_ENREF_36)]; <1996 | Free living | 19 |  |  |  | Individualised dietary advice to modify fat intake falling into one of the following 4 categories: Step 1 AHA diet with <30% fat of TE and 8 to 10% SFA of TE; Step 2 AHA diet with <30% of TE and ≤7% SFA of TE; Diet to increase PUFA:SFA with little or no change in total fat; Low total fat without changing the proportions of different fats consumed | NR |  |  | TC |
|  | Yu-Poth 1999 [[37](#_ENREF_37)]; 1997 | Free living; healthy people and people at high risk for CVD | 37 |  |  |  | One of the National Cholesterol Education Program’s diets, namely: Step I diet ≤30% fat of TE and ≤10% SFA as TE; Step II diet ≤ 7% SFA of TE | NR | CVD incidence | weight | TC, LDL, HDL, TG |
|  | Wu 2013 [[38](#_ENREF_38)]; 31 August 2012 | Premenopausal and postmenopausal women without CVD or cancer | 8 |  |  |  | Low fat diet (<30% of TE) with at least partial replacement of energy lost with CHO ((simple or complex), protein,or fruit and vegetables; ≥4 weeks | “Usual diet” |  |  | TC, LDL, HDL, TG, |

AHA = American Heart Association; BMI = body mass index; BP = blood pressure; CAD = coronary artery disease; CHD = coronary heart disease; CHO = carbohydrate; CS = cross-sectional; CT = clinical trials; CV= cardiovascular; CVD = cardiovascular disease; DBP = diastolic blood pressure; DM = diabetes mellitus; HbA1c = glycosylated haemoglobin; HDL = high density lipoprotein cholesterol; IHD = ischemic heart disease; LDL = low density lipoprotein cholesterol; MD = Mediterranean diet; MUFA = monounsaturated fatty acids; NR = not reported; PUFA = polyunsaturated fatty acids; SBP = systolic blood pressure; SFA = saturated fatty acids; TC = total cholesterol; T2 DM = type 2 diabetes mellitus; TE = total energy; TG = triglycerides

**Table 3: Systematic reviews with emphasis on the effect or association of protein intake**

| **Types of participants** | **First author, year Date of last search** | **Description of participants** | **Number and types of studies included** | | | | **Pre-specified treatment/exposure** | **Pre-specified comparator** | **Outcomes reported relevant to this project** | | |
| --- | --- | --- | --- | --- | --- | --- | --- | --- | --- | --- | --- |
|  |  |  | CT | Cohort | CS | Other |  |  | CV events or incidence of disease | Weight-related | CVD risk factors |
| Adults with DM or impaired glucose control | Dong 2013 [[39](#_ENREF_39)]; August 2012 | Adults; T2 DM | 9 |  |  |  | High protein diet (>20% of TE); >4 weeks | At least 5% less protein than treatment group |  | weight, BMI | LDL, HDL, TG, DBP, SBP, HbA1c, glucose |
| Not reported or other | Altorf-van der Kuil 2010 [[40](#_ENREF_40)]; June 2010 | Adults | 20 |  |  | 26 | Higher protein intake | Lower protein intake | Incidence of hypertension |  | SBP, DBP |
|  | Halton 2004 [[41](#_ENREF_41)]; NR | NR | 48 |  |  |  | Higher protein | Lower protein |  | weight |  |
|  | Lepe 2011 [[42](#_ENREF_42)]; NR | NR | 8 |  |  |  | High protein diet | A “conventional” energy restricted diet or a low fat, high CHO diet |  | weight, BMI |  |
|  | Rebholz 2012 [[43](#_ENREF_43)]; April 2011 | Normal, overweight and obese adults (≥19 years) | 40 |  |  |  | Increased protein intake | Increased intake of CHO, fat and/or another source of protein | Incidence of hypertension | weight, BMI | SBP, DBP |
|  | Santesso 2012 [[44](#_ENREF_44)]; July 2011 | Adults >18 years in general population; with or without hyperlipidaemia, hypertension, metabolic syndrome; ≥80% of participants must without chronic illness requiring dietary intervention (e.g. diabetes or chronic renal disease) | 74 |  |  |  | High protein diet, irrespective of whether the aim is weight loss or not; Excluded special meal replacements or supplements | Low protein diet using 5% in TE | Death; CVD events; incidence of diabetes, renal disease (or need dialysis), cancer, or bone fractures; other adverse events | weight, BMI | TC, LDL, HDL, TG, DBP, SBP,HbA1c, glucose, insulin |

BMI = body mass index; DM = diabetes mellitus; CHO = carbohydrate; CS = cross-sectional; CT = clinical trials; CV= cardiovascular; CVD = cardiovascular disease; DBP = diastolic blood pressure; HbA1c = glycosylated haemoglobin; HDL = high density lipoprotein cholesterol; LDL = low density lipoprotein cholesterol; NR = not reported; SBP = systolic blood pressure; TE = total energy; T2 DM = type 2 diabetes mellitus

**Table 4: Systematic reviews with emphasis on the effect or association of two or all three macronutrients**

| **Types of participants** | **First author, year Date of last search** | **Description of participants** | **Number and types of studies included** | | | | **Pre-specified treatment/exposure** | **Pre-specified comparator** | **Outcomes reported relevant to this project** | | |
| --- | --- | --- | --- | --- | --- | --- | --- | --- | --- | --- | --- |
|  |  |  | CT | Cohort | CS | Other |  |  | CV events or incidence of disease | Weight-related | CVD risk factors |
| Adults with CV risk factors or disease | Hession 2009 [[45](#_ENREF_45)]; January 2000 to March 2007 | Adults (>18 years) with BMI ≥28 kg/m^2^ | 13 |  |  |  | Low CHO, high protein diets that was either one of the following: high protein ‘ketogenic’ diet, where CHO was <40 g/day, irrespective of TE; or low CHO diets (≤60 g/day) | Low fat, high CHO diets (≤30% TE from fat) with 600 kCal deficit; or “Healthy eating advice” |  | weight | TC, LDL, HDL, TG, SBP, DBP, HbA1c, glucose, insulin |
| Adults with DM or impaired glucose control | Kodama 2009 [[46](#_ENREF_46)]; 2007 | Adults with T2 DM | 22 |  |  |  | Low fat, high CHO diets; CHO quality between treatment and control group must be similar | High fat, low CHO diet, with isoenergetic energy and similar protein when compared to treatment group |  | BMI | TC, LDL, HDL, TG, HbA1c, glucose |
|  | Ajala 2013 [[47](#_ENREF_47)]; August 2011 | Adults (≥18 years); T2 DM | 20 |  |  |  | One or more of the following diets: low CHO, high CHO, high protein, vegetarian or vegan, low glycaemic, high fibre, or MD; duration >6 months | “Any control diet” |  | weight | TC, LDL, HDL, TG, HbA1c, glucose |
| Not Reported or Other | Krieger 2006 [[48](#_ENREF_48)]; 18 September 2005 | Adults (≥ 19 years) | 87 |  |  |  | Low CHO, high protein diets with ≥4200 kJ/day | Low fat diets (higher CHO and lower protein content compared to treatment group |  | weight |  |
|  | Noto 2013 [[49](#_ENREF_49)]; 12 September 2012 | NR |  | 17 |  |  | Low CHO intake; duration ≥12 months | High CHO intake | All-cause & CV mortality, CVD events | weight |  |
|  | Schwingshackl 2013 [[50](#_ENREF_50)]; August 2012 | NR | 15 |  |  |  | High protein (≥ 25% of TE) , low fat (≤ 30% of TE) diet; ≥12 months | Low protein (≤ 20% of TE), low fat (≤ 30% of TE) diet |  | weight | TC, LDL, HDL, TG, SBP, DBP, HbA1c, glucose, insulin |
|  | Wycherley 2012 [[51](#_ENREF_51)]; 12 May 2011 | ≥18 years, with any BMI | 23 |  |  |  | Energy-restricted (weight-loss), high protein diets; duration ≥4 weeks; Excluded: had a concurrent structured exercise programme, TE <4184 kJ/d | Standard protein, low-fat (≤30% of TE from fat) weight loss diets; difference between treatment and control group must be <1250 kJ/d for TE (isoenergetic) and ≤10% of TE from fat |  | weight | TC, LDL, HDL, TG, SBP, DBP, glucose, insulin |

BMI = body mass index; CHO = carbohydrates; CS = cross-sectional; CT = clinical trials; CV= cardiovascular; CVD = cardiovascular disease; DBP = diastolic blood pressure; HbA1c = glycated haemoglobin; HDL = high density lipoprotein cholesterol; LDL = low density lipoprotein cholesterol; SBP = systolic blood pressure; TC = total cholesterol; TE = total energy; TG = triglycerides; T2 DM = type 2 diabetes mellitu

**Table 5: Main limitations identified in existing systematic reviews that served as constraints to interpretation of the evidence and what we did to address them in our review**

| **What answering the research question requires** | **Why was it identified as a limitation in existing reviews?** | **What we did to address identified limitations in our review** |
| --- | --- | --- |
| Explicit definition of treatment and control diets with complete macronutrient profile | If unclear, any effects seen on weight loss and CVD risk factors cannot be attributed to a well-defined intervention diet compared to a well-defined control diet | Used explicit cut-off ranges for macronutrients for treatment and control diets; the complete macronutrient profile of intervention diets had to be available (proportions of total energy intake) |
| Recommended energy intake in treatment and control groups needs to be similar | If different, any effects seen on weight loss and CVD risk factors would be confounded by total energy intake | Only included isoenergetic diet comparisons |
| Co-interventions, such as drugs given as part of the intervention, or recommendations for exercise, need to be similar in the comparison groups | If different, any effects on CVD risk factors could be confounded by co-interventions | Only included interventions with a diet component alone, or combined interventions that were similar to prevent confounding by co-interventions |
| Appropriate study design for the question | Methodological heterogeneity: some reviews included both controlled and uncontrolled trials | Only included randomised controlled trials |
| Meaningful and comparable follow-up in trials needs to be considered | Outcomes of trials with different follow-ups were pooled; generalized conclusions about weight loss may be skewed by early changes; or follow-up may be insufficient to detect CVD risk factor changes | Only included studies with 12 weeks or more follow-up; and outcomes were grouped by defined timepoints |

CVD: cardiovascular disease

**Table 6: Excluded systematic reviews with reasons for exclusion**

| Study ID  (First author and year) | Reason for exclusion |
| --- | --- |
| Almeida 2009 [[52](#_ENREF_52)] | Not English |
| Astrup 2000 [[53](#_ENREF_53)] | Duplicate |
| Astrup 2002 [[54](#_ENREF_54)] | Not a systematic review |
| Avenell 2004a[[55](#_ENREF_55)] | No control group |
| Avenell, 2004b[[56](#_ENREF_56)] | No control group |
| Ayyad 2000 [[57](#_ENREF_57)] | Intervention has a focus on energy restriction rather than macronutrient composition |
| Babio 2009 [[58](#_ENREF_58)] | Not a systematic review |
| Bautista 2005 [[59](#_ENREF_59)] | Not a systematic review |
| Bray 2008 [[60](#_ENREF_60)] | Not a systematic review |
| Brown 1996 [[61](#_ENREF_61)] | Intervention has a focus on energy restriction rather than macronutrient composition |
| Brown 2009 [[62](#_ENREF_62)] | Effect of macronutrient manipulation alone not assessed |
| Brunner 2007 [[63](#_ENREF_63)] | No control group |
| Clauss 2002 [[64](#_ENREF_64)] | Participants not adults |
| Dansinger 2007 [[65](#_ENREF_65)] | Intervention has a focus on energy restriction rather than macronutrient composition |
| de Lorgeril 2007 [[66](#_ENREF_66)] | Not a systematic review |
| Dick 2004 [[67](#_ENREF_67)] | Not a systematic review |
| Ebrahim 2011 [[68](#_ENREF_68)] | Effect of diet alone not assessed |
| Franz 2004 [[69](#_ENREF_69)] | Effect of diet alone not assessed |
| Gibson 2006 [[70](#_ENREF_70)] | Participants not adults |
| Giugliano 2008 [[71](#_ENREF_71)] | Not a systematic review |
| Hadi 2005 [[72](#_ENREF_72)] | Not a systematic review |
| Howell 1997 [[73](#_ENREF_73)] | Not directly relevant to the question |
| Johnson 2010 [[74](#_ENREF_74)] | Participants not adults |
| Kastorini 2009 [[75](#_ENREF_75)] | Not a systematic review |
| Kesten 2011 [[76](#_ENREF_76)] | Effect of macronutrient manipulation alone not assessed |
| Lara 2011 [[77](#_ENREF_77)] | Focus not on macronutrient composition |
| McArthur 1998 [[78](#_ENREF_78)] | Effect of macronutrient manipulation alone not assessed |
| Miller 1997 [[79](#_ENREF_79)] | Intervention has a focus on energy restriction rather than macronutrient composition |
| Nield 2007 [[80](#_ENREF_80)] | Focus not on macronutrient composition |
| Nilsson 2009 [[81](#_ENREF_81)] | Not a systematic review |
| Nordmann 1994 [[82](#_ENREF_82)] | Not English |
| Norris 2004 [[83](#_ENREF_83)] | Intervention has a focus on energy restriction rather than macronutrient composition |
| Obarzanek 1996 [[84](#_ENREF_84)] | No control group |
| Orozco 2008 [[85](#_ENREF_85)] | Effect of diet alone not assessed |
| Oude Luttikhuis 2009 [[86](#_ENREF_86)] | Effect of diet alone not assessed |
| Panagiotakos 2004 [[87](#_ENREF_87)] | Not a systematic review |
| Pirozzo 2002 [[88](#_ENREF_88)] | Duplicate |
| Poustie 2001 [[89](#_ENREF_89)] | Duplicate |
| Proietti 2009 [[90](#_ENREF_90)] | Not English |
| Saraswat 2012 [[91](#_ENREF_91)] | Only a protocol |
| Schooff 2003 [[92](#_ENREF_92)] | Not a systematic review |
| Serra-Majem 2006 [[93](#_ENREF_93)] | Not a systematic review |
| Siebenhofer 2011 [[94](#_ENREF_94)] | Control was not a dietary intervention |
| Snethen 2006 [[95](#_ENREF_95)] | Effect of macronutrient manipulation alone not assessed |
| Sodlerlund 2009 [[96](#_ENREF_96)] | Effect of macronutrient manipulation alone not assessed |
| Sofi 2009 [[97](#_ENREF_97)] | Not a systematic review |
| Studer 2005 [[98](#_ENREF_98)] | Focus not on macronutrient composition |
| Summerbell 2008 [[99](#_ENREF_99)] | Duplicate |
| Tawaramoto 2005 [[100](#_ENREF_100)] | Not English |
| Tsai 2005 [[101](#_ENREF_101)] | Focus not on macronutrient composition |
| Tyrovolas 2010 [[102](#_ENREF_102)] | Not a systematic review |
| Walker 2010 [[103](#_ENREF_103)] | Not a systematic review |
| Walsh 1995 [[104](#_ENREF_104)] | Not a systematic review |
| Yamaoka 2013 [[105](#_ENREF_105)] | Focus not on macronutrient composition |
| Young 2012 [[106](#_ENREF_106)] | Effect of macronutrient manipulation alone not assessed |

**References**

1. Higgins D, Green S, editors (2011) Cochrane Handbook for Systematic Reviews of Interventions Version 5.1.1 [updated March 2011]. London: John Wiley & Sons, Ltd.

2. Bueno NB, de Melo IS, de Oliveira SL, da Rocha Ataide T (2013) Very-low-carbohydrate ketogenic diet v. low-fat diet for long-term weight loss: a meta-analysis of randomised controlled trials. Br J Nutr 110: 1178-1187.

3. Hu T, Mills KT, Yao L, Demanelis K, Eloustaz M, et al. (2012) Effects of low-carbohydrate diets versus low-fat diets on metabolic risk factors: a meta-analysis of randomized controlled clinical trials. American Journal of Epidemiology 176 Suppl 7: S44-54.

4. Nordmann AJ, Nordmann A, Briel M, Keller U, Yancy WS, Jr., et al. (2006) Effects of low-carbohydrate vs low-fat diets on weight loss and cardiovascular risk factors: a meta-analysis of randomized controlled trials. Arch Intern Med 166: 285-293.

5. Castaneda-Gonzalez LM, Bacardi Gascon M, Jimenez Cruz A (2011) Effects of low carbohydrate diets on weight and glycemic control among type 2 diabetes individuals: a systemic review of RCT greater than 12 weeks. Nutr Hosp 26: 1270-1276.

6. Dyson PA (2008) A review of low and reduced carbohydrate diets and weight loss in type 2 diabetes. Journal of Human Nutrition & Dietetics 21: 530-538.

7. Kirk JK, Graves DE, Craven TE, Lipkin EW, Austin M, et al. (2008) Restricted-carbohydrate diets in patients with type 2 diabetes: a meta-analysis. Journal of the American Dietetic Association 108: 91-100.

8. Bravata DM, Sanders L, Huang J, Krumholz HM, Olkin I, et al. (2003) Efficacy and safety of low-carbohydrate diets: a systematic review. JAMA 289: 1837-1850.

9. Santos FL, Esteves SS, da Costa Pereira A, Yancy WS, Jr., Nunes JP (2012) Systematic review and meta-analysis of clinical trials of the effects of low carbohydrate diets on cardiovascular risk factors. Obes Rev 13: 1048-1066.

10. Shikany JM, Desmond R, McCubrey R, Allison DB (2011) Meta-analysis of studies of a specific delivery mode for a modified-carbohydrate diet. Journal of Human Nutrition & Dietetics 24: 525-535.

11. Buckland G, Bach A, Serra-Majem L (2008) Obesity and the Mediterranean diet: a systematic review of observational and intervention studies. Obes Rev 9: 582-593.

12. Hooper L, Summerbell CD, Thompson R, Sills D, Roberts FG, et al. (2012) Reduced or modified dietary fat for preventing cardiovascular disease. Cochrane Database of Systematic Reviews 16.

13. Nordmann AJ, Suter-Zimmermann K, Bucher HC, Shai I, Tuttle KR, et al. (2011) Meta-analysis comparing Mediterranean to low-fat diets for modification of cardiovascular risk factors. American Journal of Medicine 124: 841-851.

14. Pirozzo S, Summerbell C, Cameron C, Glasziou P (2003) Should we recommend low-fat diets for obesity? Obes Rev 4: 83-90.

15. Smart NA, Marshall BJ, Daley M, Boulos E, Windus J, et al. (2011) Low-fat diets for acquired hypercholesterolaemia. Cochrane Database of Systematic Reviews 16.

16. Garg A (1998) High-monounsaturated-fat diets for patients with diabetes mellitus: a meta-analysis. Am J Clin Nutr 67: 577S-582S.

17. Schwingshackl L, Strasser B, Hoffmann G (2011) Effects of monounsaturated fatty acids on glycaemic control in patients with abnormal glucose metabolism: a systematic review and meta-analysis. Annals of Nutrition & Metabolism 58: 290-296.

18. Winters SM, Visser H, Steerneman AH, Thomas G, Bots ML, et al. (2008) Is there a need for dietary measures to further reduce LDL cholesterol in patients with type II diabetes mellitus on statin therapy? Prim Care Diabetes 2: 51-54.

19. Cao Y, Mauger DT, Pelkman CL, Zhao G, Townsend SM, et al. (2009) Effects of moderate (MF) versus lower fat (LF) diets on lipids and lipoproteins: a meta-analysis of clinical trials in subjects with and without diabetes. J Clin Lipidol 3: 19-32.

20. Esposito K, Maiorino MI, Ceriello A, Giugliano D (2010) Prevention and control of type 2 diabetes by Mediterranean diet: a systematic review. Diabetes Res Clin Pract 89: 97-102.

21. Astrup A, Grunwald GK, Melanson EL, Saris WH, Hill JO (2000) The role of low-fat diets in body weight control: a meta-analysis of ad libitum dietary intervention studies. Int J Obes Relat Metab Disord 24: 1545-1552.

22. Brunner E, White I, Thorogood M, Bristow A, Curle D, et al. (1997) Can dietary interventions change diet and cardiovascular risk factors? A meta-analysis of randomized controlled trials. American Journal of Public Health 87: 1415-1422.

23. Clarke R, Frost C, Collins R, Appleby P, Peto R (1997) Dietary lipids and blood cholesterol: quantitative meta-analysis of metabolic ward studies. BMJ 314: 112-117.

24. Esposito K, Kastorini CM, Panagiotakos DB, Giugliano D (2011) Mediterranean diet and weight loss: meta-analysis of randomized controlled trials. Metab Syndr Relat Disord 9: 1-12.

25. Hooper L, Abdelhamid A, Moore HJ, Douthwaite W, Skeaff CM, et al. (2012) Effect of reducing total fat intake on body weight: systematic review and meta-analysis of randomised controlled trials and cohort studies. BMJ 345: e7666.

26. Kastorini CM, Milionis HJ, Goudevenos JA, Panagiotakos DB (2010) Mediterranean diet and coronary heart disease: is obesity a link? - A systematic review. Nutr Metab Cardiovasc Dis 20: 536-551.

27. Kastorini CM, Milionis HJ, Esposito K, Giugliano D, Goudevenos JA, et al. (2011) The effect of Mediterranean diet on metabolic syndrome and its components: a meta-analysis of 50 studies and 534,906 individuals. J Am Coll Cardiol 57: 1299-1313.

28. Psaltopoulou T, Sergentanis TN, Panagiotakos DB, Sergentanis IN, Kosti R, et al. (2013) Mediterranean Diet, Stroke, Cognitive Impairment, and Depression: A Meta-Analysis. Annals of Neurology 74: 580-591.

29. Rees K, Hartley L, Flowers N, Clarke A, Hooper L, et al. (2013) 'Mediterranean' dietary pattern for the primary prevention of cardiovascular disease. Cochrane Database Syst Rev 8: CD009825.

30. Schwingshackl L, Hoffmann G (2013) Comparison of effects of long-term low-fat vs high-fat diets on blood lipid levels in overweight or obese patients: a systematic review and meta-analysis. J Acad Nutr Diet 113: 1640-1661.

31. Shafiq N, Singh M, Kaur S, Khosla P, Malhotra S (2010) Dietary treatment for familial hypercholesterolaemia. Cochrane Database of Systematic Reviews 20.

32. Shah M, Adams-Huet B, Garg A (2007) Effect of high-carbohydrate or high-cis-monounsaturated fat diets on blood pressure: a meta-analysis of intervention trials. Am J Clin Nutr 85: 1251-1256.

33. Siri-Tarino PW, Sun Q, Hu FB, Krauss RM (2010) Meta-analysis of prospective cohort studies evaluating the association of saturated fat with cardiovascular disease. Am J Clin Nutr 91: 535-546.

34. Skeaff CM, Miller J (2009) Dietary fat and coronary heart disease: summary of evidence from prospective cohort and randomised controlled trials. Annals of Nutrition & Metabolism 55: 173-201.

35. Sofi F, Abbate R, Gensini GF, Casini A (2010) Accruing evidence on benefits of adherence to the Mediterranean diet on health: an updated systematic review and meta-analysis. Am J Clin Nutr 92: 1189-1196.

36. Tang JL, Armitage JM, Lancaster T, Silagy CA, Fowler GH, et al. (1998) Systematic review of dietary intervention trials to lower blood total cholesterol in free-living subjects. BMJ 316: 1213-1220.

37. Yu-Poth S, Zhao G, Etherton T, Naglak M, Jonnalagadda S, et al. (1999) Effects of the National Cholesterol Education Program's Step I and Step II dietary intervention programs on cardiovascular disease risk factors: a meta-analysis. Am J Clin Nutr 69: 632-646.

38. Wu L, Ma D, Walton-Moss B, He Z (2014) Effects of low-fat diet on serum lipids in premenopausal and postmenopausal women: a meta-analysis of randomized controlled trials. Menopause 21: 89-99.

39. Dong JY, Zhang ZL, Wang PY, Qin LQ (2013) Effects of high-protein diets on body weight, glycaemic control, blood lipids and blood pressure in type 2 diabetes: meta-analysis of randomised controlled trials. Br J Nutr 110: 781-789.

40. Altorf – van der Kuil W, Engberink MF, Brink EJ, van Baak MA, Bakker SJL, et al. (2010) Dietary Protein and Blood Pressure: A Systematic Review. PLoS ONE [Electronic Resource] 5: e12102.

41. Halton TL, Hu FB (2004) The effects of high protein diets on thermogenesis, satiety and weight loss: a critical review. J Am Coll Nutr 23: 373-385.

42. Lepe M, Bacardi Gascon M, Jimenez Cruz A (2011) Long-term efficacy of high-protein diets: a systematic review. Nutr Hosp 26: 1256-1259.

43. Rebholz CM, Friedman EE, Powers LJ, Arroyave WD, He J, et al. (2012) Dietary protein intake and blood pressure: a meta-analysis of randomized controlled trials. American Journal of Epidemiology 176 Suppl 7: S27-43.

44. Santesso N, Akl EA, Bianchi M, Mente A, Mustafa R, et al. (2012) Effects of higher- versus lower-protein diets on health outcomes: a systematic review and meta-analysis. European Journal of Clinical Nutrition 66: 780-788.

45. Hession M, Rolland C, Kulkarni U, Wise A, Broom J (2009) Systematic review of randomized controlled trials of low-carbohydrate vs. low-fat/low-calorie diets in the management of obesity and its comorbidities. Obes Rev 10: 36-50.

46. Kodama S, Saito K, Tanaka S, Maki M, Yachi Y, et al. (2009) Influence of fat and carbohydrate proportions on the metabolic profile in patients with type 2 diabetes: a meta-analysis. Diabetes Care 32: 959-965.

47. Ajala O, English P, Pinkney J (2013) Systematic review and meta-analysis of different dietary approaches to the management of type 2 diabetes. Am J Clin Nutr 97: 505-516.

48. Krieger JW, Sitren HS, Daniels MJ, Langkamp-Henken B (2006) Effects of variation in protein and carbohydrate intake on body mass and composition during energy restriction: a meta-regression 1. Am J Clin Nutr 83: 260-274.

49. Noto H, Goto A, Tsujimoto T, Noda M (2013) Low-carbohydrate diets and all-cause mortality: a systematic review and meta-analysis of observational studies. PLoS One 8: e55030.

50. Schwingshackl L, Hoffmann G (2013) Long-term effects of low-fat diets either low or high in protein on cardiovascular and metabolic risk factors: a systematic review and meta-analysis. Nutrition Journal 12.

51. Wycherley TP, Moran LJ, Clifton PM, Noakes M, Brinkworth GD (2012) Effects of energy-restricted high-protein, low-fat compared with standard-protein, low-fat diets: a meta-analysis of randomized controlled trials. Am J Clin Nutr 96: 1281-1298.

52. Almeida JC, Rodrigues TC, Silva FM, Azevedo MJ (2009) [Systematic review of weight loss diets: role of dietary components]. Arq Bras Endocrinol Metabol 53: 673-687.

53. Astrup A, Ryan L, Grunwald GK, Storgaard M, Saris W, et al. (2000) The role of dietary fat in body fatness: evidence from a preliminary meta-analysis of ad libitum low-fat dietary intervention studies. British Journal of Nutrition 83: S25-32.

54. Astrup A, Buemann B, Flint A, Raben A (2002) Low-fat diets and energy balance: how does the evidence stand in 2002? Proceedings of the Nutrition Society 61: 299-309.

55. Avenell A, Broom J, Brown TJ, Poobalan A, Aucott L, et al. (2004) Systematic review of the long-term effects and economic consequences of treatments for obesity and implications for health improvement. Health Technol Assess 8: 1-182.

56. Avenell A, Brown TJ, McGee MA, Campbell MK, Grant AM, et al. (2004) What are the long-term benefits of weight reducing diets in adults? A systematic review of randomized controlled trials. Journal of Human Nutrition & Dietetics 17: 317-335.

57. Ayyad C, Andersen T (2000) Long-term efficacy of dietary treatment of obesity: a systematic review of studies published between 1931 and 1999. Obes Rev 1: 113-119.

58. Babio N, Bullo M, Salas-Salvado J (2009) Mediterranean diet and metabolic syndrome: the evidence. Public Health Nutrition 12: 1607-1617.

59. Bautista MC, Engler MM (2005) The Mediterranean diet: is it cardioprotective? Prog Cardiovasc Nurs 20: 70-76.

60. Bray GA (2008) Lifestyle and pharmacological approaches to weight loss: efficacy and safety. Journal of Clinical Endocrinology & Metabolism 93: S81-88.

61. Brown SA, Upchurch S, Anding R, Winter M, Ramirez G (1996) Promoting weight loss in type II diabetes. Diabetes Care 19: 613-624.

62. Brown T, Avenell A, Edmunds LD, Moore H, Whittaker V, et al. (2009) Systematic review of long-term lifestyle interventions to prevent weight gain and morbidity in adults. Obes Rev 10: 627-638.

63. Brunner EJ, Rees K, Ward K, Burke M, Thorogood M (2007) Dietary advice for reducing cardiovascular risk. Cochrane Database of Systematic Reviews 17.

64. Clauss SB, Kwiterovich PO (2002) Long-term safety and efficacy of low-fat diets in children and adolescents. Minerva Pediatr 54: 305-313.

65. Dansinger ML, Tatsioni A, Wong JB, Chung M, Balk EM (2007) Meta-analysis: the effect of dietary counseling for weight loss. Ann Intern Med 147: 41-50.

66. de Lorgeril M, Salen P (2007) Modified cretan Mediterranean diet in the prevention of coronary heart disease and cancer: An update. World Review of Nutrition & Dietetics 97: 1-32.

67. Dick JJ (2004) Weight loss interventions for adult obesity: evidence for practice. Worldviews Evid Based Nurs 1: 209-214.

68. Ebrahim S, Taylor F, Ward K, Beswick A, Burke M, et al. (2011) Multiple risk factor interventions for primary prevention of coronary heart disease. Cochrane Database of Systematic Reviews: CD001561.

69. Franz MJ (2004) Effectiveness of weight loss and maintenance interventions in women. Curr Diab Rep 4: 387-393.

70. Gibson LJ, Peto J, Warren JM, dos Santos Silva I (2006) Lack of evidence on diets for obesity for children: a systematic review. International Journal of Epidemiology 35: 1544-1552.

71. Giugliano D, Ceriello A, Esposito K (2008) Are there specific treatments for the metabolic syndrome? Am J Clin Nutr 87: 8-11.

72. Hadi S, Jensen GL (2005) Efficacy of a low-carbohydrate diet for short-term weight loss. Nutrition in Clinical Practice 20: 17-20.

73. Weber CL, Matthews HS (2008) Food-miles and the relative climate impacts of food choices in the United States. Environ Sci Technol 42: 3508-3513.

74. Johnson ST, Newton AS, Chopra M, Buckingham J, Huang TT, et al. (2010) In search of quality evidence for lifestyle management and glycemic control in children and adolescents with type 2 diabetes: A systematic review. BMC Pediatr 10: 97.

75. Kastorini CM, Panagiotakos DB (2009) Dietary patterns and prevention of type 2 diabetes: from research to clinical practice; a systematic review. Curr Diabetes Rev 5: 221-227.

76. Kesten JM, Griffiths PL, Cameron N (2011) A systematic review to determine the effectiveness of interventions designed to prevent overweight and obesity in pre-adolescent girls. Obes Rev 12: 997-1021.

77. Lara M, Amigo H (2011) [What kind of intervention has the best results to reduce the weight in overweighted or obese adults?]. Archivos Latinoamericanos de Nutricion 61: 45-54.

78. McArthur DB (1998) Heart healthy eating behaviors of children following a school-based intervention: a meta-analysis. Issues Compr Pediatr Nurs 21: 35-48.

79. Miller WC, Koceja DM, Hamilton EJ (1997) A meta-analysis of the past 25 years of weight loss research using diet, exercise or diet plus exercise intervention. Int J Obes Relat Metab Disord 21: 941-947.

80. Nield L, Moore HJ, Hooper L, Cruickshank JK, Vyas A, et al. (2007) Dietary advice for treatment of type 2 diabetes mellitus in adults. Cochrane Database of Systematic Reviews 18.

81. Nilsson PM (2009) [Mediterranean diet protects the heart. A new systemic review supports the diet recommendations]. Lakartidningen 106.

82. Nordmann A (1994) [Mediterranean or low-fat diets to reduce cardiovascular risk?]. Praxis 100: 1283-1288.

83. Norris SL, Zhang X, Avenell A, Gregg E, Bowman B, et al. (2004) Long-term effectiveness of lifestyle and behavioral weight loss interventions in adults with type 2 diabetes: a meta-analysis. American Journal of Medicine 117: 762-774.

84. Obarzanek E, Velletri PA, Cutler JA (1996) Dietary protein and blood pressure. JAMA 275: 1598-1603.

85. Orozco LJ, Buchleitner AM, Gimenez-Perez G, Roque IFM, Richter B, et al. (2008) Exercise or exercise and diet for preventing type 2 diabetes mellitus. Cochrane Database of Systematic Reviews 16.

86. Oude Luttikhuis H, Baur L, Jansen H, Shrewsbury VA, O'Malley C, et al. (2009) Interventions for treating obesity in children. Cochrane Database of Systematic Reviews 21.

87. Panagiotakos DB, Pitsavos C, Polychronopoulos E, Chrysohoou C, Zampelas A, et al. (2004) Can a Mediterranean diet moderate the development and clinical progression of coronary heart disease? A systematic review. Med Sci Monit 10: 23.

88. Pirozzo S, Summerbell C, Cameron C, Glasziou P (2002) Advice on low-fat diets for obesity. Cochrane Database of Systematic Reviews 2.

89. Poustie VJ, Rutherford P (2001) Dietary treatment for familial hypercholesterolaemia. Cochrane Database of Systematic Reviews 2.

90. Proietti AR, del Balzo V, Dernini S, Donini LM, Cannella C (2009) [Mediterranean diet and prevention of non-communicable diseases: scientific evidences]. Ann Ig 21: 197-210.

91. Saraswat A, Jayasinghe R, Sweeny AL (2012) Carbohydrate or fat‐restricted diets for obesity (protocol). Cochrane Database of Systematic Reviews: John Wiley & Sons, Ltd.

92. Schooff M (2003) Are low-fat diets better than other weight-reducing diets in achieving long-term weight loss? American Family Physician 67: 507-508.

93. Serra-Majem L, Roman B, Estruch R (2006) Scientific evidence of interventions using the Mediterranean diet: a systematic review. Nutr Rev 64: S27-47.

94. Siebenhofer A, Jeitler K, Berghold A, Waltering A, Hemkens LG, et al. (2011) Long-term effects of weight-reducing diets in hypertensive patients. Cochrane Database of Systematic Reviews 7.

95. Snethen JA, Broome ME, Cashin SE (2006) Effective weight loss for overweight children: a meta-analysis of intervention studies. J Pediatr Nurs 21: 45-56.

96. Sodlerlund A, Fischer A, Johansson T (2009) Physical activity, diet and behaviour modification in the treatment of overweight and obese adults: a systematic review. Perspect Public Health 129: 132-142.

97. Sofi F (2009) The Mediterranean diet revisited: evidence of its effectiveness grows. Curr Opin Cardiol 24: 442-446.

98. Studer M, Briel M, Leimenstoll B, Glass TR, Bucher HC (2005) Effect of different antilipidemic agents and diets on mortality: a systematic review. Arch Intern Med 165: 725-730.

99. Summerbell CD, Cameron C, Glasziou PP (2008) WITHDRAWN: Advice on low-fat diets for obesity. Cochrane Database of Systematic Reviews 16.

100. Tawaramoto K, Kaku K (2005) [Treatment for patients with impaired glucose tolerance]. Nihon Rinsho 2: 423-427.

101. Tsai AG, Wadden TA (2005) Systematic review: an evaluation of major commercial weight loss programs in the United States. Ann Intern Med 142: 56-66.

102. Tyrovolas S, Panagiotakos DB (2010) The role of Mediterranean type of diet on the development of cancer and cardiovascular disease, in the elderly: a systematic review. Maturitas 65: 122-130.

103. Walker KZ, O'Dea K, Gomez M, Girgis S, Colagiuri R (2010) Diet and exercise in the prevention of diabetes. Journal of Human Nutrition & Dietetics 23: 344-352.

104. Walsh JM, Grady D (1995) Treatment of hyperlipidemia in women. JAMA 274: 1152-1158.

105. Yamaoka K, Tango T (2012) Effects of lifestyle modification on metabolic syndrome: a systematic review and meta-analysis. BMC Med 10: 138.

106. Young MD, Morgan PJ, Plotnikoff RC, Callister R, Collins CE (2012) Effectiveness of male-only weight loss and weight loss maintenance interventions: a systematic review with meta-analysis. Obes Rev 13: 393-408.
