# Supplementary material for: Low Carbohydrate versus Isoenergetic Balanced Diets for Reducing Weight and Cardiovascular Risk: A Systematic Review and Meta-Analysis
Source: PLoS One. 2014 Jul 9;9(7):e100652. doi: 10.1371/journal.pone.0100652 (PMC4090010; doi:10.1371/journal.pone.0100652)
Supplement: Support Information S2 — Forest plots of meta-analyses in overweight and obese adults without type 2 diabetes mellitus. Figure S2A: Forest plot of low carbohydrate versus balanced diets in overweight and obese adults for body mass index (kg/m2) at three to six months. Figure S2B: Forest plot of low carbohydrate versus balanced diets in overweight and obese adults for body mass index (kg/m2) at one year. Figure S2C: Forest plot of low carbohydrate versus balanced diets in overweight and obese adults for diastolic blood pressure (mmHg) at three to six months. Figure S2D: Forest plot of low carbohydrate versus balanced diets in overweight and obese adults for diastolic blood pressure (mmHg) at one to two years. Figure S2E: Forest plot of low carbohydrate versus balanced diets in overweight and obese adults for systolic blood pressure (mmHg) at three to six months. Figure S2F: Forest plot of low carbohydrate versus balanced diets in overweight and obese adults for systolic blood pressure (mmHg) at one to two years. Figure S2G: Forest plot of low carbohydrate versus balanced diets in overweight and obese adults for serum LDL cholesterol (mmol/L) at three to six months. Figure S2H: Forest plot of low carbohydrate versus balanced diets in overweight and obese adults for serum LDL cholesterol (mmol/L) at one to two years. Figure S2I: Forest plot of low carbohydrate versus balanced diets in overweight and obese adults for serum HDL cholesterol (mmol/L) at three to six months. Figure S2J: Forest plot of low carbohydrate versus balanced diets in overweight and obese adults for HDL cholesterol (mmol/L) at one to two years. Figure S2K: Forest plot of low carbohydrate versus balanced diets in overweight and obese adults for serum total cholesterol (mmol/L) at three to six months. Figure S2L: Forest plot of low carbohydrate versus balanced diets in overweight and obese adults for serum total cholesterol (mmol/L) at one to two years. Figure S2M: Forest plot of low carbohydrate versus balanced diets in over [file pone.0100652.s003.docx]

**Low carbohydrate versus isoenergetic balanced diets for reducing weight and cardiovascular risk:**

**a systematic review and meta-analysis**

**Support Information S2: Forest plots of meta-analyses in overweight and obese adults without type 2 diabetes mellitus**


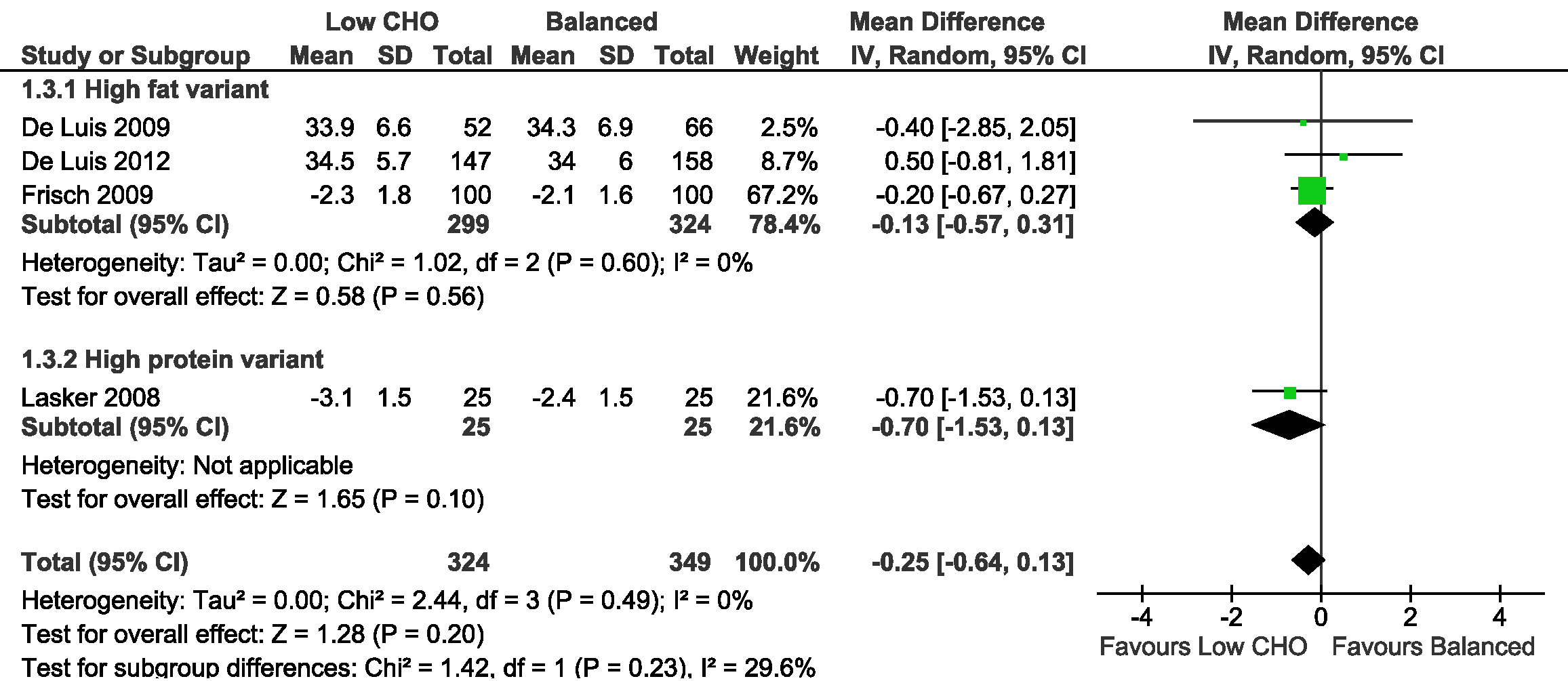


Figure S2A: Forest plot of low carbohydrate versus balanced diets in overweight and obese adults for body mass index (kg/m^2^) at three to six months


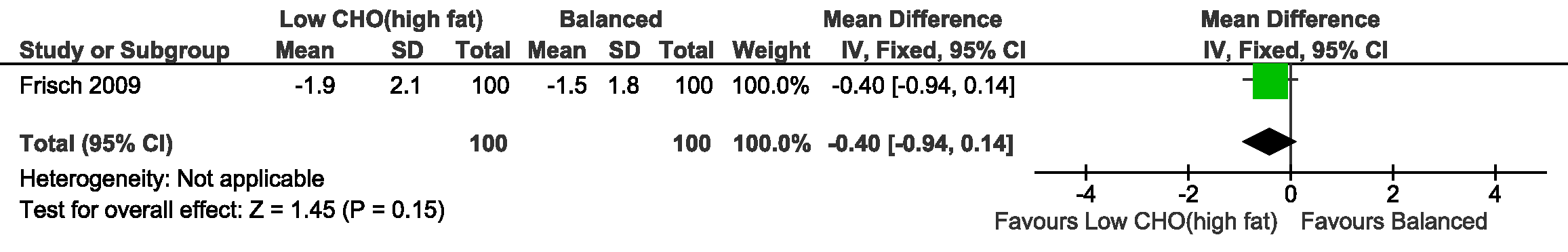


Figure S2B: Forest plot of low carbohydrate versus balanced diets in overweight and obese adults for body mass index (kg/m^2^) at one year


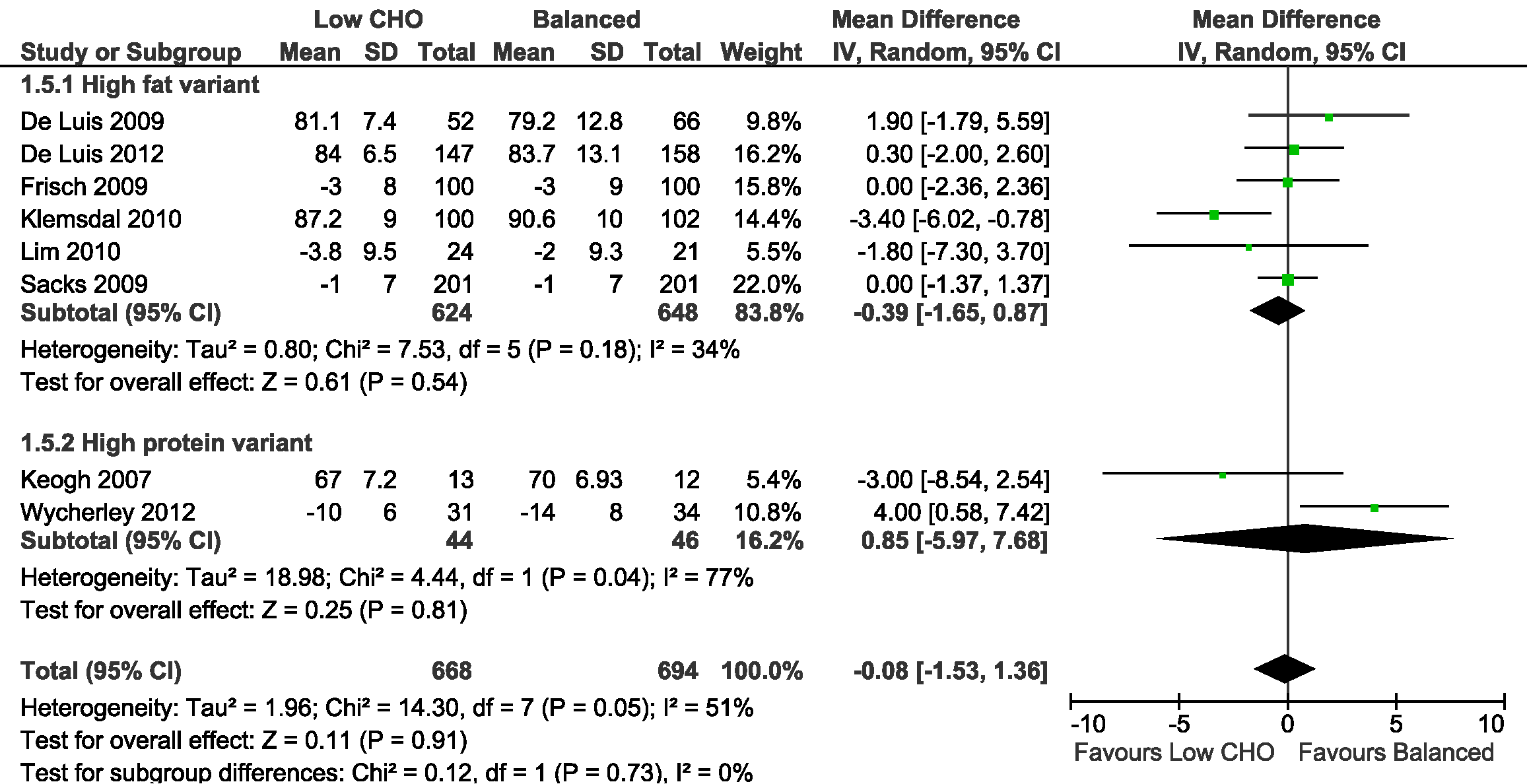


Figure S2C: Forest plot of low carbohydrate versus balanced diets in overweight and obese adults for diastolic blood pressure (mmHg) at three to six months


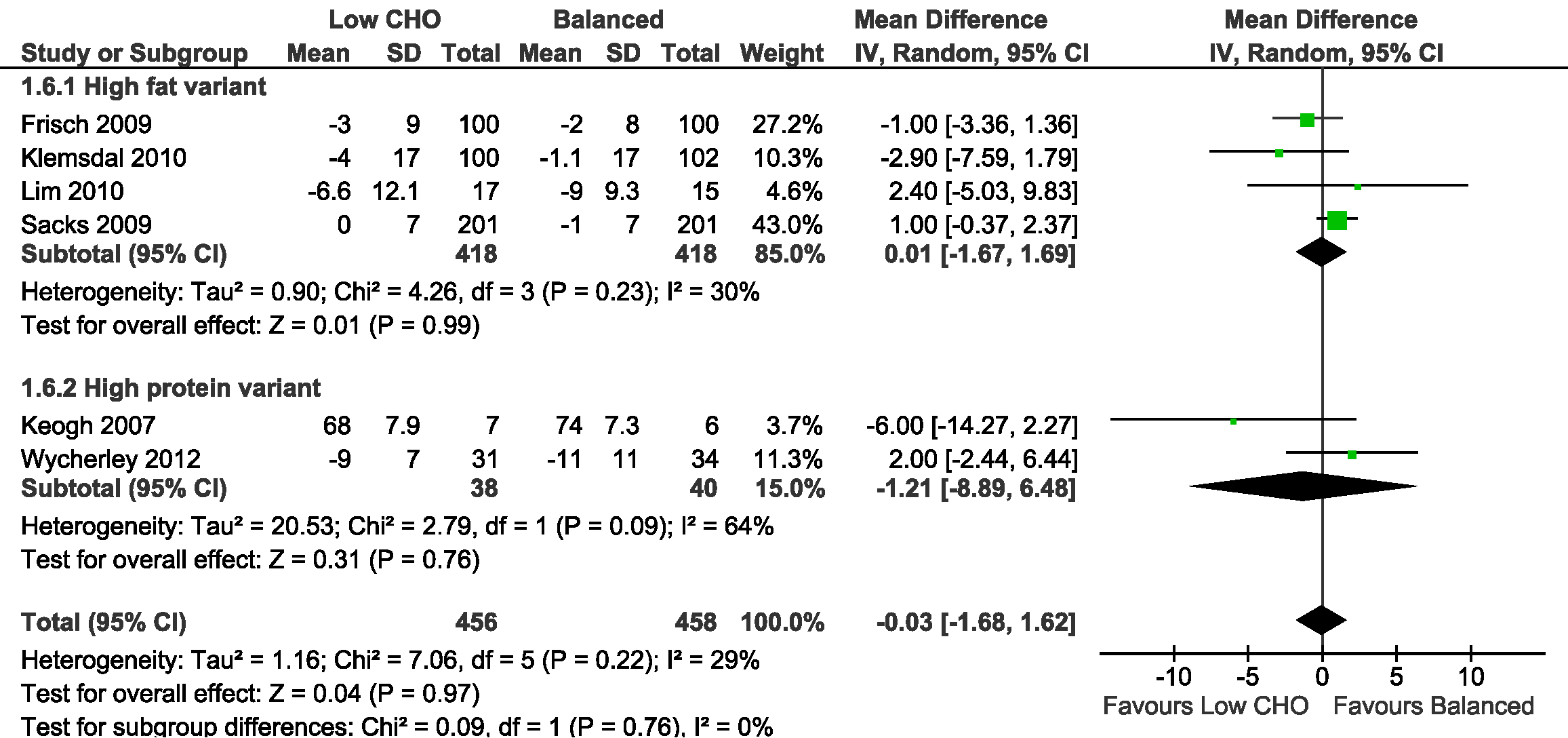


Figure S2D: Forest plot of low carbohydrate versus balanced diets in overweight and obese adults for diastolic blood pressure (mmHg) at one to two years


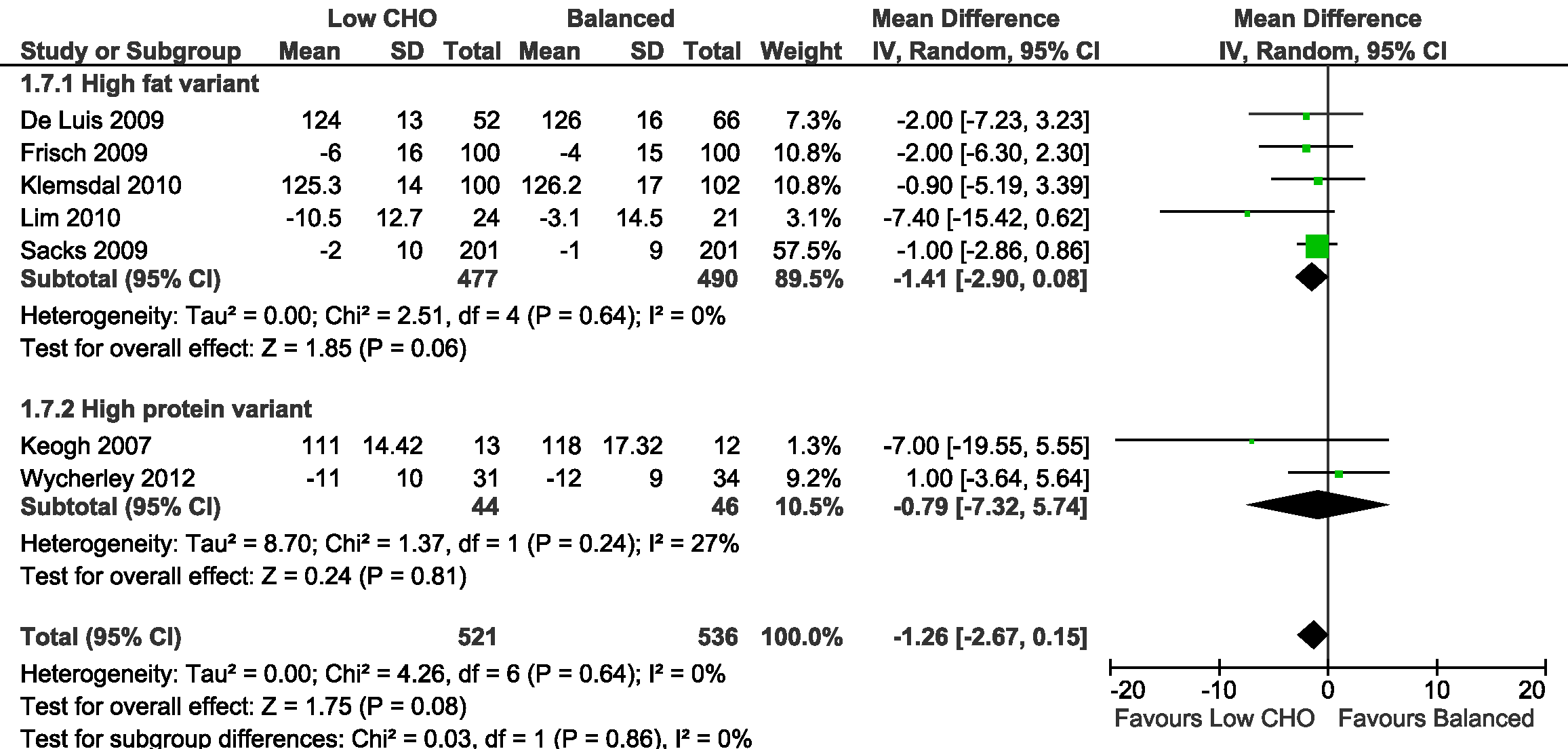


Figure S2E: Forest plot of low carbohydrate versus balanced diets in overweight and obese adults for systolic blood pressure (mmHg) at three to six months


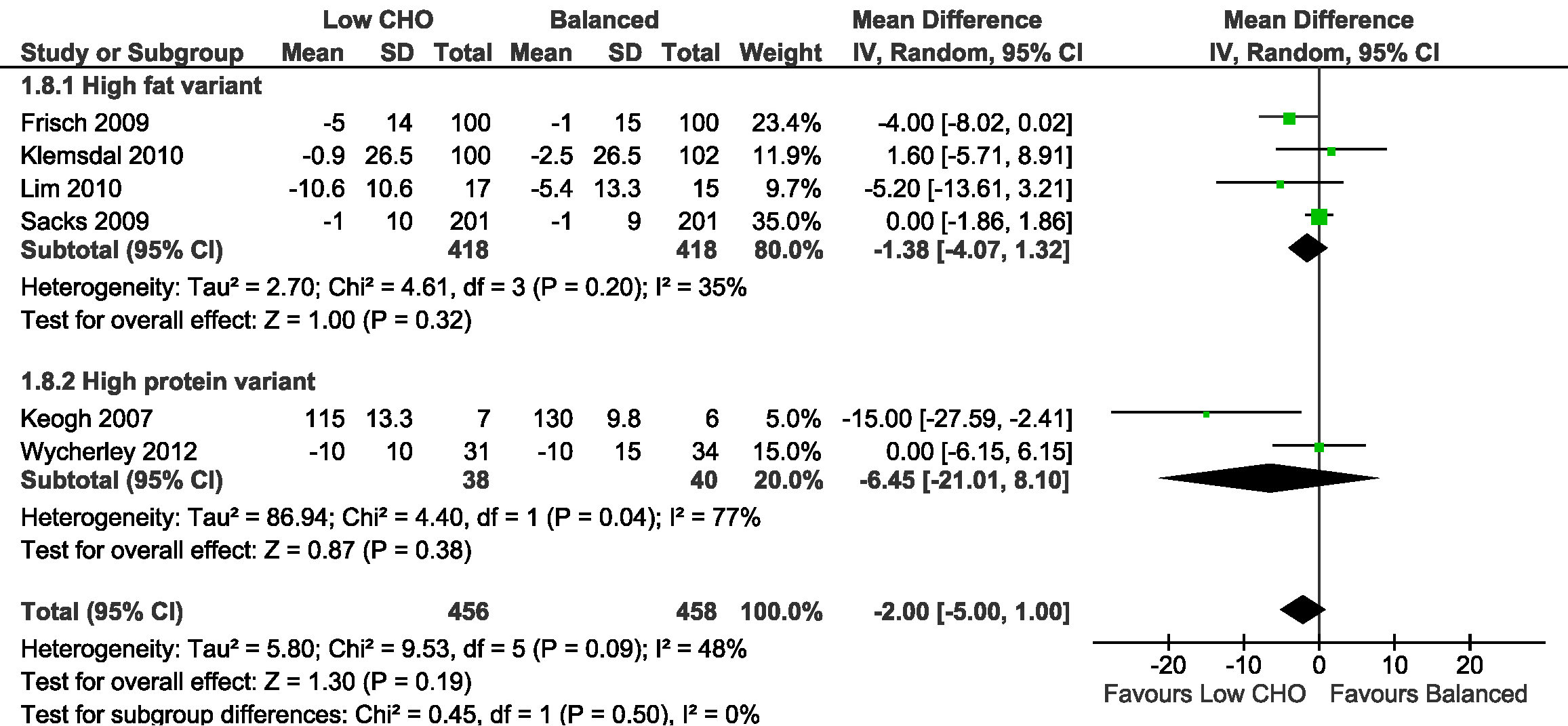


Figure S2F: Forest plot of low carbohydrate versus balanced diets in overweight and obese adults for systolic blood pressure (mmHg) at one to two years


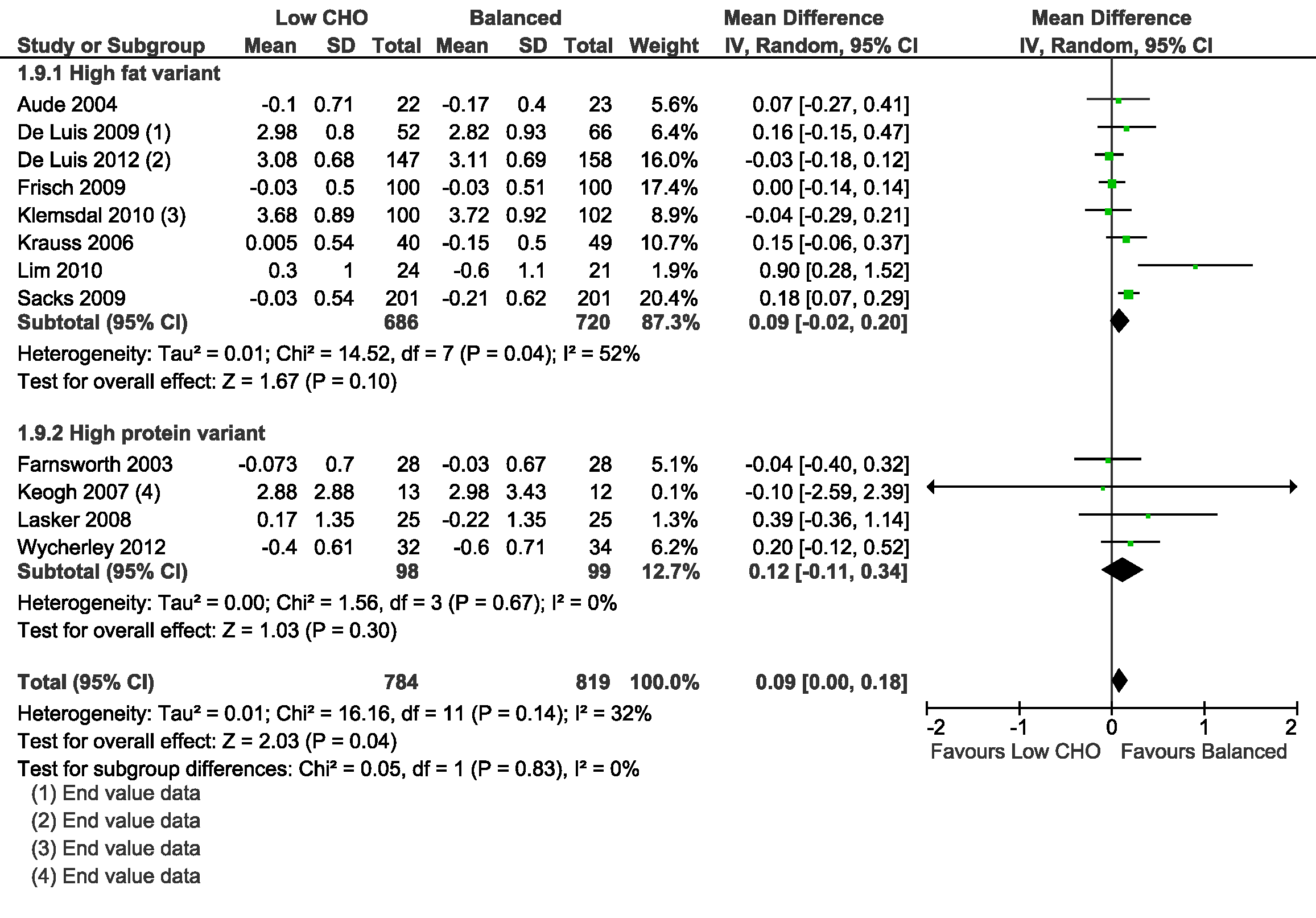
Figure S2G: Forest plot of low carbohydrate versus balanced diets in overweight and obese adults for serum LDL cholesterol (mmol/L) at three to six months


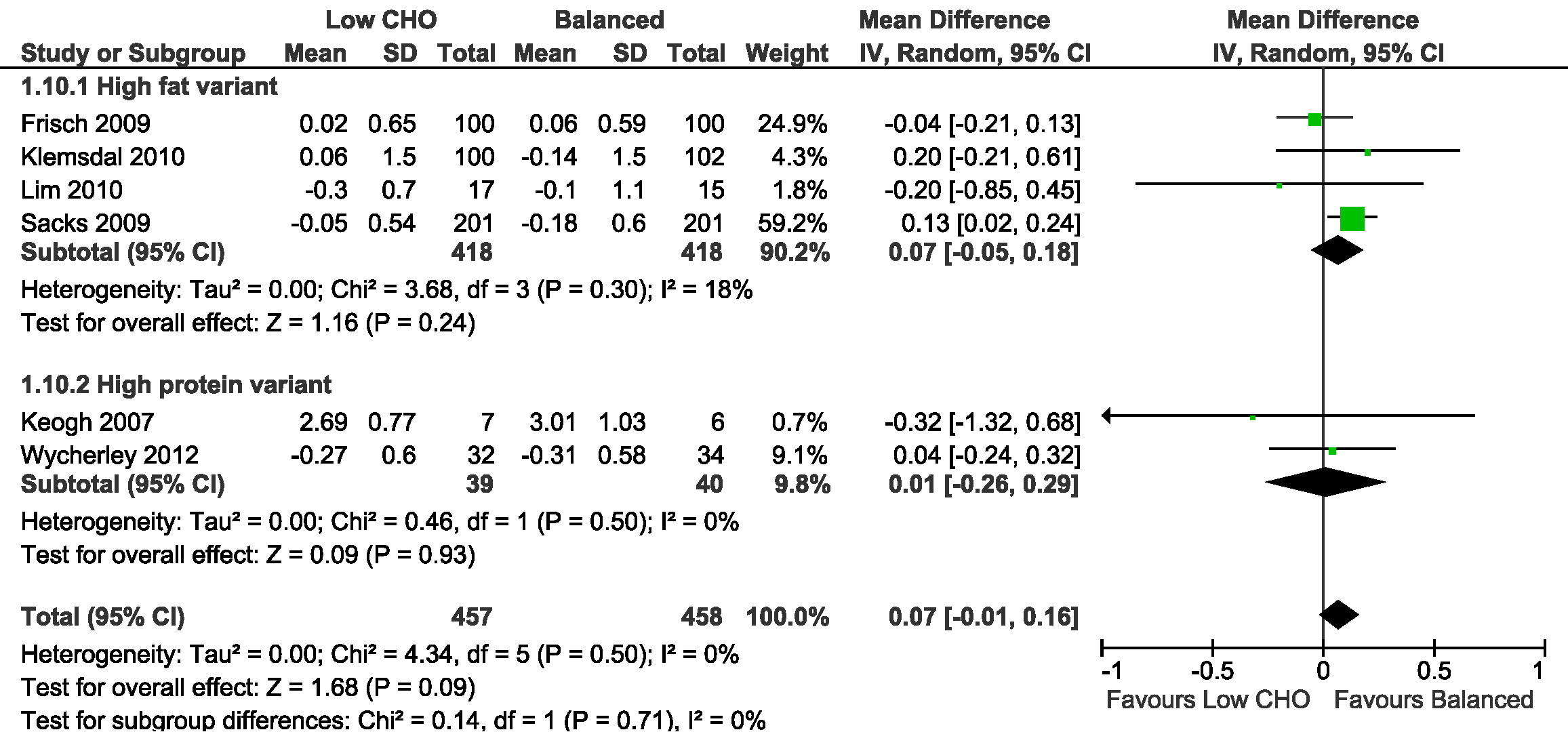


Figure S2H: Forest plot of low carbohydrate versus balanced diets in overweight and obese adults for serum LDL cholesterol (mmol/L) at one to two years


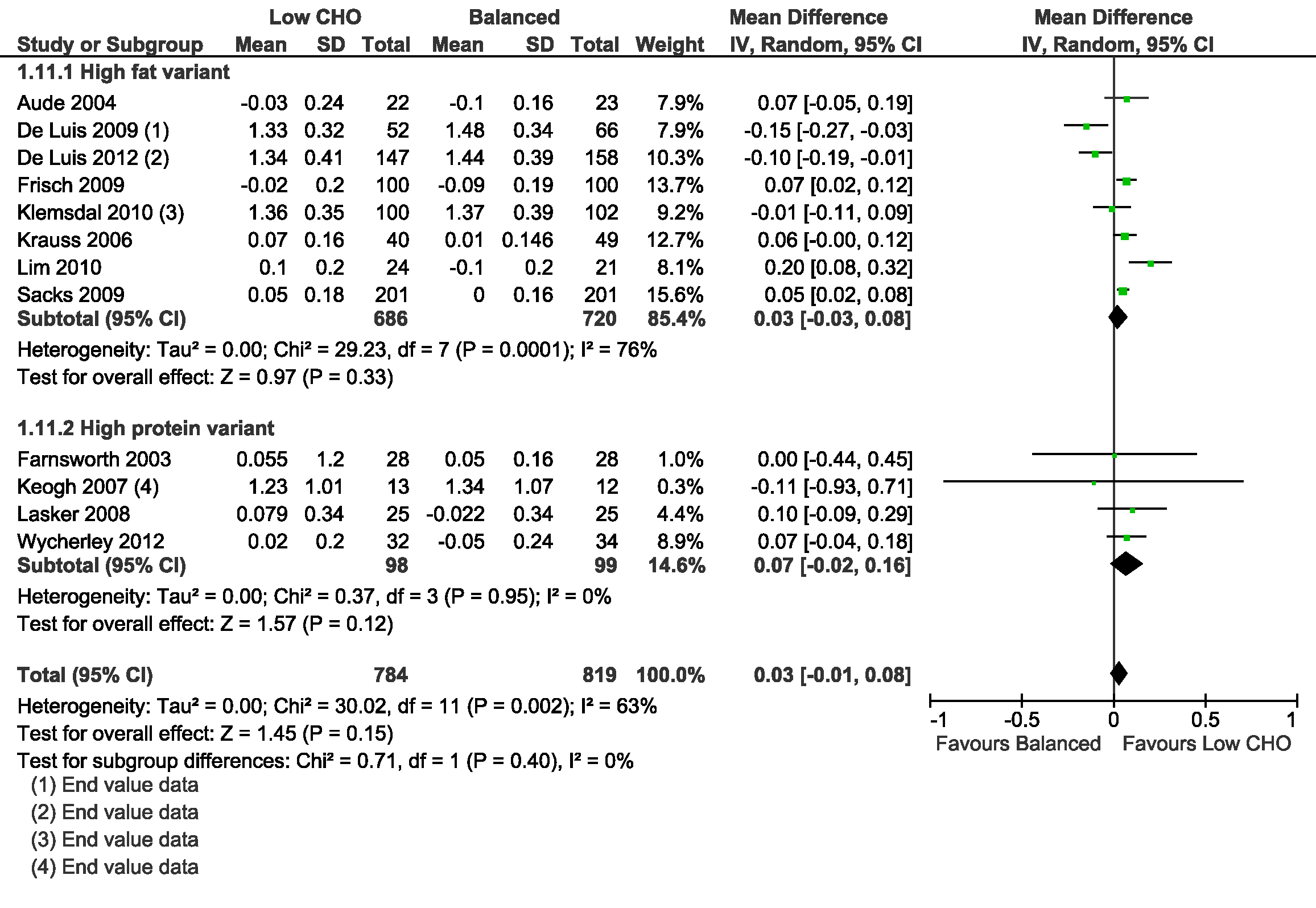
Figure S2I: Forest plot of low carbohydrate versus balanced diets in overweight and obese adults for serum HDL cholesterol (mmol/L) at three to six months


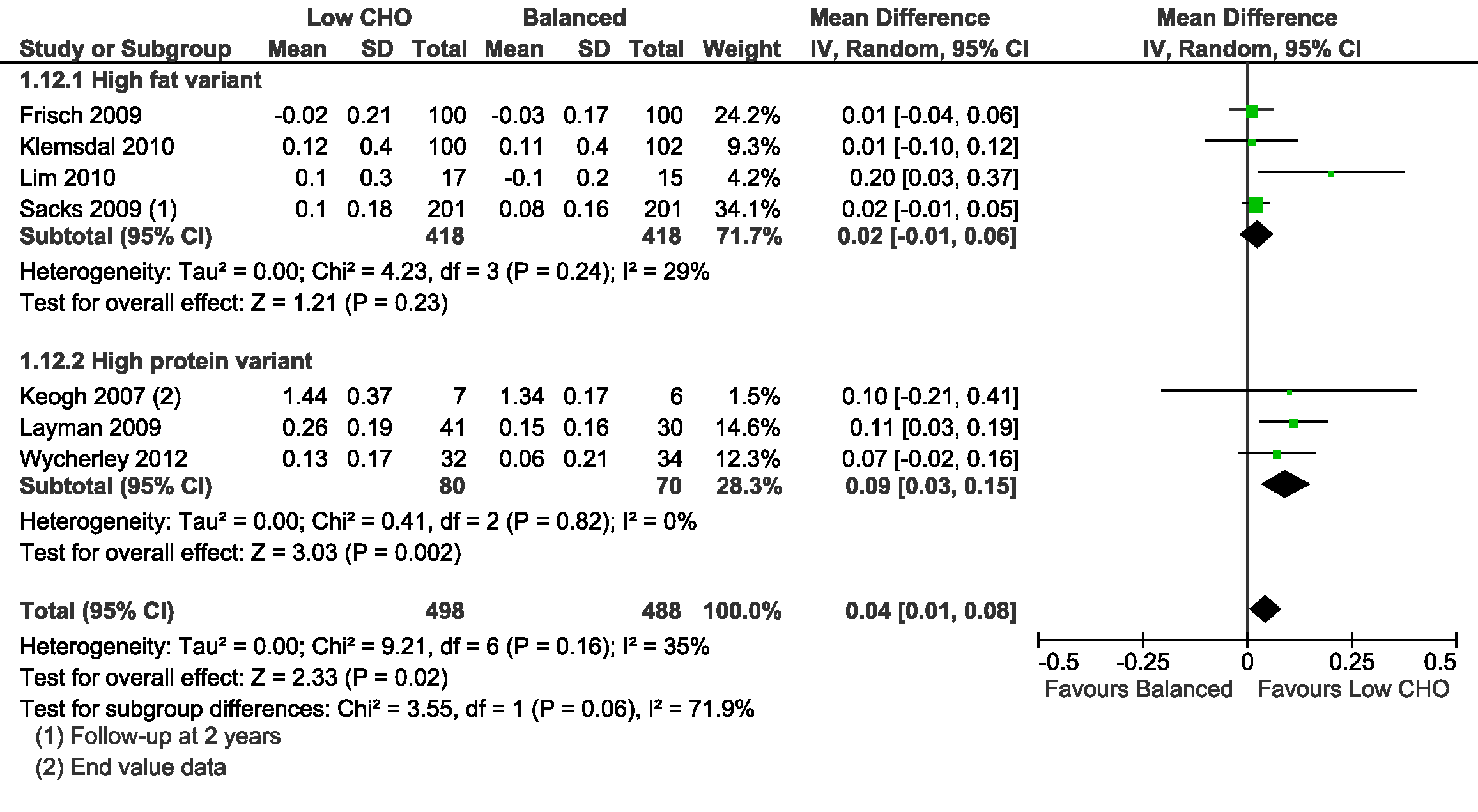
Figure S2J: Forest plot of low carbohydrate versus balanced diets in overweight and obese adults for HDL cholesterol (mmol/L) at one to two years


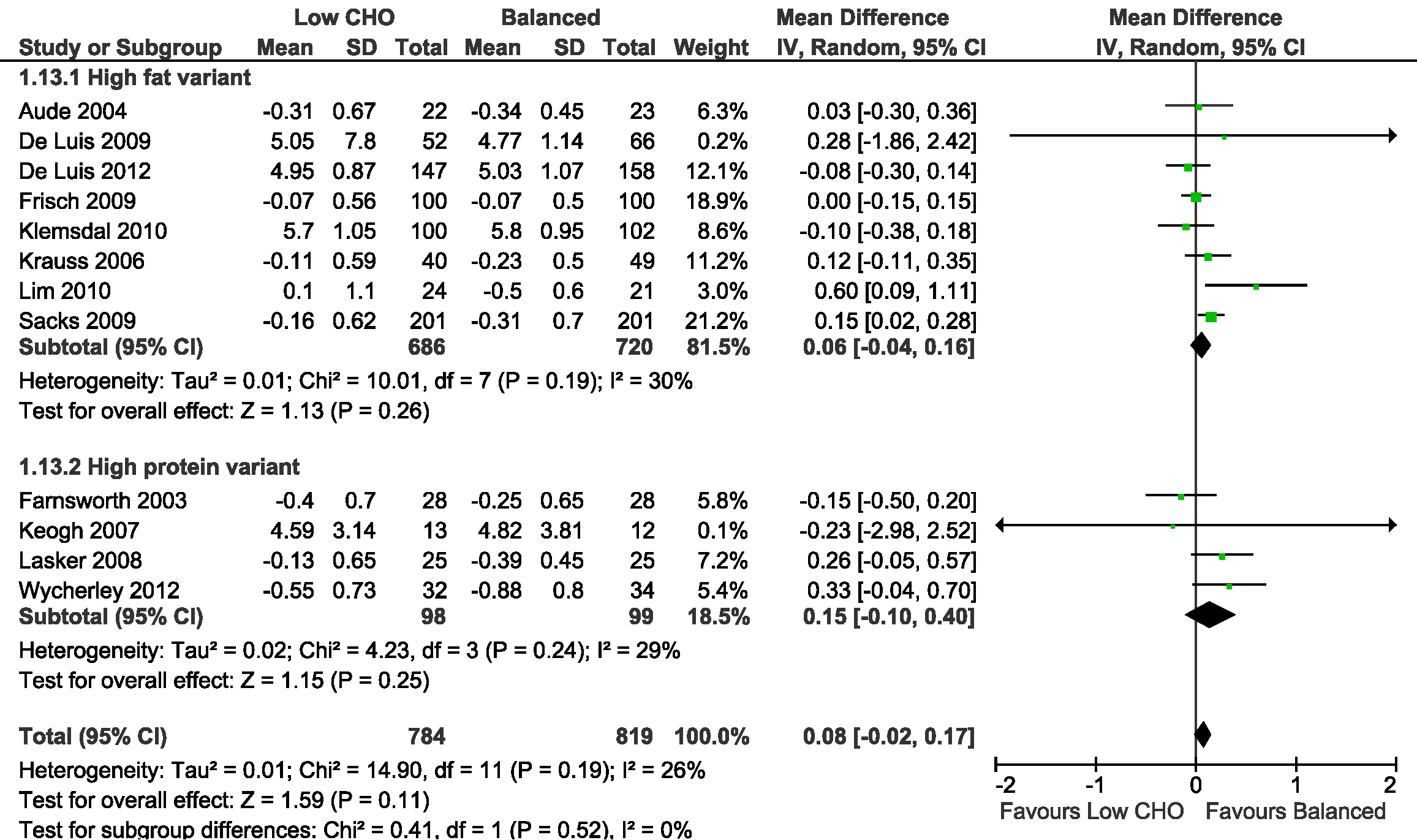


Figure S2K: Forest plot of low carbohydrate versus balanced diets in overweight and obese adults for serum total cholesterol (mmol/L) at three to six months


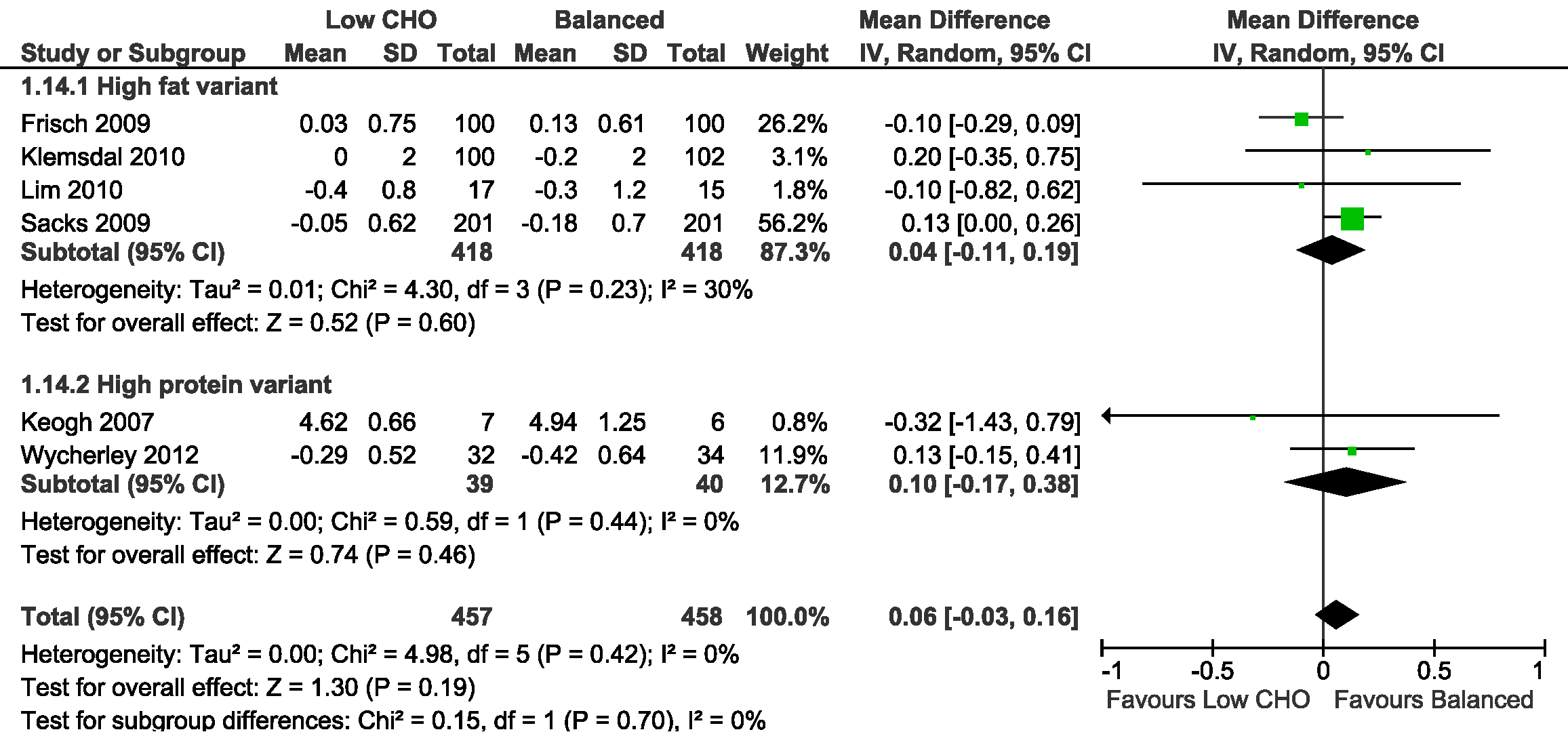


Figure S2L: Forest plot of low carbohydrate versus balanced diets in overweight and obese adults for serum total cholesterol (mmol/L) at one to two years


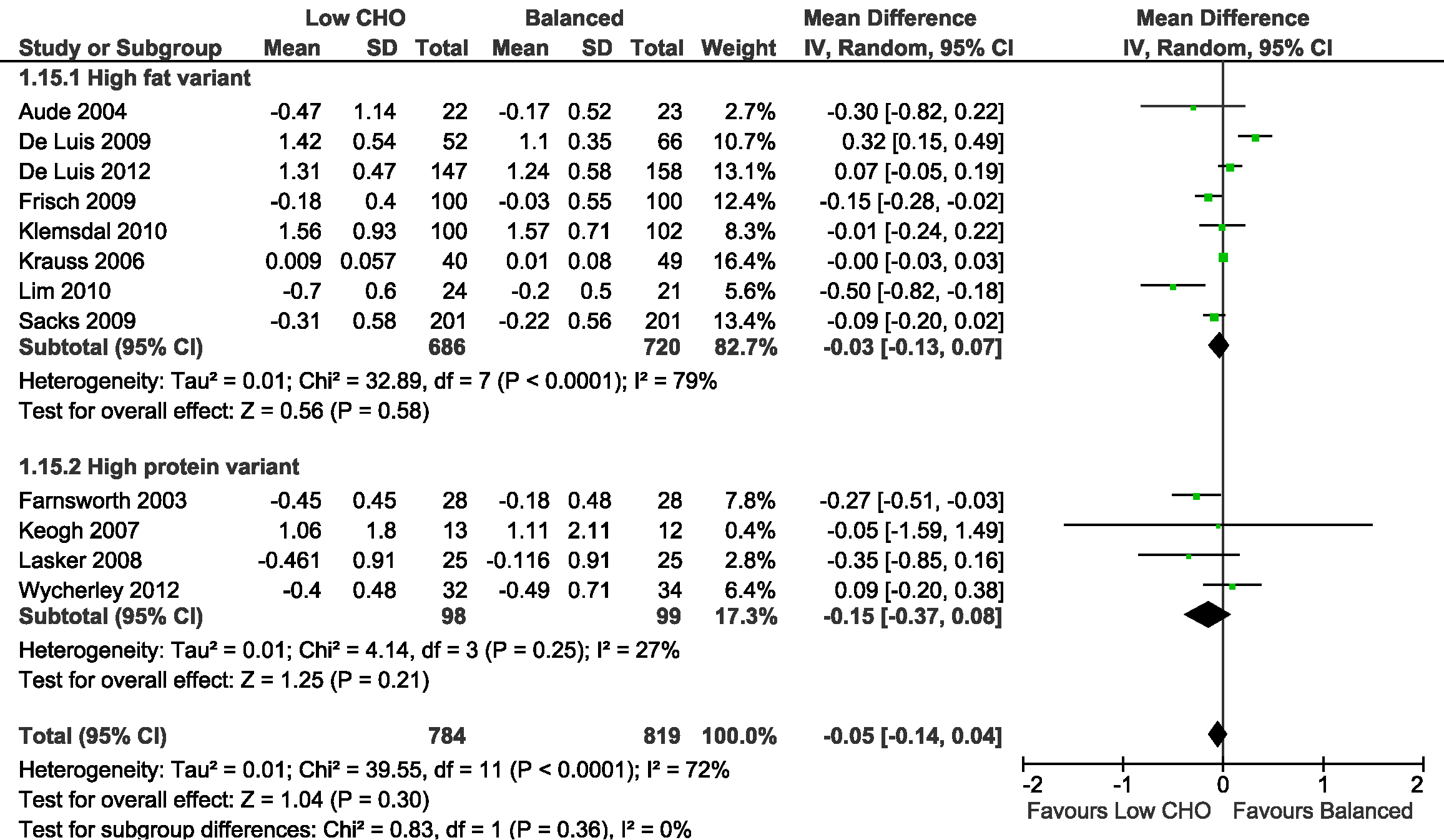


Figure S2M: Forest plot of low carbohydrate versus balanced diets in overweight and obese adults for serum triglycerides (mmol/L) at three to six months


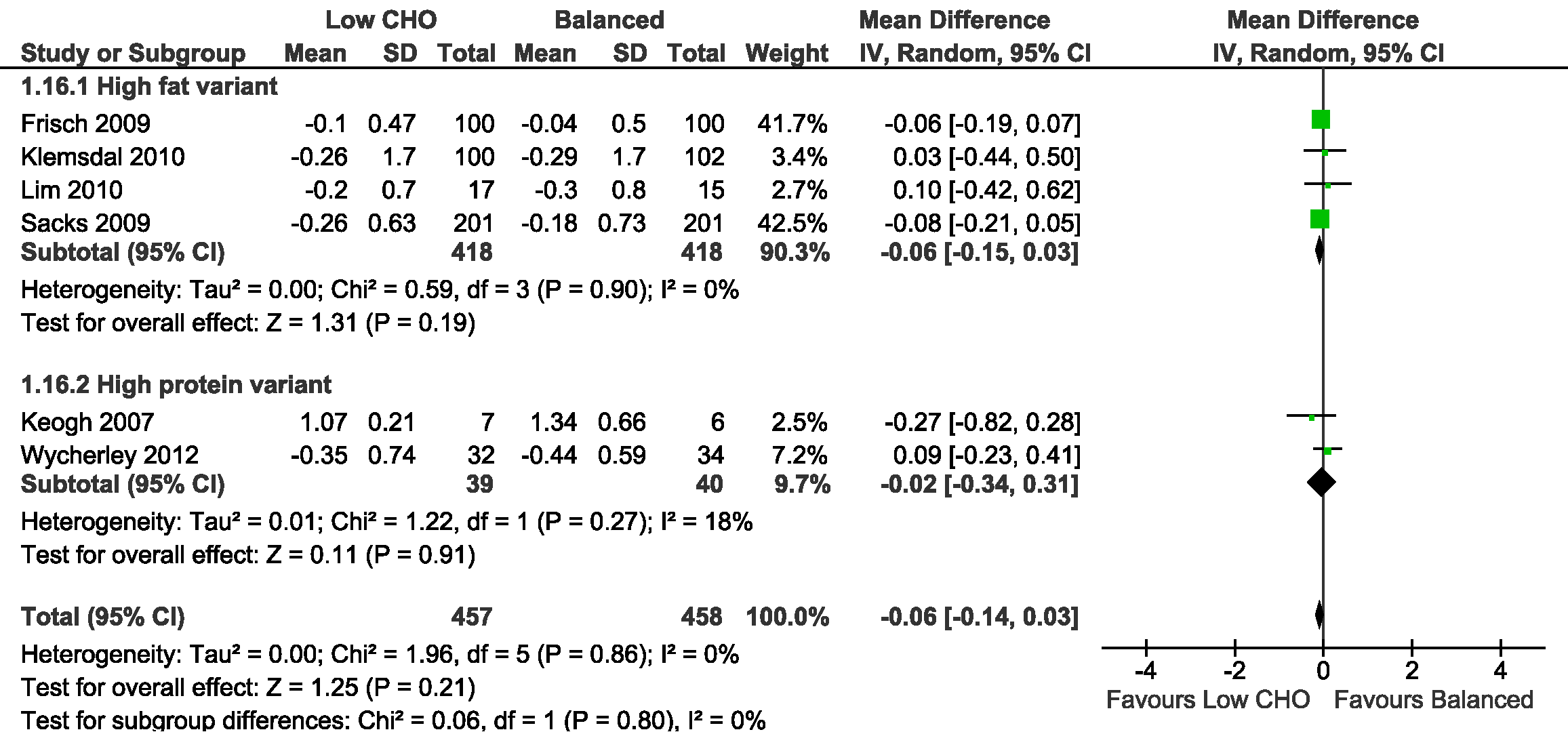


Figure S2N: Forest plot of low carbohydrate versus balanced diets in overweight and obese adults for serum triglycerides (mmol/L) at one to two years


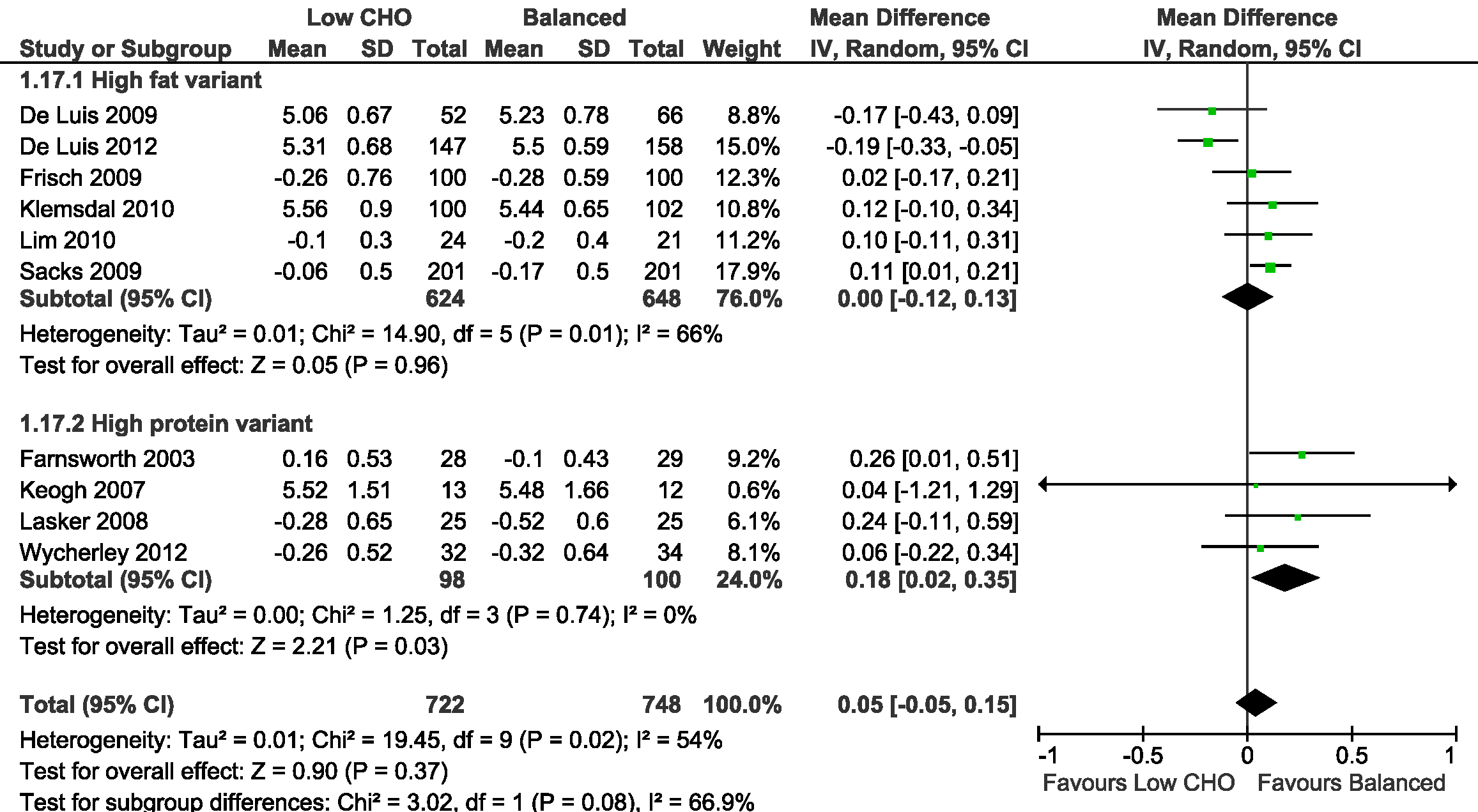


Figure S2O: Forest plot of low carbohydrate versus balanced diets in overweight and obese adults for fasting blood glucose (mmol/L) at three to six months


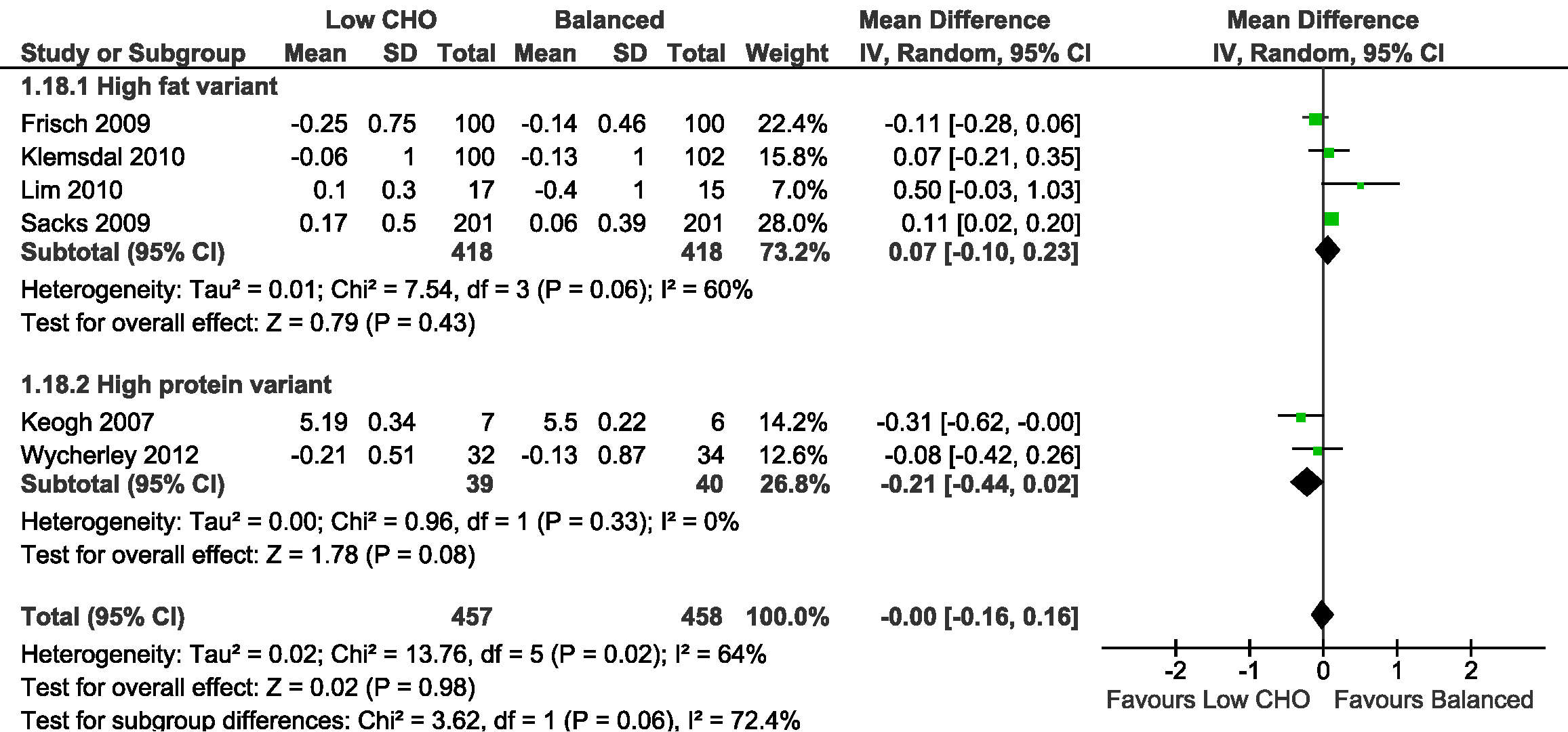


Figure S2P: Forest plot of low carbohydrate versus balanced diets in overweight and obese adults for fasting blood glucose (mmol/L) at one to two years
