# Supplementary material for: Low Carbohydrate versus Isoenergetic Balanced Diets for Reducing Weight and Cardiovascular Risk: A Systematic Review and Meta-Analysis
Source: PLoS One. 2014 Jul 9;9(7):e100652. doi: 10.1371/journal.pone.0100652 (PMC4090010; doi:10.1371/journal.pone.0100652)
Supplement: Support Information S3 — Forest plots of meta-analyses in overweight and obese adults with type 2 diabetes mellitus. Figure S3A: Forest plot of low carbohydrate versus balanced diets in overweight and obese adults with type 2 diabetes mellitus for body mass index (kg/m2) at three to six months. Figure S3B: Forest plot of low carbohydrate versus balanced diets in overweight and obese adults with type 2 diabetes mellitus of body mass index (kg/m2) at two years. Figure S2C: Forest plot of low carbohydrate versus balanced diets in overweight and obese adults with type 2 diabetes mellitus for glycosylated hemoglobin (%) at three to six months. Figure S3D: Forest plot of low carbohydrate versus balanced diets in overweight and obese adults with type 2 diabetes mellitus for glycolosated hemoglobin (%) at one to two years. Figure S3E: Forest plot of low carbohydrate versus balanced diets in overweight and obese adults with type 2 diabetes mellitus for fasting blood glucose (mmol/L) at three to six months. Figure S3F: Forest plot of low carbohydrate versus balanced diets in overweight and obese adults with type 2 diabetes mellitus for fasting blood glucose (mmol/L) at 15 months. Figure S3G: Forest plot of low carbohydrate versus balanced diets in overweight and obese adults with type 2 diabetes mellitus for diastolic blood pressure (mmHg) at three to six months. Figure S3H: Forest plot of low carbohydrate versus balanced diets in overweight and obese adults with type 2 diabetes mellitus for diastolic blood pressure (mmHg) at one to two years. Figure S3I: Forest plot of low carbohydrate versus balanced diets in overweight and obese adults with type 2 diabetes mellitus for systolic blood pressure (mmHg) at three to six months. Figure S3J: Forest plot of low carbohydrate versus balanced diets in overweight and obese adults with type 2 diabetes mellitus for systolic blood pressure (mmHg) at one to two years. Figure S3K: Forest plot of low carbohydrate versus balanced diets in overweight and obese adults [file pone.0100652.s004.docx]

**Low carbohydrate versus isoenergetic balanced diets for reducing weight and cardiovascular risk:**

**a systematic review and meta-analysis**

**Support Information S3: Forest plots of meta-analyses in overweight and obese adults with type 2 diabetes mellitus**


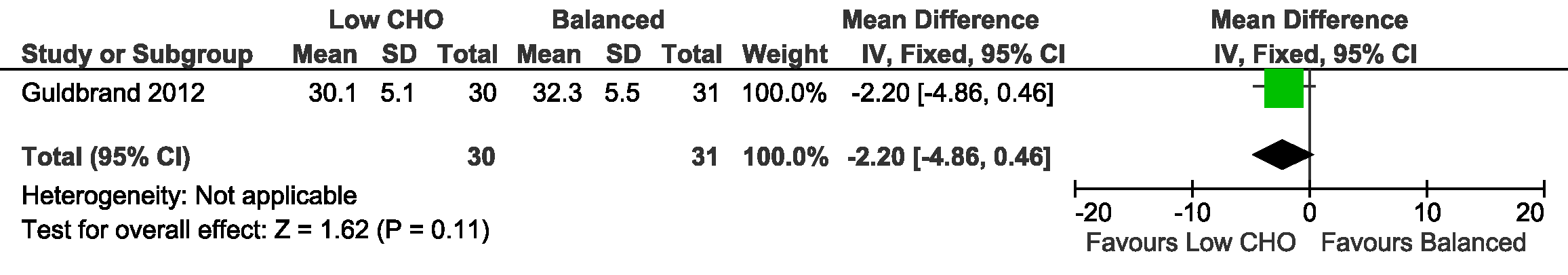


Figure S3A: Forest plot of low carbohydrate versus balanced diets in overweight and obese adults with type 2 diabetes mellitus for body mass index (kg/m^2^) at three to six months


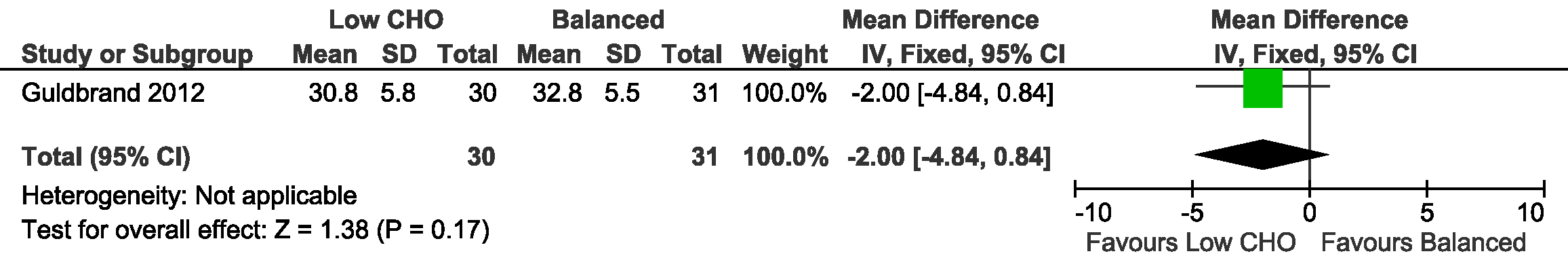


Figure S3B: Forest plot of low carbohydrate versus balanced diets in overweight and obese adults with type 2 diabetes mellitus of body mass index (kg/m^2^) at two years


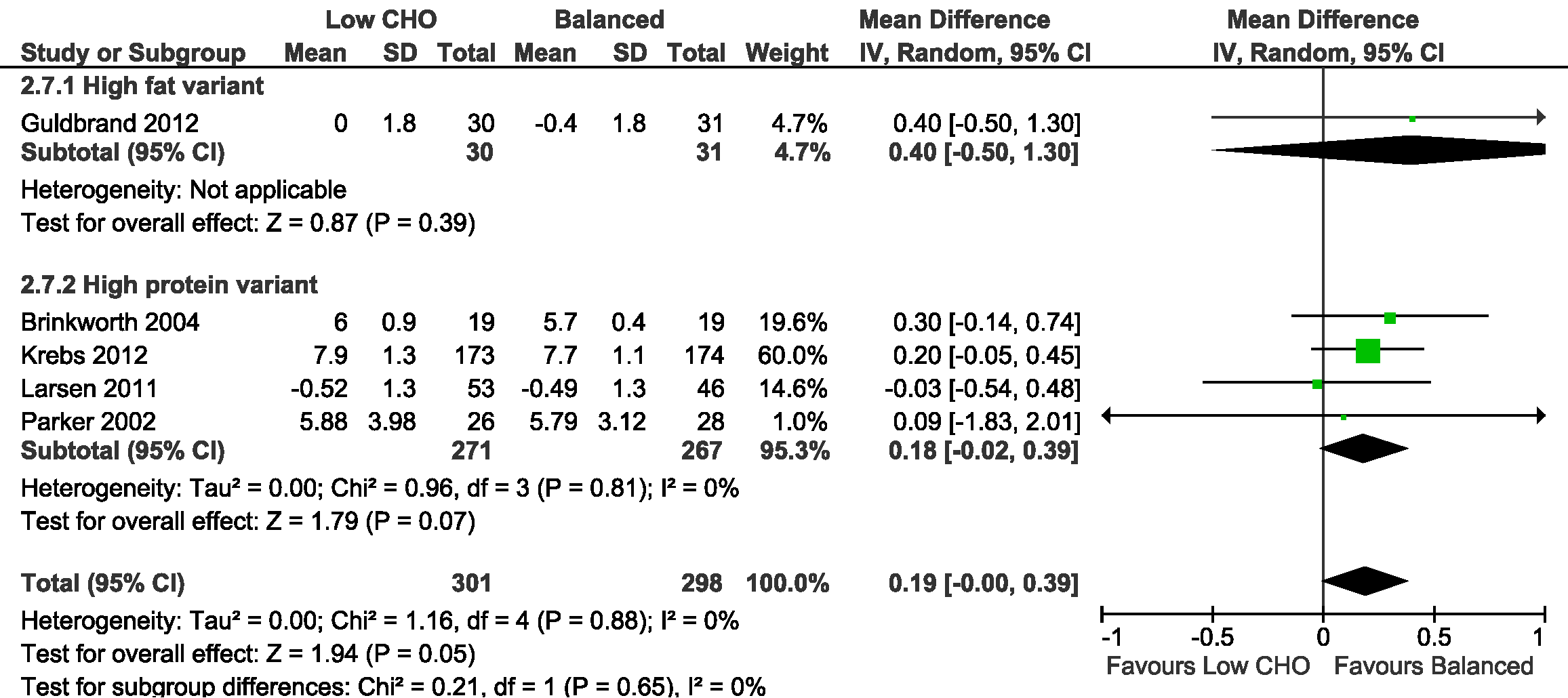


Figure S2C: Forest plot of low carbohydrate versus balanced diets in overweight and obese adults with type 2 diabetes mellitus for glycosylated hemoglobin (%) at three to six months


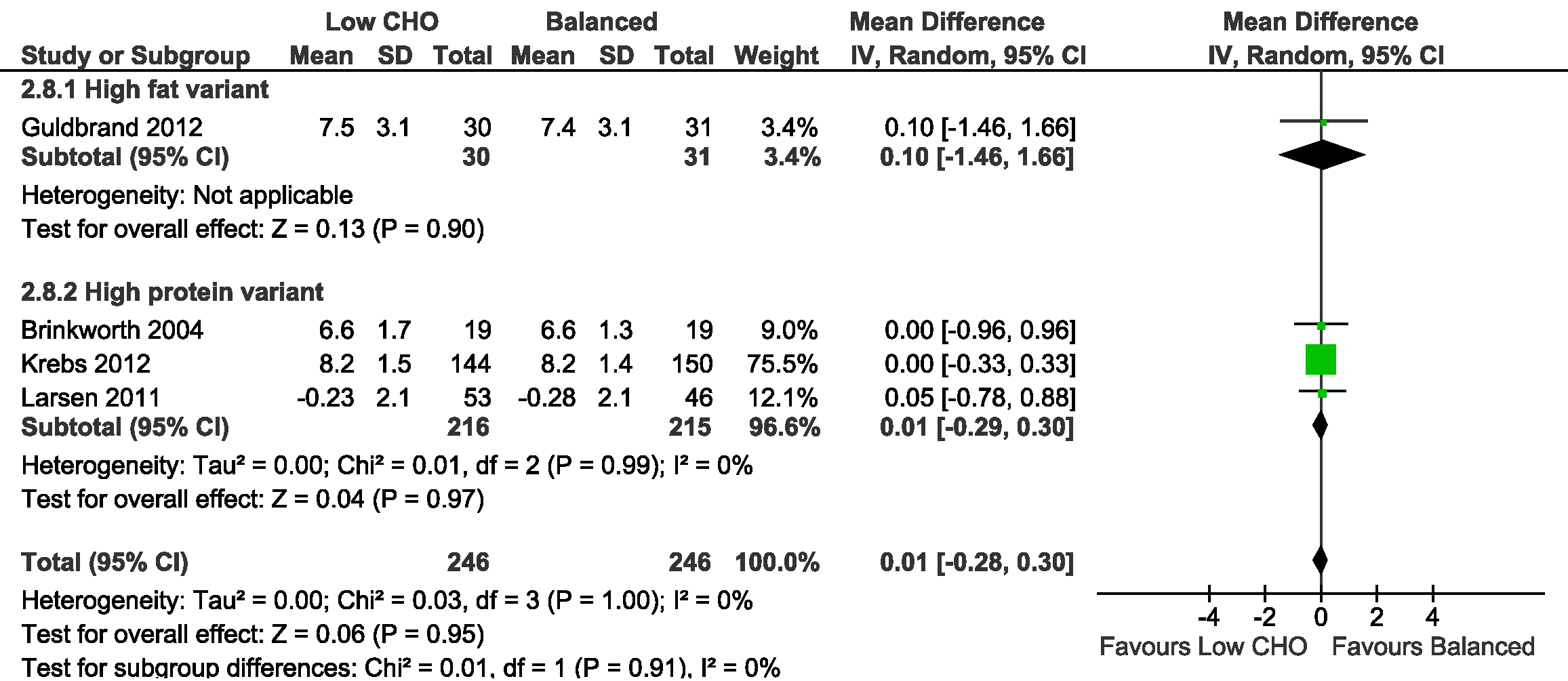


Figure S3D: Forest plot of low carbohydrate versus balanced diets in overweight and obese adults with type 2 diabetes mellitus for glycolosated hemoglobin (%) at one to two years


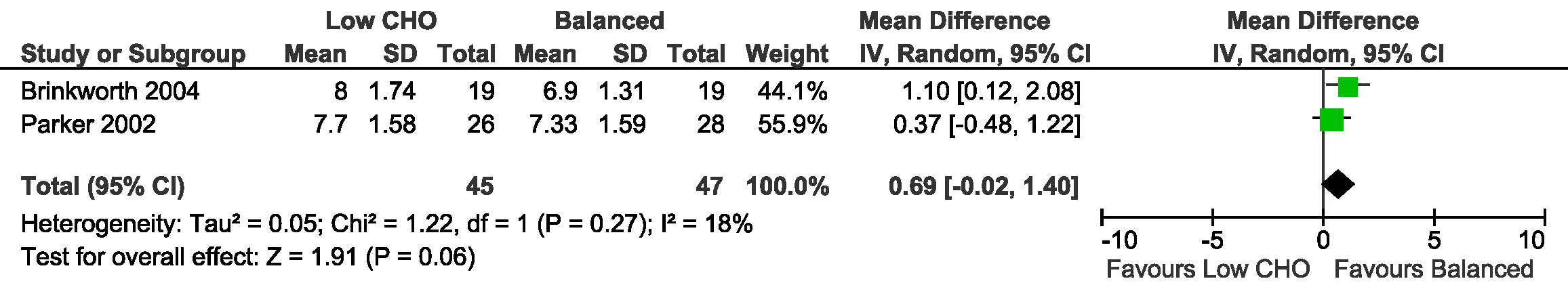


Figure S3E: Forest plot of low carbohydrate versus balanced diets in overweight and obese adults with type 2 diabetes mellitus for fasting blood glucose (mmol/L) at three to six months


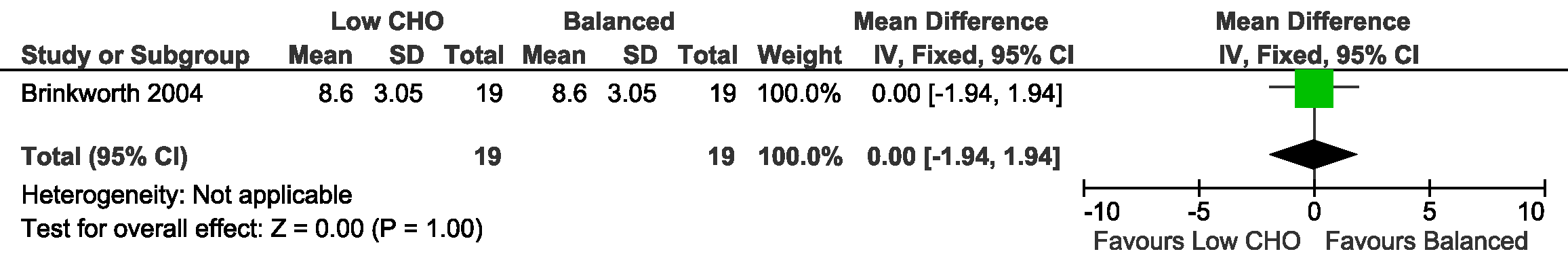


Figure S3F: Forest plot of low carbohydrate versus balanced diets in overweight and obese adults with type 2 diabetes mellitus for fasting blood glucose (mmol/L) at 15 months


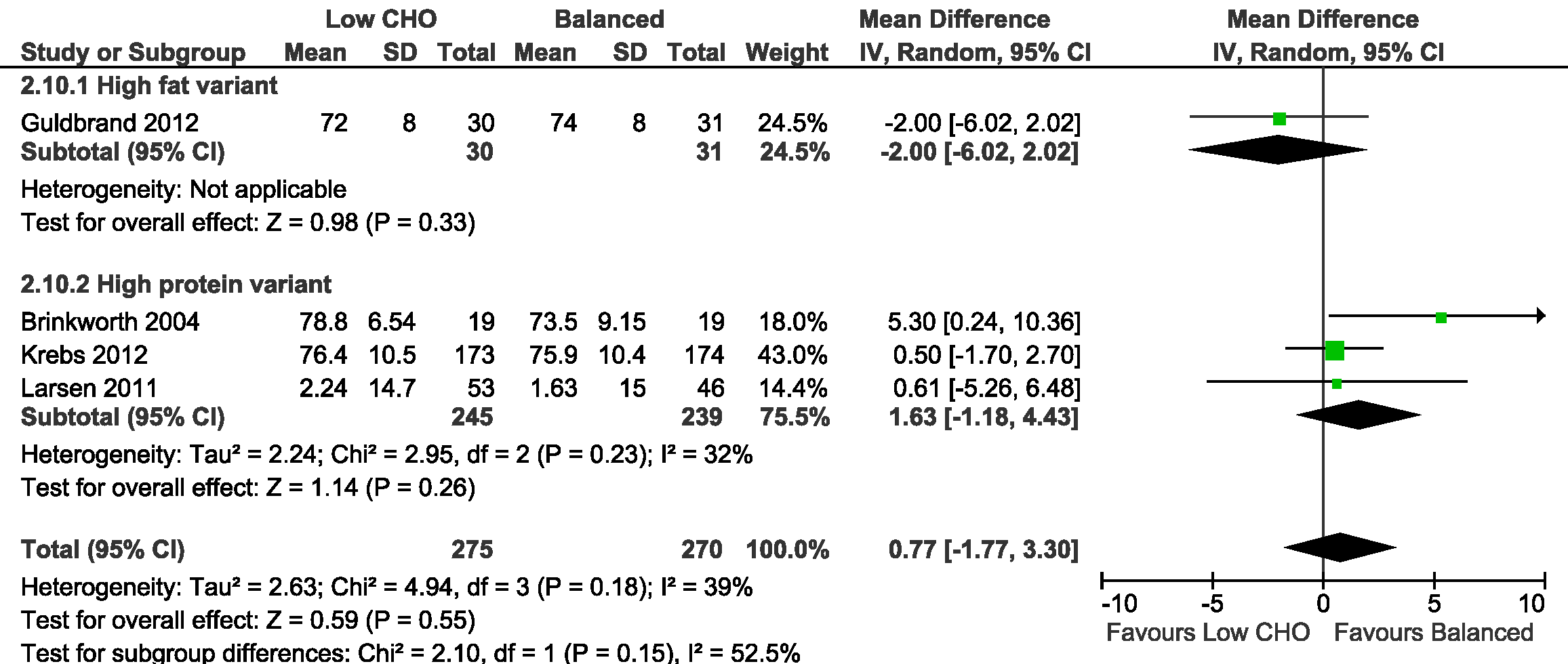


Figure S3G: Forest plot of low carbohydrate versus balanced diets in overweight and obese adults with type 2 diabetes mellitus for diastolic blood pressure (mmHg) at three to six months


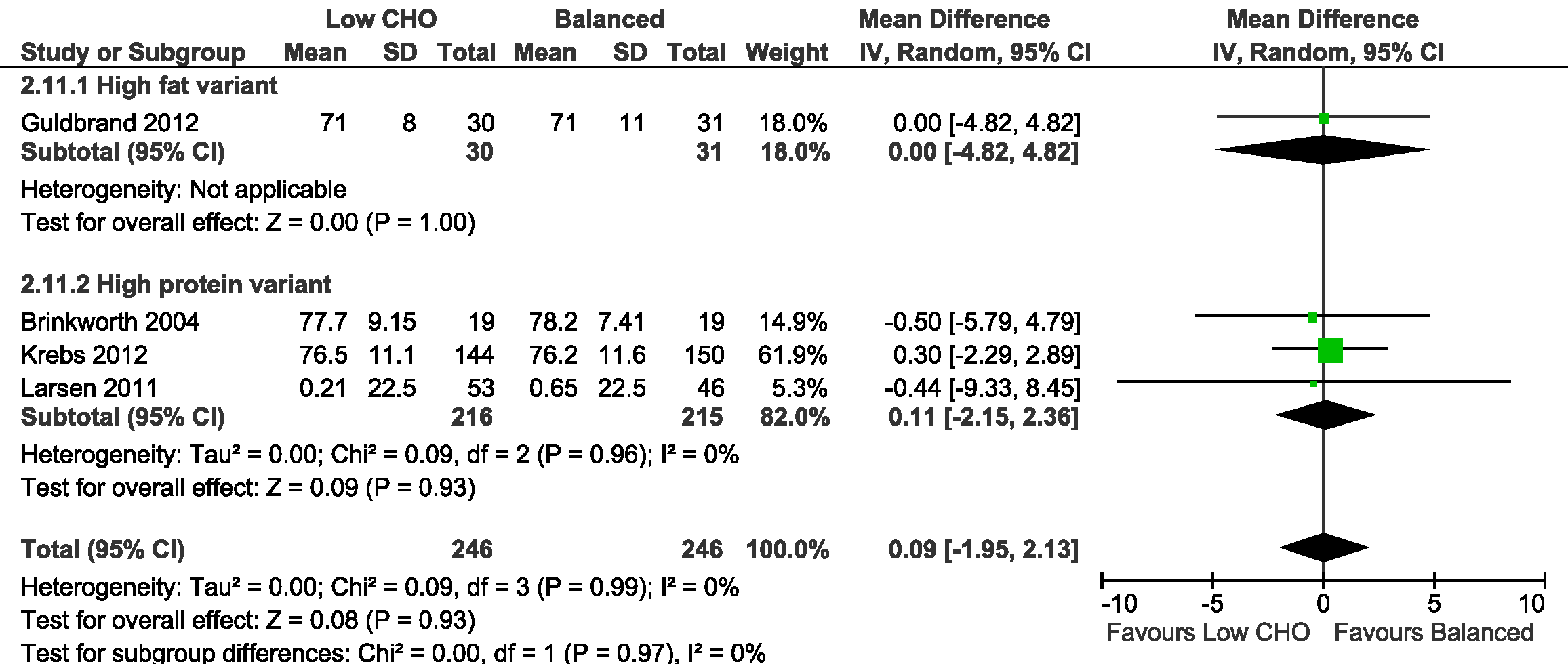


Figure S3H: Forest plot of low carbohydrate versus balanced diets in overweight and obese adults with type 2 diabetes mellitus for diastolic blood pressure (mmHg) at one to two years


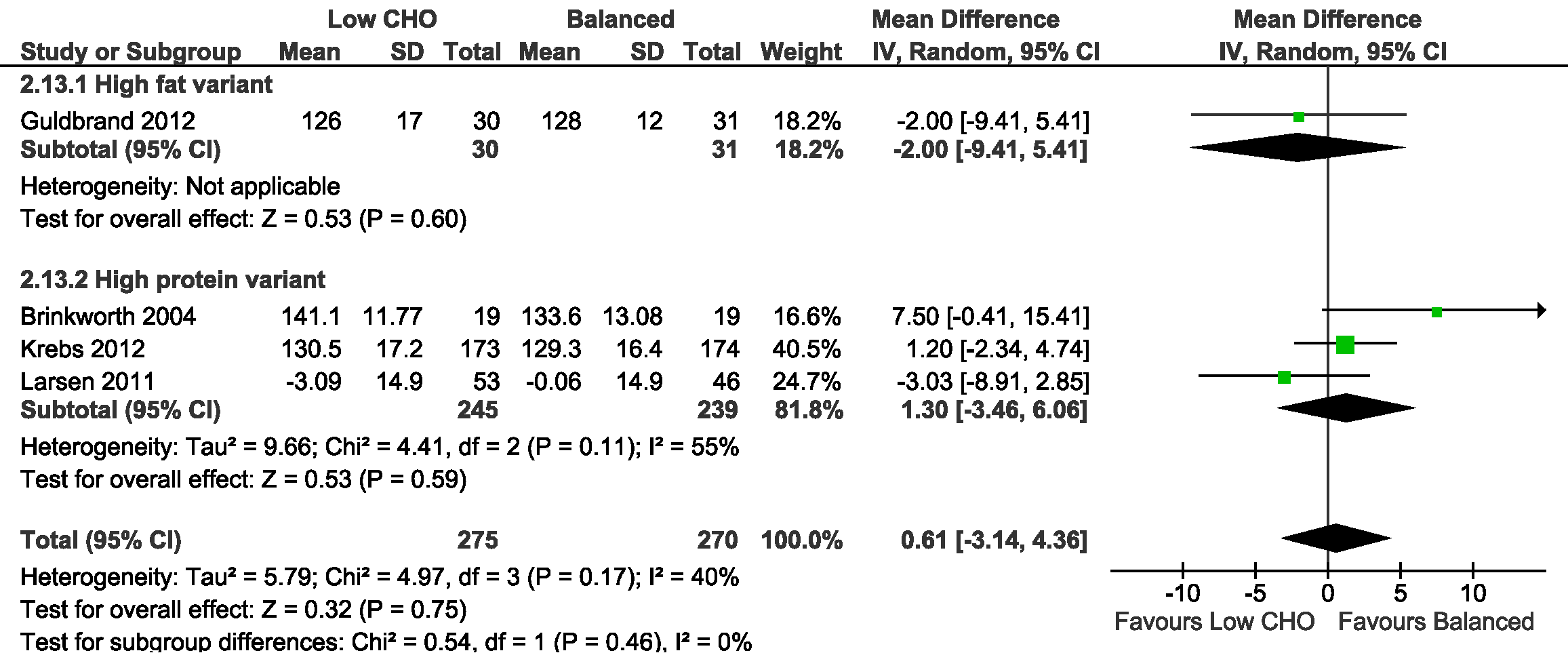


Figure S3I: Forest plot of low carbohydrate versus balanced diets in overweight and obese adults with type 2 diabetes mellitus for systolic blood pressure (mmHg) at three to six months


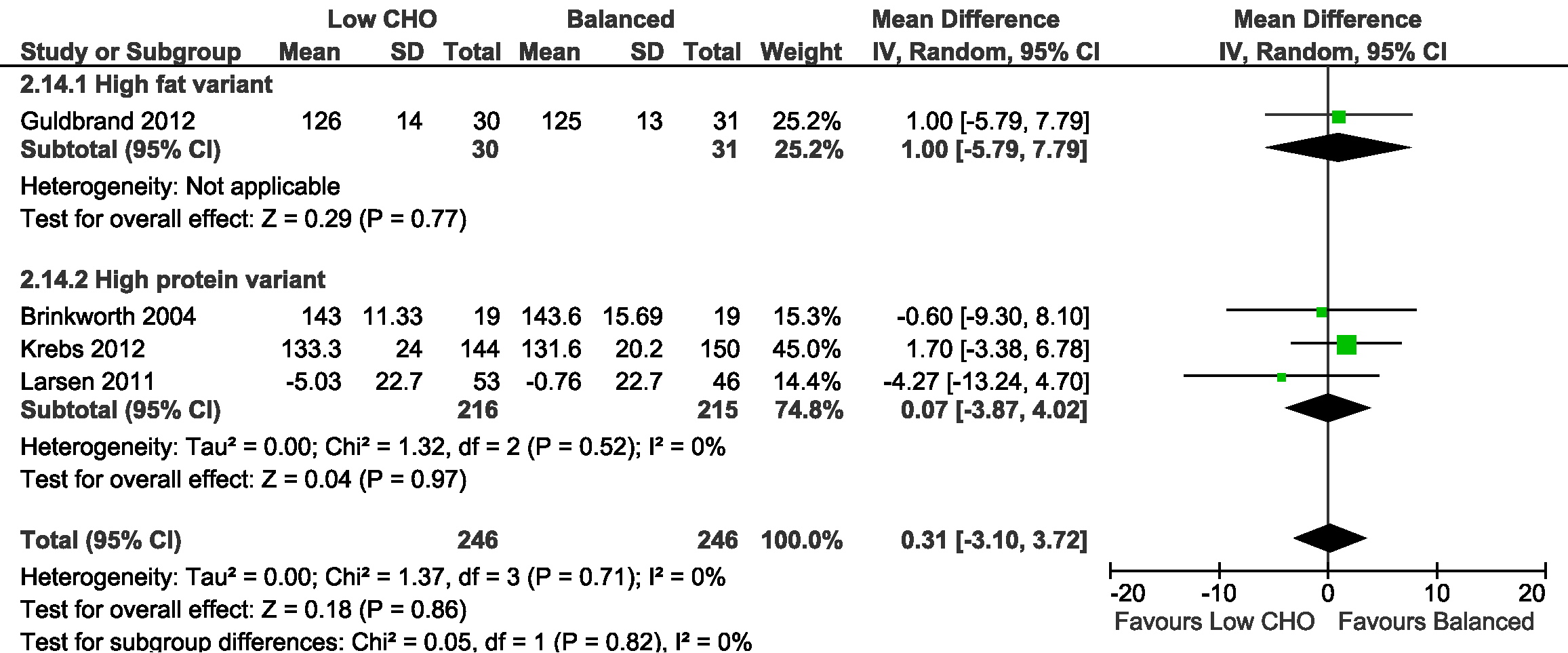


Figure S3J: Forest plot of low carbohydrate versus balanced diets in overweight and obese adults with type 2 diabetes mellitus for systolic blood pressure (mmHg) at one to two years


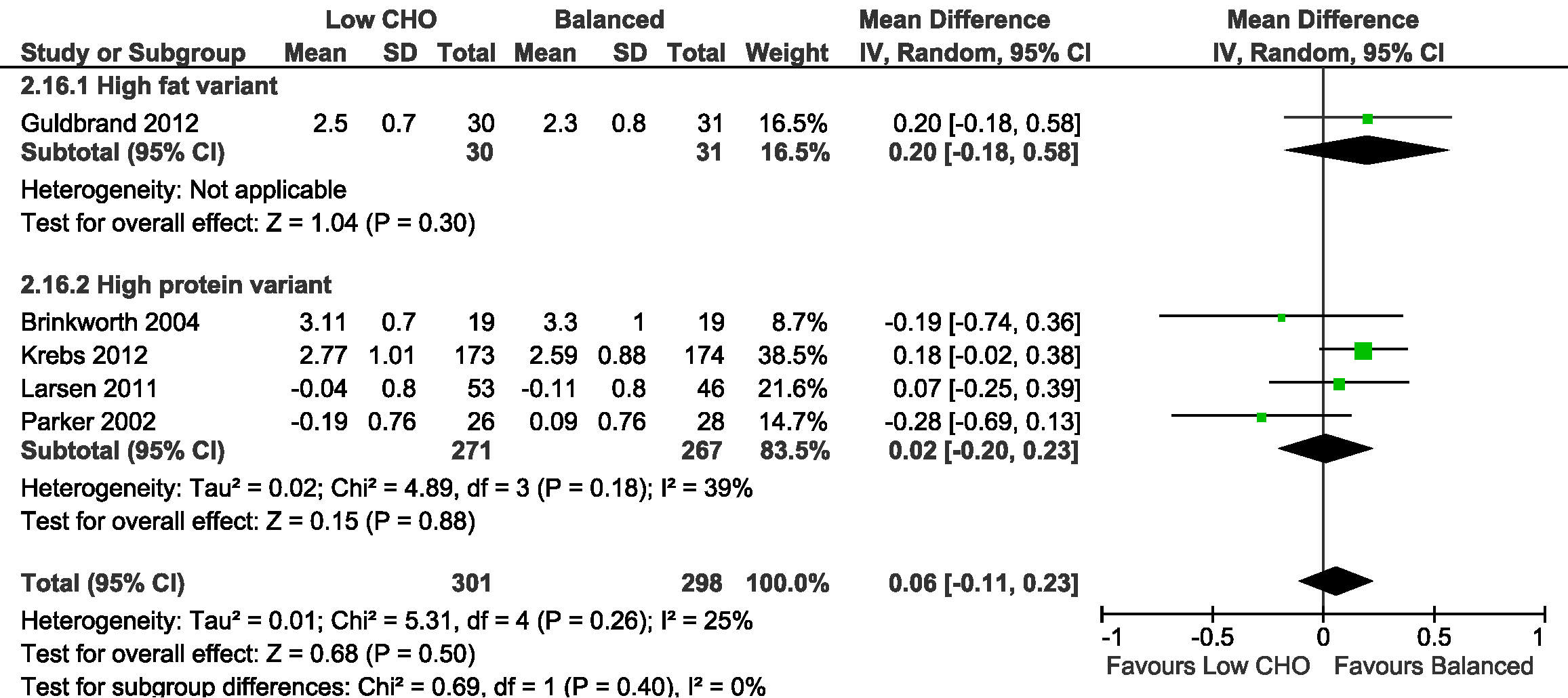


Figure S3K: Forest plot of low carbohydrate versus balanced diets in overweight and obese adults with type 2 diabetes mellitus for LDL cholesterol (mmol/L) at three to six months


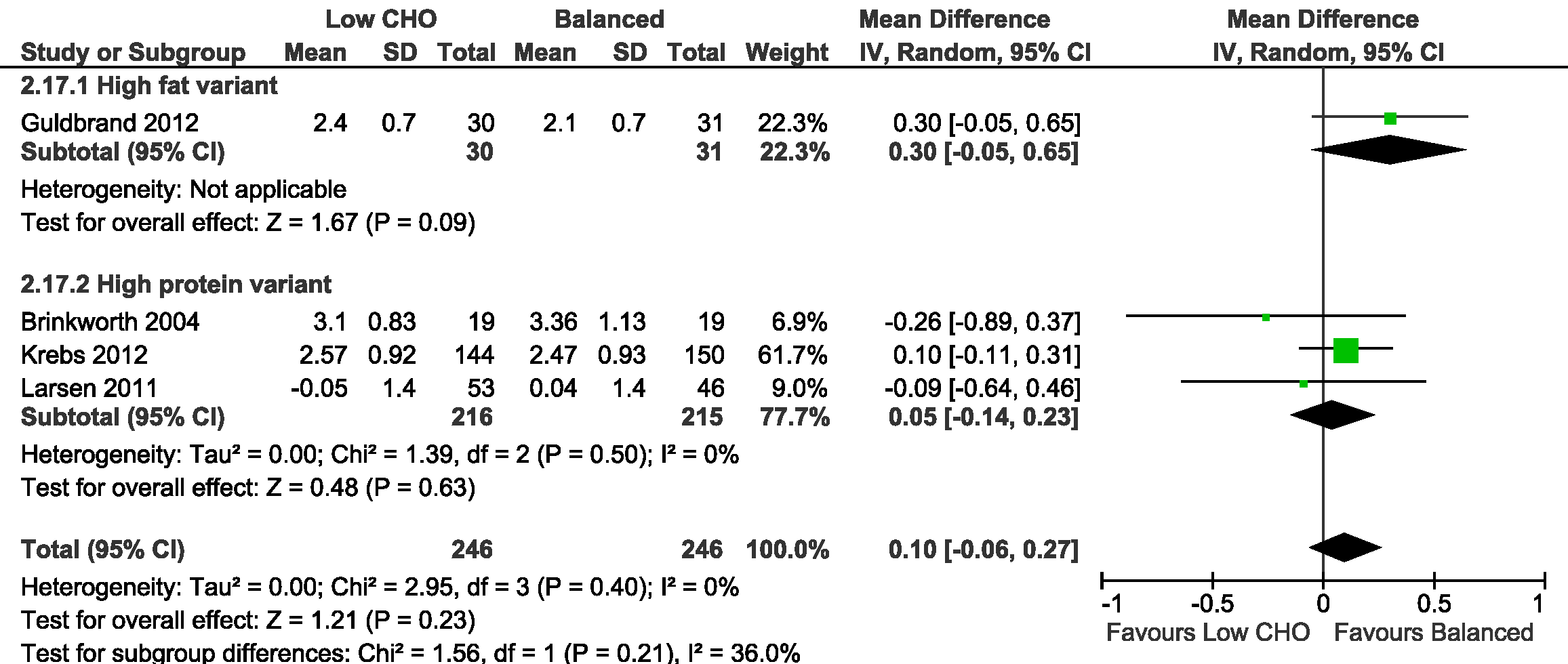


Figure S3L: Forest plot of low carbohydrate versus balanced diets in overweight and obese adults with type 2 diabetes mellitus for LDL cholesterol (mmol/L) at one to two years


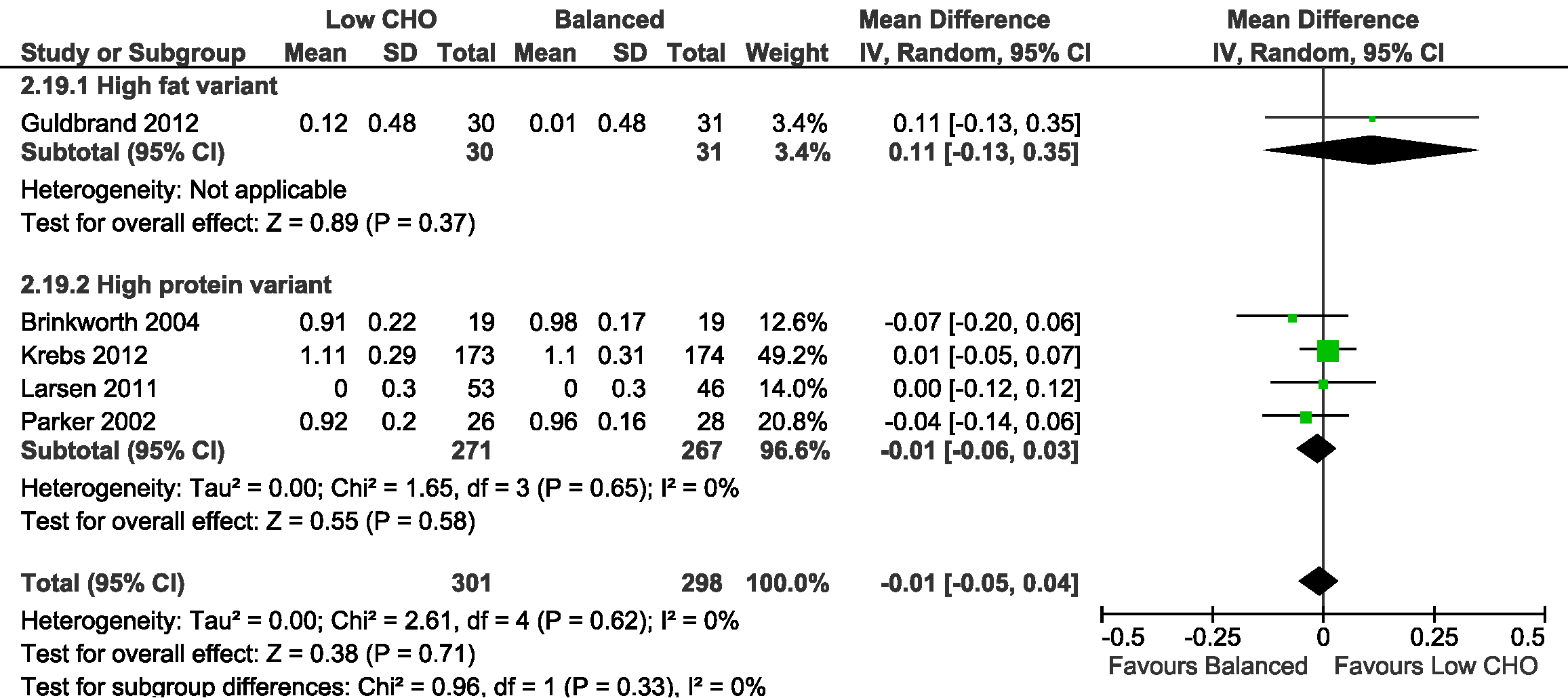


Figure S3M: Forest plot of low carbohydrate versus balanced diets in overweight and obese adults with type 2 diabetes mellitus for HDL cholesterol (mmol/L) at three to six months


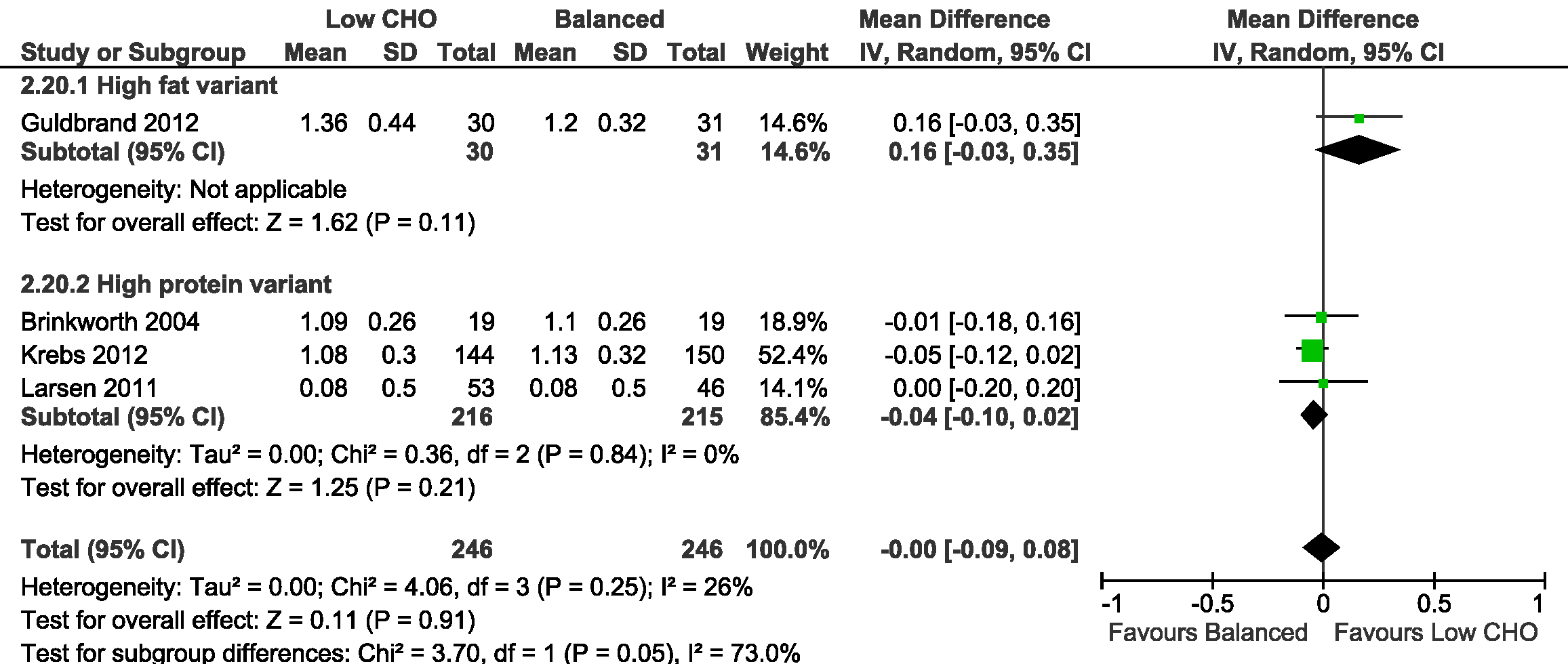


Figure S3N: Forest plot of low carbohydrate versus balanced diets in overweight and obese adults with type 2 diabetes mellitus for HDL cholesterol (mmol/L) at one to two years


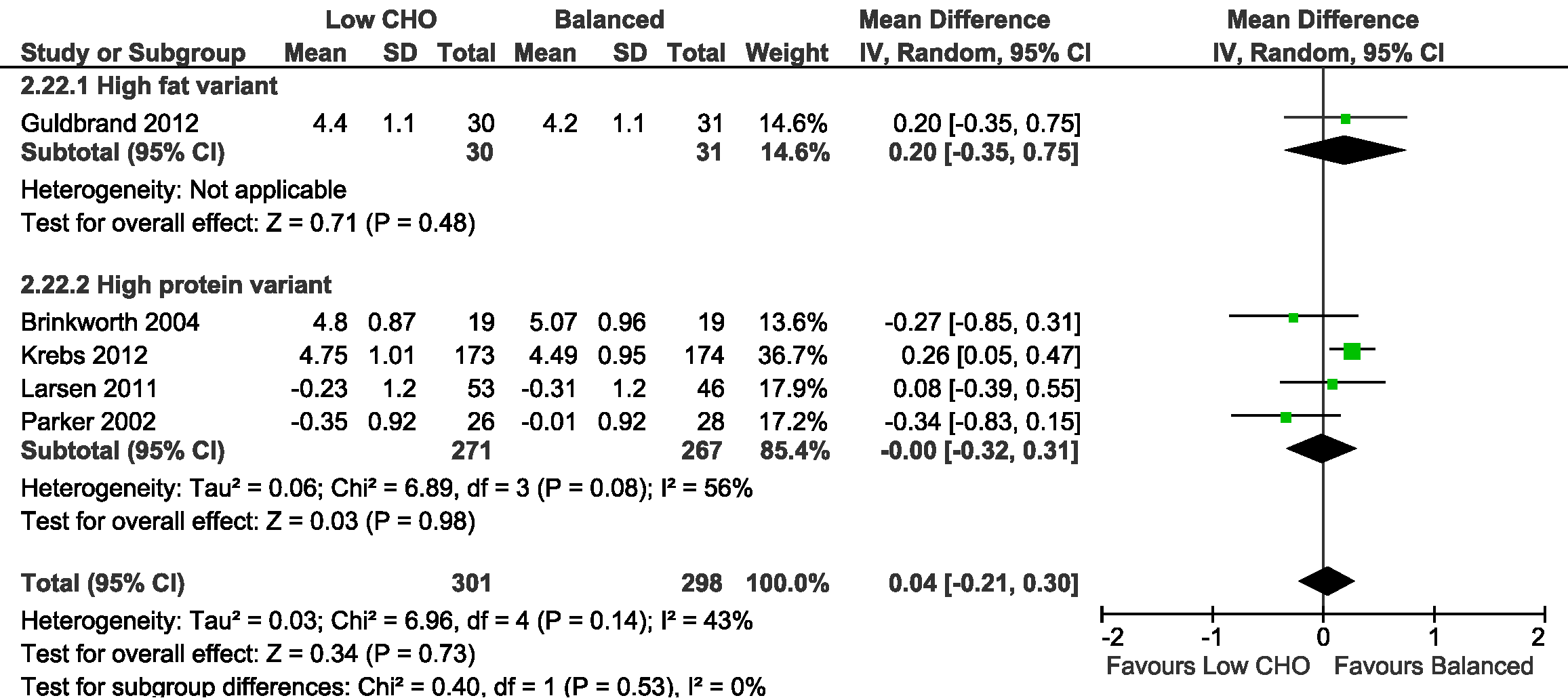


Figure S3O: Forest plot of low carbohydrate versus balanced diets in overweight and obese adults with type 2 diabetes mellitus of total cholesterol (mmol/L) at three to six months


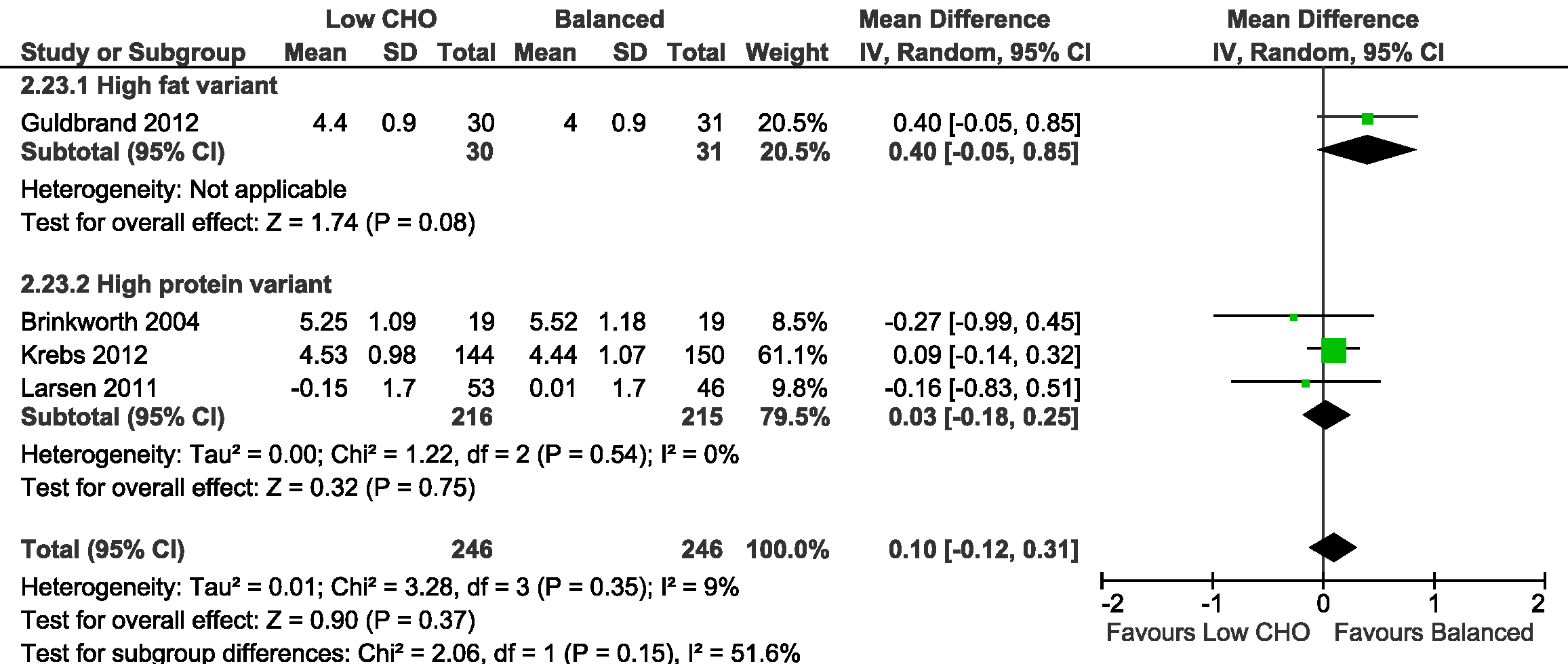


Figure S3P: Forest plot of low carbohydrate versus balanced diets in overweight and obese adults with type 2 diabetes mellitus for total cholesterol (mmol/L) at one to two years


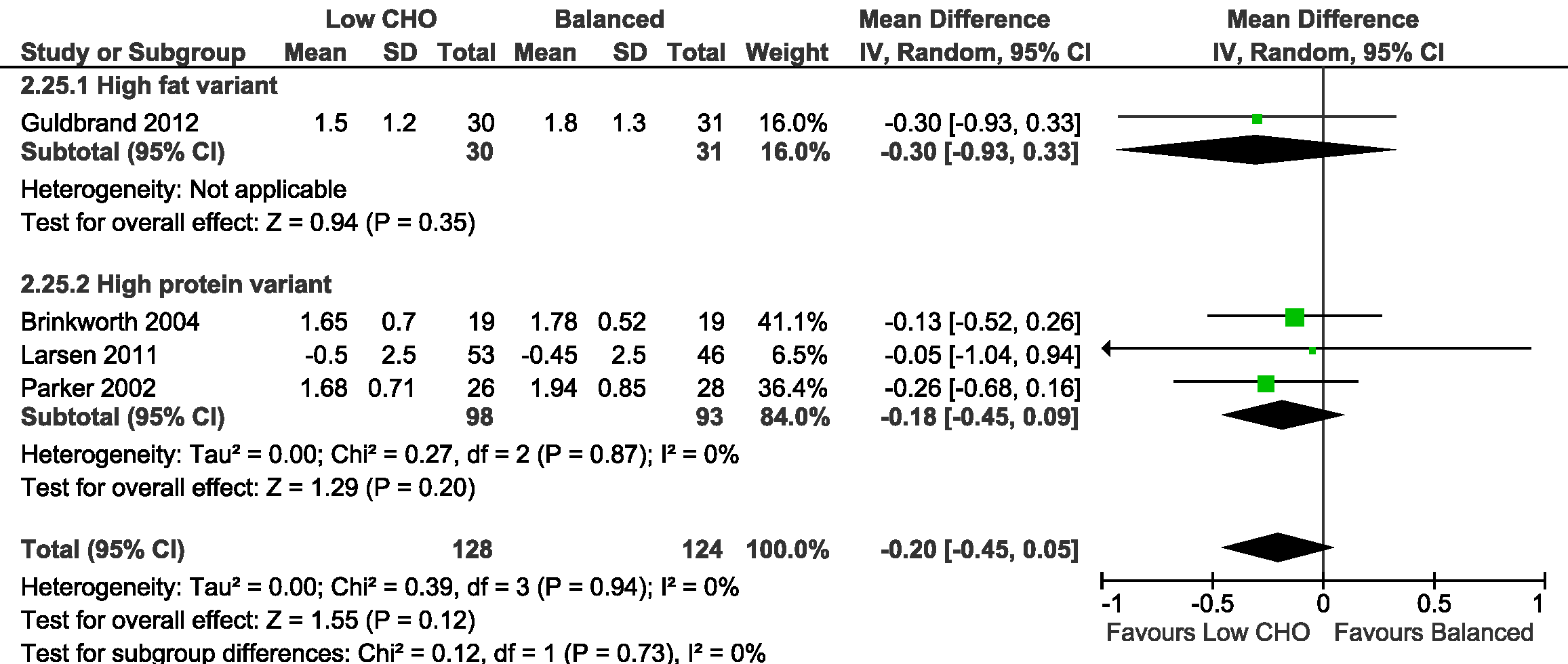


Figure S3Q: Forest plot of low carbohydrate versus balanced diets in overweight and obese adults with type 2 diabetes mellitus for triglycerides (mmol/L) at three to six months


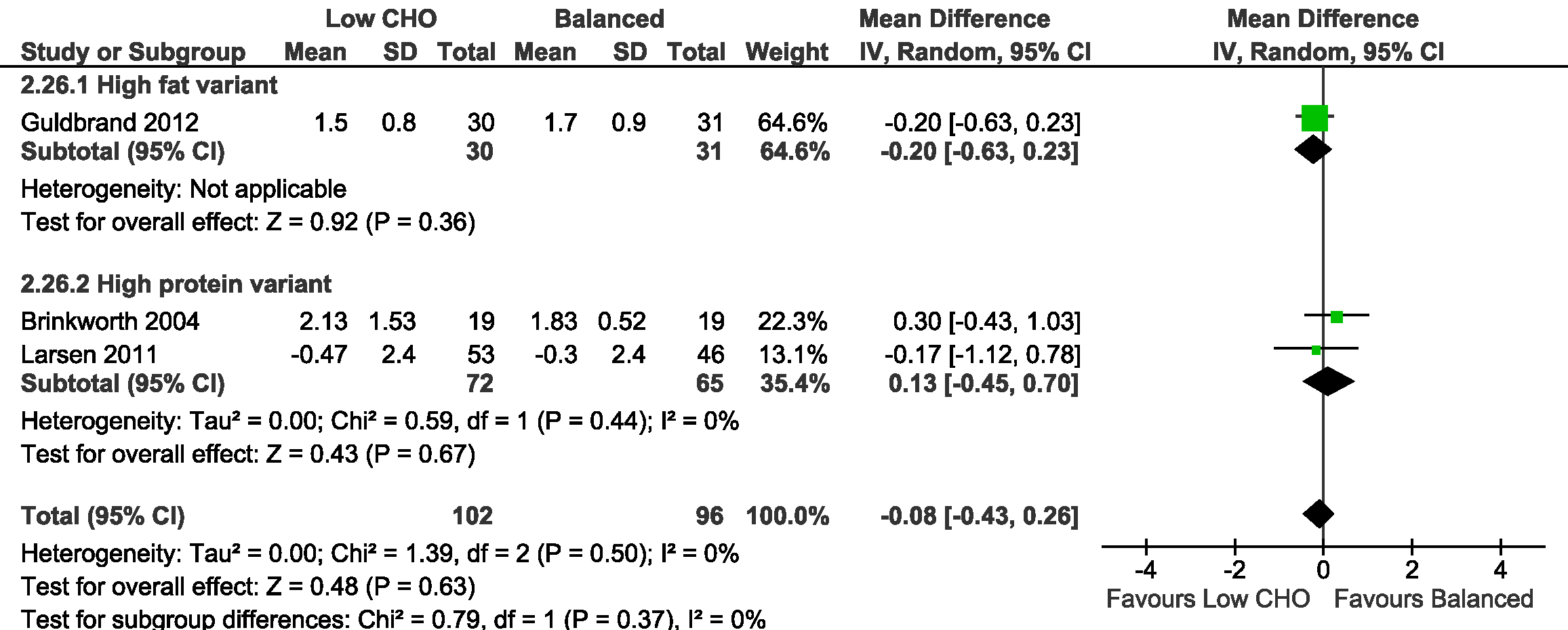


Figure S3R: Forest plot of low carbohydrate versus balanced diets in overweight and obese adults with type 2 diabetes mellitus for triglycerides (mmol/L) at one to two years
